# Clinical and biological significance of circulating miRNAs in chronic pancreatitis patients undergoing total pancreatectomy with islet autotransplantation

**DOI:** 10.1002/ctm2.1434

**Published:** 2023-10-17

**Authors:** Srividya Vasu, Giovanna Saracino, Carly M. Darden, Kenjiro Kumano, Yang Liu, Michael C. Lawrence, Bashoo Naziruddin

**Affiliations:** ^1^ Islet Cell Laboratory Baylor Scott and White Research Institute Dallas Texas USA; ^2^ Baylor Simmons Transplant Institute Baylor University Medical Center Dallas Texas USA; ^3^ Department of Gastroenterological Surgery Okayama University Graduate School of Medicine Dentistry and Pharmaceutical Sciences Okayama Japan; ^4^ The University of Texas Southwestern Medical Center Dallas Texas USA

**Keywords:** biomarkers, chronic pancreatitis, circulating miRNAs, islet autotransplantation

## Abstract

**Background:**

Specific microRNAs (miRNAs) were elevated in chronic pancreatitis (CP) patients during islet infusion after total pancreatectomy (TPIAT). We aimed to identify circulating miRNA signatures of pancreatic damage, predict miRNA‐mRNA networks to identify potential links to CP pathogenesis and identify islet isolation and transplantation functional outcomes.

**Methods:**

Small RNA sequencing was performed to identify distinct circulating miRNA signatures in CP. Plasma miRNAs were measured using miRCURY LNA SYBR green quantitative real‐time polymerase chain reaction assays. Correlation analyses were performed using R software. The miRNA target and disease interactions were determined using miRNet and the miRNA enrichment and annotation tool.

**Results:**

Alterations were found in circulating miRNAs in CP patients compared to healthy controls. Further studies were conducted on 12 circulating miRNAs enriched in the pancreas, other tissues and other diseases including cancer and fibrosis. Approximately 2888 mRNAs in the pancreas were their targets, demonstrating interactions with 76 small molecules. Three miRNAs exhibited interactions with morphine and five exhibited interactions with glucose. The miRNA panel targeted 22 genes associated with pancreatitis. The islet‐specific, acinar cell‐specific and liver‐specific miRNAs were elevated at 6 h after islet infusion and returned to baseline levels 3 months after TPIAT. Circulating levels of miRNAs returned to pre‐transplant levels 1‐year post‐transplant. Circulating miRNAs measured before and 6 h after islet infusion were directly or inversely associated with metabolic outcomes at 3 and 6 months post‐transplant.

**Conclusions:**

miRNAs may contribute to CP pathogenesis, and elevated circulating levels may be specific to pancreatic inflammation and fibrosis, warranting further investigation.

## Graphical abstract

Circulating microRNAs may exert biological functions in the pancreas during the pathogenesis of chronic pancreatitis. Circulating miRNAs may be predictors of clinical outcomes after islet autotransplantation.

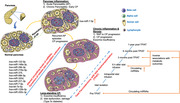

## 1 BACKGROUND

Chronic pancreatitis (CP), a pathologic, progressive fibroinflammatory syndrome of the pancreas, is characterized by chronic abdominal pain that significantly affects the quality of life.^1^ In patients with refractory CP, total pancreatectomy with islet autotransplantation (TPIAT) improves pain and quality of life and prevents brittle diabetes.^2^, ^3^ TPIAT is considered only after other surgical interventions have failed.^2^ Clinical practitioners face challenges in determining the timing of surgical interventions due to the disease's unpredictable and highly varied clinical course of the disease.^4^, ^5^ Although currently available diagnostic tests provide insights into morphological abnormalities and exocrine and endocrine function, they do not provide a clear picture of disease progression, the extent of pancreas inflammation, fibrosis, islet stress and damage before an irreversible loss of function.^4^, ^5^, ^6^ Most importantly, when exocrine pancreatic insufficiency and/or endocrine dysfunction are diagnosed, the damage caused by persistent inflammation and fibrotic replacement of pancreatic parenchyma is irreversible. Even if endocrine function is intact, an inflamed and fibrotic pancreas may affect islet isolation outcomes negatively. As optimal islet yield is critical in achieving insulin independence post‐TPIAT,^7^ knowledge of disease progression will help in determining the timing of surgery. After TPIAT, routine diagnostic tests cannot predict islet stress and damage before islet graft failure. Thus, there is an urgent need to develop metrics to determine the status of pancreas inflammation, fibrosis and islet damage.

Circulating microRNAs (miRNAs) are ideal candidates as biomarkers of pancreatic inflammation and islet damage. They are stable in circulation and can be detected easily using polymerase chain reaction (PCR) technologies. Islet stress and damage‐specific miRNAs (*hsa‐miR‐375*, *hsa‐miR‐148a‐3p*, *hsa‐miR‐29b‐3p*, *hsa‐miR‐216a‐5p* and *hsa‐miR‐200c‐3p*) were elevated in islet culture media and circulation during islet infusion in TPIAT patients.^8^, ^9^, ^10^, ^11^ Other studies have reported elevated levels of circulating miRNAs in patients with acute or chronic pancreatitis.^12^, ^13^, ^14^, ^15^ Further analysis of circulating miRNA profiles in CP patients is needed to identify key miRNA signatures with excellent diagnostic potential. As miRNAs are crucial for post‐transcriptional regulation of gene expression, they may play important roles in initiating and perpetuating pancreatic inflammation and fibrosis.^16^ The involvement of miRNAs in activating rat pancreatic stellate cells, which play a central role in fibrosis, was reported previously.^17^ A systematic review and meta‐analysis of miRNA–mRNA regulation networks in CP revealed *hsa‐miR‐324‐5p* and *NOTCH3* (neurogenic locus notch homolog protein 3)*, COX5A* (cytochrome C oxidase subunit 5a)*, THBS1* (thrombospondin 1) and *KARS* (lysyl‐tRNA synthetase) as high‐risk markers with high prediction accuracy.^18^ Further investigations of miRNA–mRNA networks in the pancreas and their contributions to the pathogenesis of CP are warranted.

Our objectives are to (1) identify differentially altered circulating miRNAs in CP; (2) identify the miRNA–mRNA networks in the pancreas; and (3) identify associations with metabolic outcomes before and after TPIAT. In this study, we profiled circulating miRNAs in CP patients compared to healthy controls using small RNA sequencing. We investigated the potential interactions of differentially expressed miRNAs in circulation in CP with mRNA networks in the pancreas. We further assessed whether the selected miRNAs targeted genes identified as associated with risk for pancreatitis using publicly available datasets (genome‐wide association studies). We also investigated the associations of the selected miRNA candidates with clinical measures before and after TPIAT.

## 2 MATERIAL & METHODS

### 2.1 Study design

This study of CP patients is based on an institutional review board–approved protocol (#010‐150) and informed consent. To analyse differentially expressed miRNAs in circulation in CP patients, plasma samples were collected from CP patients (*n* = 18, age > 18 and <55, any sex, race, aetiology and ethnicity) before TPIAT. Healthy donor plasma samples (n = 6, age > 18 and <55, any sex, race, ethnicity, no medications, body mass index < 26 kg/m2) were obtained from the Baylor University Medical Center biobank. To study associations of selected circulating miRNA candidates with clinical measures, we included patients (n = 40) of any age, sex, race, or ethnicity who had stored plasma samples before TPIAT, at 6 h after islet infusion and at 90 and 365 days after TPIAT. Patients diagnosed with other pancreatic diseases, including cancer, were excluded. Detailed information on the patient and clinical data collected is provided in Supporting Information. Blood was collected in ethylenediaminetetraacetic acid tubes, and plasma was separated, aliquoted and stored frozen at −80°C until further analysis.

### 2.2 Plasma RNA extraction

Cell‐free and exosomal RNA were extracted using the miRNeasy Serum/Plasma advanced kit (Qiagen) following the manufacturer's instructions. After stabilization using MS2 RNA (bacteriophage MS2, Millipore Sigma), spiking with Unisp6 (Qiagen) and lysis, plasma proteins were removed. After binding nucleic acids to the spin column and washing, miRNA was eluted in nuclease‐free water containing an RNAse inhibitor. MS2 RNA, UniSp6 and RNAse inhibitors were avoided for small RNA sequencing.

### 2.3 Small RNA sequencing analysis

Small RNA integrity and quality were assessed using an Agilent 2100 Bioanalyzer. To profile circulating miRNAs, a miRNA library was constructed using the TruSeq Small RNA Library Prep Kit (Illumina Inc.) following the manufacturer's instructions. Following quality control, the libraries were sequenced using a HiSeq 2500 Genome Analyzer (Illumina Inc.), with a sequencing depth of 2 million reads per sample and a read length of 50 bp. Fastq files were generated from base‐calling files using Illumina bcl2fastq2 software for data analysis. After trimming 3′‐adapter sequences using Cutadapt,^19^ reads of length less than 12 nucleotides were excluded. The resulting trimmed reads were aligned to a publicly available database containing published human miRNA sequences and annotations (miRbase, release 22) using the quantifier.pl module from miRDeep2.^20^ The count data were analysed using DeSeq2 for normalization and differential analysis.^21^ For principal component analysis and hierarchical clustering, the heatmap function package for non‐negative matrix factorization was used (R software, version 3.6.3).^22^ miRNAs with *p* < .05 were considered to be differentially expressed.

### 2.4 Absolute quantification of circulating miRNAs

After RNA extraction from plasma and miRNA (2 μL/sample) conversion to cDNA (miRCURY LNA RT kit, Qiagen), quantitative real‐time PCR (qPCR, cDNA dilution 1:40) was performed (QuantStudio 7 Flex real‐time PCR system, Applied Biosystems) using miRCURY LNA miRNA PCR assay system following the manufacturer's instructions. A standard curve using specific miRNA mimics (miRNA mimics, Qiagen) and melting curve analysis were included in all reactions. The UniSp6 cycle threshold value (±0.2) was used as a quality control for inclusion in the data analysis. Patient information was protected by relabeling all samples and blinding the investigator. All samples were assayed in triplicate. QuantStudio™ 7 Flex software was used for data analysis (Applied Biosystems).

### 2.5 Overrepresentation analysis

Overrepresentation analysis was performed using the miRNA Enrichment Analysis and Annotation Tool, miEAA (version 2.0).^23^ The following categories were explored: pathways (Kyoto Encyclopaedia of Genes and Genomes; KEGG), exRNA forms (miRandola), target genes (miRTarBase), diseases (miRWalk), organs (miRWalk), localisation (RNALocate) and expressed in tissue (TissueAtlas). Benjamini–Hochberg adjustment (false discovery rate [FDR]) (independent adjustment of *p* values for each category, significance level at .05) was used for multiple comparisons.

### 2.6 miRNA–mRNA network analysis

Interactions of miRNAs with mRNAs in the pancreas (464 datasets) or small molecules were assessed using miRNet (version 2.0).^24^ To identify miRNA interactions with genes associated with a risk for pancreatitis, we compiled a list of predicted targets of the selected miRNAs using TargetScan (version 8.0).^25^ We searched the genome‐wide association studies catalogue (National Human Genome Research Institute—European Bioinformatics Institute, NHGRI‐ EBI) for genes associated with risk for pancreatitis. Based on 25 published studies, 187 variants (112 mapped genes) were associated with pancreatitis. We compared these lists of mRNA targets of miRNAs and pancreatitis‐associated genes (13 lists) using a web‐based “multiple list comparator” tool (molbiotools.com)^26^ to identify potential interactions with genes associated with risk for pancreatitis.

### 2.7 Statistical analysis

All analyses were performed using R software (version 4.2.2)^22^ and Bioconductor 3.16.^27^ Raw miRNA data were transformed to the common log (log to base 10) scale for all analyses. Associations of log_10_miRNA levels with measures on continuous scales (e.g., C‐peptide or islet‐yield measures) were determined using linear regression. The data were corrected for multiple comparisons for computing FDR‐adjusted *p*‐values with and without bootstrapping (empirical distributions, bootstrapped resampling with 1000 replications). In each comparison, a clinical parameter, a time point and 12 miRNAs were considered, and *p*‐values were averaged over all replications. GraphPad PRISM (version 9.0) was used for receiver operating characteristic curve analysis. A *p‐*value of < .05 was considered statistically significant. For multiple comparisons, a *p*‐value of < .1 was considered statistically significant.

## 3 RESULTS

### 3.1 Distinct circulating miRNA profiles of CP patients

**FIGURE 1:**
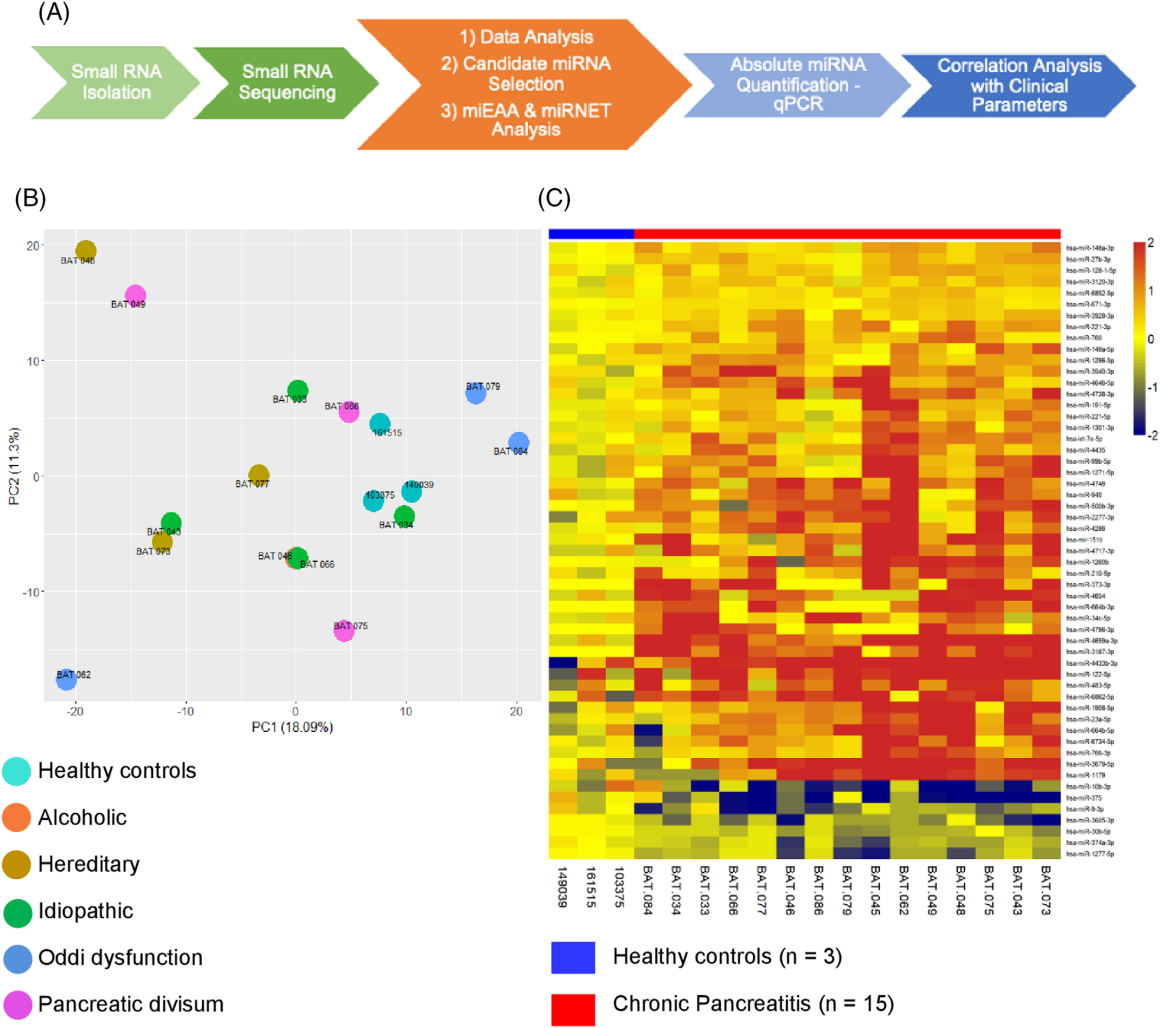
Circulating miRNA profiles in chronic pancreatitis. (A) Experimental design. Plasma miRNAs were isolated and subjected to small RNA sequencing analysis. (B) Principal component analysis plot. (C) Heatmap representation of circulating miRNA sequencing data (differential expression analysis based on the negative binomial model) from patients with chronic pancreatitis and healthy controls. Colour codes for fold change are indicated in the heat map.

The experimental design for the study is provided in Figure 1A. To profile circulating miRNAs in patients with CP, we isolated small RNAs from plasma collected from patients (n = 18) admitted for TPIAT and normal healthy donors (n = 6) and performed small RNA‐seq analyses. Quality control revealed that healthy donor samples (n = 3) exhibited low levels of circulating miRNAs, leading to insufficient reads (<2 million reads) and, hence, were excluded from further analyses. Principal component analyses revealed that the circulating small RNA profiles of healthy donors were clustered, while those of patients were variable (Figure 1B). In the patient group, three samples were excluded because of high variability (principal component analysis), poor library quality and insufficient reads for analyses (<2 million reads). A heatmap showing the differential circulating miRNAs in patients with CP compared to healthy donors is depicted in Figure 1C. Small RNA‐seq analysis revealed 803 miRNAs in circulation, of which 53 were significantly expressed in patients with CP (*p* < .05, Figure 1C). When adjusted for multiple comparisons, these did not reach statistical significance (Table 1).

**TABLE 1:** Differentially expressed circulating miRNAs in patients with chronic pancreatitis.*

Circulating miRNA	Base mean	log_2_FC	FC	*p* Value	*p*_adj_ Value
**Elevated miRNAs in circulation**					
hsa‐miR‐148a‐3p	33539.75	0.58	1.50	.020	.69
hsa‐miR‐99b‐5p	17019.20	1.27	2.42	.035	.72
hsa‐miR‐221‐3p	10168.09	0.70	1.63	.006	.58
hsa‐miR‐122‐5p	7163.39	2.07	4.20	.001	.55
hsa‐let‐7e‐5p	4804.17	1.01	2.01	.011	.69
hsa‐miR‐1301‐3p	1033.59	0.68	1.61	.035	.72
hsa‐miR‐148a‐5p	826.59	0.83	1.77	.006	.58
hsa‐miR‐1271‐5p	400.51	0.96	1.95	.033	.72
hsa‐miR‐4286	222.97	1.15	2.22	.004	.58
hsa‐miR‐766‐3p	163.77	0.89	1.85	.030	.72
hsa‐miR‐4435	85.87	0.79	1.73	.020	.69
hsa‐miR‐1908‐5p	78.00	1.42	2.67	.015	.69
hsa‐miR‐1296‐5p	73.54	0.72	1.65	.006	.58
hsa‐miR‐1260b	66.01	1.52	2.87	.011	.69
hsa‐miR‐23a‐5p	65.31	1.21	2.31	.006	.58
hsa‐miR‐4433b‐3p	58.36	2.04	4.12	.001	.55
hsa‐miR‐940	27.84	1.17	2.25	.017	.69
hsa‐miR‐760	27.32	0.73	1.66	.051	.72
hsa‐miR‐483‐5p	23.22	1.45	2.73	.015	.69
hsa‐miR‐34c‐5p	13.05	1.71	3.28	.021	.69
**Reduced miRNAs in circulation**					
hsa‐miR‐375	1383.80	−1.32	0.40	.04	.72
hsa‐miR‐145‐5p	221.16	−0.90	0.54	.03	.72
hsa‐miR‐3605‐3p	35.78	−0.85	0.56	.03	.72
hsa‐miR‐10b‐3p	10.83	−1.43	0.37	.05	.72

* Small RNA was isolated from patient plasma samples and subjected to small RNA sequencing. Differentially expressed circulating miRNAs with high base mean in chronic pancreatitis patients (n = 15) compared to healthy controls (n = 3) are listed. Out of six healthy controls included in the study, three samples did not contain measurable levels of circulating miRNAs and were excluded from the study. FC, fold change; *p*_adj_, *p*‐value adjusted for multiple comparisons.

Of the differentially expressed miRNAs, 39 miRNAs were markedly elevated and 5 miRNAs were reduced in CP patients compared to healthy donors (fold change [FC] > 1.5 or 0.6 < FC, *p* < .05). Of these, 24 miRNAs (20 miRNAs elevated and 4 downregulated) were shortlisted based on base mean > 10 (Table 1). For further follow‐up studies, we selected *hsa‐miR‐99b‐5p, hsa‐miR‐148a‐3p, hsa‐miR‐122‐5p, hsa‐miR‐221‐3p* and *hsa‐let‐7e‐5p* based on base mean > 4000, FC > 1.5 and *p* value < .05. Of the downregulated miRNAs, we selected *hsa‐miR‐375* based on base mean > 1000, FC < 0.5 and *p* value < .05. Additionally, we included the following miRNAs of interest to our panel for further analysis: *hsa‐miR‐7‐5p*,^13^ *hsa‐miR‐216a‐*5p,^28^ *hsa‐miR‐200c‐3p* and *hsa‐miR‐29b‐3p*,^10^ *hsa‐miR‐320d*,^15^ and *hsa‐miR‐125b‐5p*.^29^ We included these miRNAs in our analysis because they were previously differentially altered in circulation in CP or AP.

### 3.2 Tissue expression and localization of selected miRNA candidates

**FIGURE 2:**
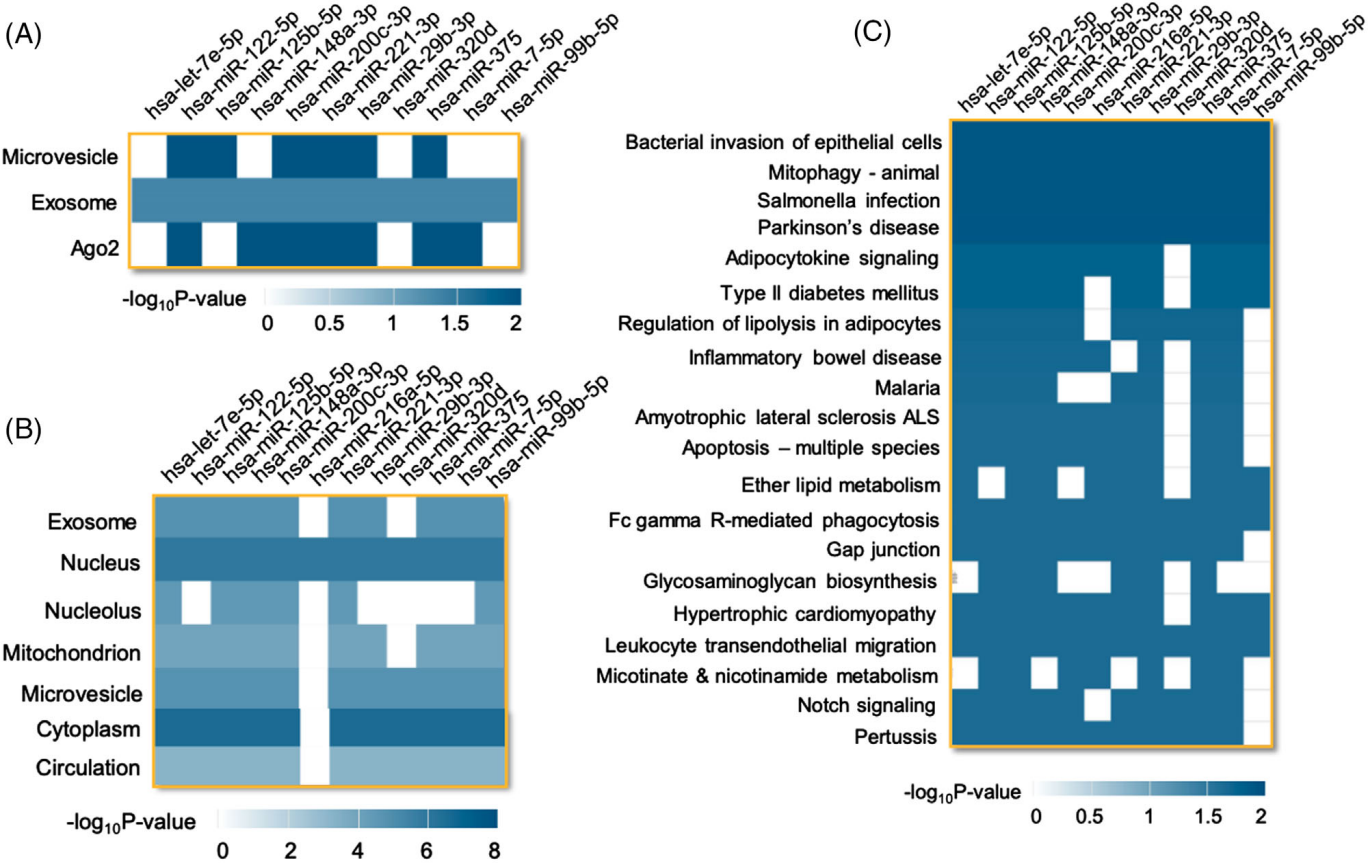
Tissue expression and localization of selected miRNA candidates and overrepresentation in KEGG pathways. Selected miRNAs were input into miRNA Enrichment and Annotation Tool software for overrepresentation analysis in (A) association in circulation; (B) cellular localization; and (C) KEGG pathways. Heat maps illustrate ‐log_10_(*p* value) for corresponding miRNAs.

We performed overrepresentation analysis using the miEAA^23^ to assess expression in diseases, organs and tissues and their cellular localization (Figure 2). Of the 12 selected miRNA candidates, 11 have been identified in extracellular vesicles called exosomes and 6 have been identified in microvesicles. Most importantly, 7 of these miRNA candidates have been associated with Argonaut 2 (Ago2) complexes in circulation, forming functional miRNA‐mediated silencing complexes (miRISC/Ago2) (Figure 2A). Selected miRNA candidates have also been identified in the nucleus, and except for *hsa‐miR‐216a‐5p*, all miRNA candidates have been observed in microvesicles, cytoplasm and circulation (Figure 2B). Supporting Information Table S1 lists tissues enriched with the selected miRNA candidates (miRNet version 2.0, function explorer tool).^24^ Interestingly, except *hsa‐let‐7e‐5p*, all miRNA candidates were significantly enriched in the pancreas (Supporting Information Table S1). These miRNAs were overrepresented in several pathways, including bacterial invasion of epithelial cells, Parkinson's disease pathogenesis, type 2 diabetes, apoptosis and notch signalling (Figure 2C) (KEGG). These selected miRNA candidates have also been implicated in several diseases (Supporting Information Table 2).

### 3.3 miRNA networks in the pancreas and possible links to CP

**FIGURE 3:**
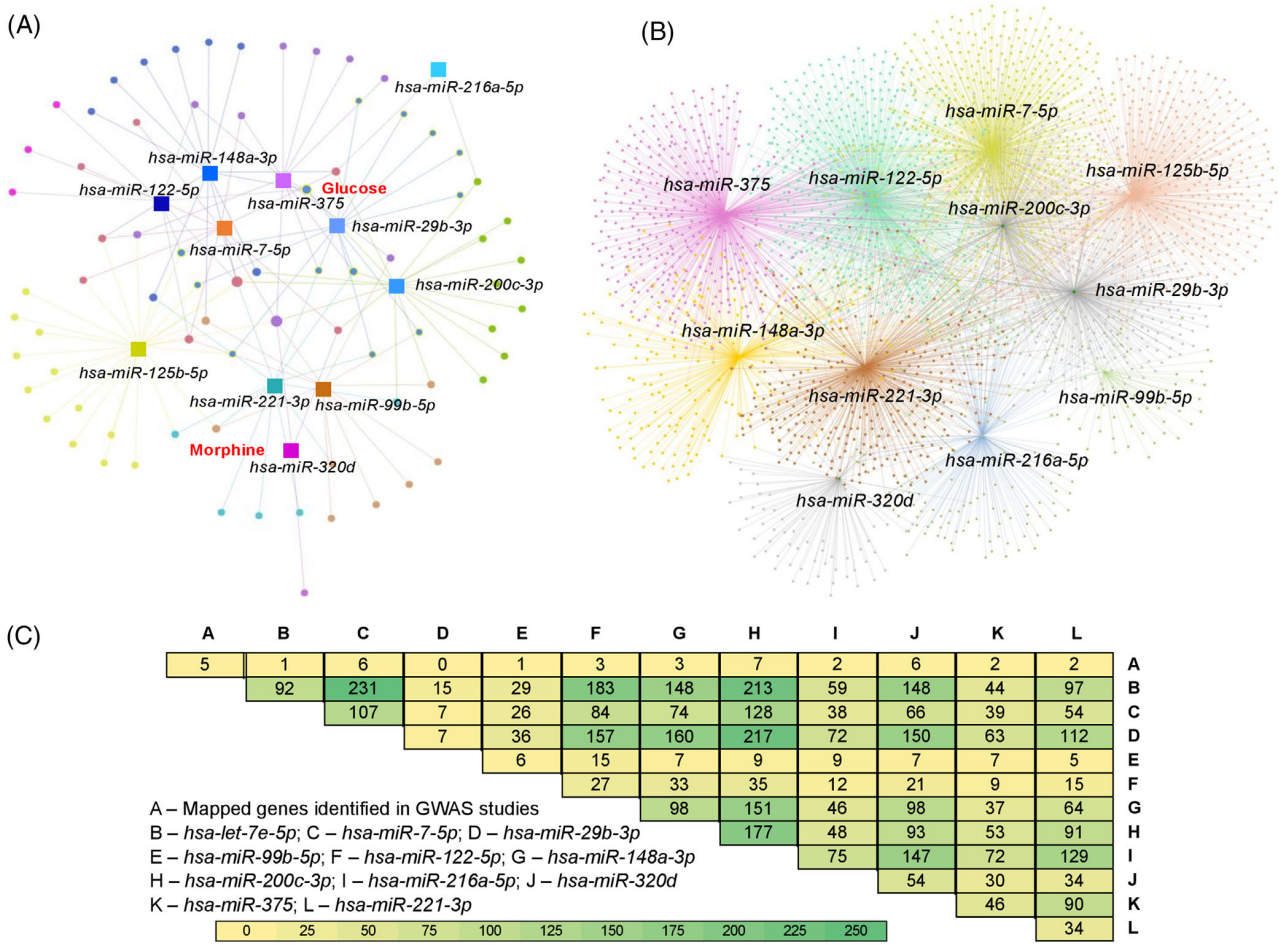
miRNA‐mRNA networks in the pancreas. (A) Clusters reveal distinct miRNA‐compound interaction networks in the pancreas based on expression data available in the literature. (B) Clusters reveal distinct miRNA‐mRNA interaction networks in the pancreas based on expression data available in the literature. (C) Jaccard similarity plot showing common targets of selected miRNA candidates in the pancreas and genes identified as associated with risk for pancreatitis.

**FIGURE 4:**
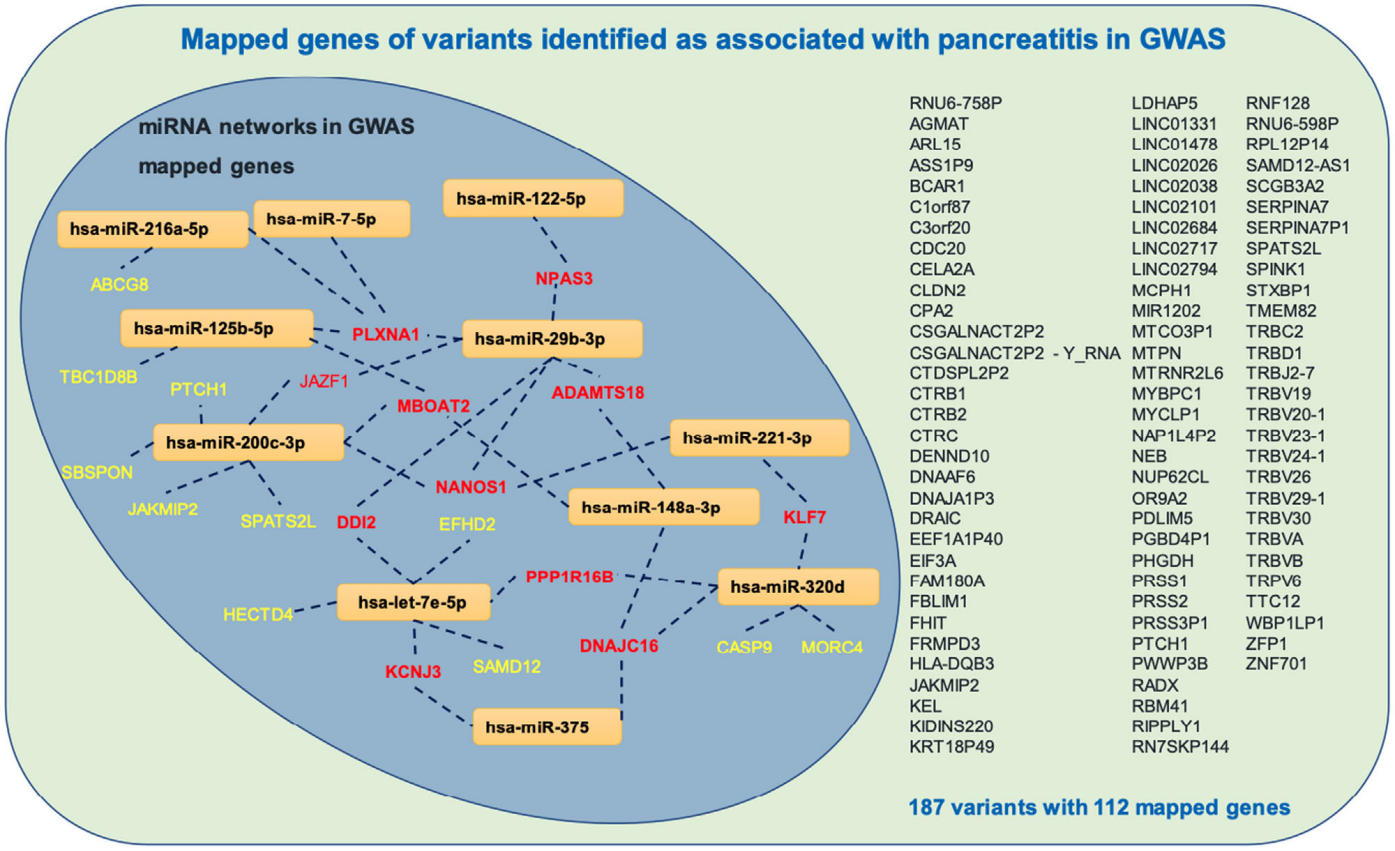
miRNA networks with genes identified as associated with risk for pancreatitis. A total of 112 mapped genes corresponding to 187 variants were identified as associated with risk for pancreatitis in genome‐wide association studies. Of these, predicted interactions of miRNAs with genes are shown. Genes with 2 or more interactions with miRNAs are depicted in red.

We analysed miRNA:mRNA/small molecule networks in the pancreas to identify key players in the pathogenesis of CP using miRNet (version 2.0)^23^ (Figure 3A, B). Exploration of miRNA interactions with small molecules revealed 72 compounds, including glucose, morphine, vitamin D3, activin A and metformin, with 152 interactions (Figure 3A). In the pancreas, our analyses revealed 7938 genes as targets of 11 out of 12 miRNAs, with 16 222 interactions (Figure 3B). To further identify miRNA networks with genes associated with a risk for pancreatitis, we compiled lists of predicted targets of these miRNAs using TargetScan (Version 8.0).^25^ We then compared these lists to 187 variants (mapping to 112 genes) identified as associated with pancreatitis in genome‐wide association studies using the NHGRI‐EBI catalogue. The commonalities between genes associated with pancreatitis and miRNAs are provided in Figure 3C. These possible miRNA‐gene networks in the context of pancreatitis are depicted in Figure 4. Genes with at least two interactions with selected miRNA candidates are indicated in red.

### 3.4 Pre‐ and post‐operative patient characteristics

To determine whether levels of selected circulating miRNA profiles are altered in patients after TPIAT, we included 40 patients from a total of 200 patients who had follow‐up plasma samples and data available at admission for TPIAT, 6 h after islet infusion and 3 months and 1 year after TPIAT. We included the 15 patients from our small RNA sequencing study in these follow‐up studies. Pre‐ and post‐operative characteristics of the selected patient cohort are provided in Tables 2 and 3, respectively. Table 2 includes patient demographics, disease duration and aetiology of pancreatitis, metabolic profile and information on islet isolation. Table 3 provides functional outcomes at 3, 6 months and 1 year after transplantation including haemoglobin A1c, glycosylated haemoglobin (HbA1c), fasting C‐peptide, glucose, plasma glucagon levels and insulin usage. At 6 months after TPIAT, plasma glucagon levels were not available.

**TABLE 2:** Preoperative patient characteristics.

Parameter	n	Median (IQR)/n (%)
Age at transplant (years)	40	44 (32, 48)
Female	30	30 (75%)
Male	10	10 (25%)
Body mass index (kg/m^2^)	40	26.7 (21.5, 29.8)
Diabetes	40	4 (10%)
Past history of pancreas operation	40	8 (20%)
Duration of symptoms of disease	40	4.2 (2.9, 9.0)
Aetiology: Autoimmune	40	4 (10%)
Genetic	40	6 (15%)
Idiopathic	40	17 (42%)
Obstructive	40	9 (22%)
Toxic	40	4 (10%)
Haemoglobin A1c (%)	40	5.50 (5.18, 6.00)
Glucose (fasting, mg/dL)	39	98 (88, 112)
Glucose (2 h post‐prandial, mg/dL)	38	165 (117, 204)
C‐peptide (fasting, ng/mL)	40	1.70 (0.90, 2.85)
Plasma glucagon (fasting, pg/mL)	40	16.6 (13.1)
Total islet yield (islet equivalents)	40	363,952 (278,936, 502,179)
Islet yield (IE/g of pancreas)	40	4846 (3204, 6215)
Total tissue volume (mL)	40	10 (7, 17)

**TABLE 3:** Metabolic outcomes at 3, 6 and 12 months after total pancreatectomy with islet autotransplantation.*

Time after TPIAT	Characteristic	N	Median (IQR)
3 months	Haemoglobin A1c (%)	38	6.15 (5.50, 6.77)
	C‐peptide (fasting, ng/mL)	35	1.10 (0.65, 2.10)
	Glucose (fasting, mg/dL)	37	111 (95, 138)
	Insulin (U/kg)	40	0.12 (0.00, 0.24)
	Plasma glucagon (fasting, pg/mL)	40	23.8 (21)
	Insulin independence	11	
6 months	Haemoglobin A1c (%)	34	6.35 (5.62, 6.68)
	C‐peptide (fasting, ng/mL)	28	1.65 (0.80, 2.70)
	Glucose (fasting, mg/dL)	33	111 (93, 154)
	Insulin (U/kg)	38	0.09 (0.00, 0.23)
	Plasma glucagon (fasting, pg/mL)	NA	
	Insulin independence	17	
1 year	Haemoglobin A1c (%)	38	6.60 (5.93, 7.47)
	C‐peptide (fasting, ng/mL)	34	1.30 (0.62, 2.00)
	Glucose (fasting, mg/dL)	37	108 (86, 154)
	Insulin (U/kg)	39	0.10 (0.00, 0.32)
	Plasma glucagon (fasting, pg/mL)	40	28.8 (20)
	Insulin independence	18	

* Follow‐up data were not available for all patients included in the study. NA, data not available.

### 3.5 The ability of hsa‐miR‐125b‐5p and hsa‐122‐5p to distinguish CP patients from healthy donors

**FIGURE 5:**
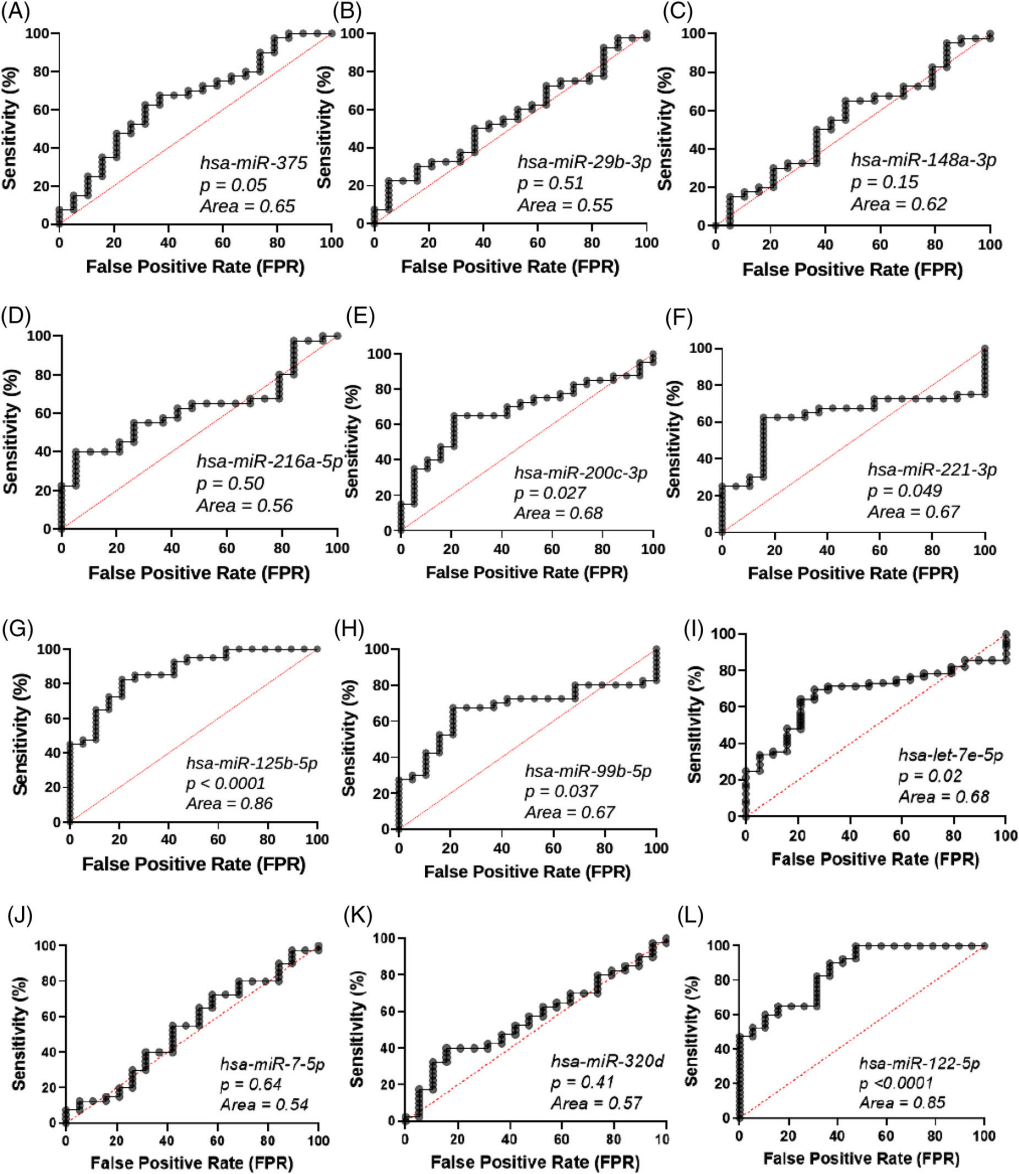
Diagnostic accuracy of circulating miRNAs. Receiver operating characteristic curves show the sensitivity and specificity of circulating miRNAs in discriminating patients with chronic pancreatitis from healthy controls for (A) *hsa‐miR‐375*; (B) *hsa‐miR‐29b‐3p*; (C) *hsa‐miR‐148a‐3p*; (D) *hsa‐miR‐216a‐5p*; (E) *hsa‐miR‐200c‐3p*; (F) *hsa‐miR‐221‐3p*; (G) *hsa‐miR‐125b‐5p*; (H) *hsa‐miR‐99b‐5p*; (I) *hsa‐let‐7e‐5p*; (J) *hsa‐miR‐7‐5p*; (K) *hsa‐miR‐320d*; (L) *hsa‐miR‐122‐5p*. The area under the curve and *p* values are indicated in the graphs.

For receiver operating characteristic analysis, we compared the circulating miRNA levels of 19 healthy donor samples (10 female and 9 male donors aged 26−54; Supporting Information Table S3) and 40 CP patient plasma samples collected before TPIAT. We observed that *hsa‐miR‐375, hsa‐miR‐221‐3p, hsa‐miR‐200c‐3p, hsa‐miR‐125b‐5p, hsa‐miR‐99b‐5p, hsa‐let‐7e‐5p* and *hsa‐miR‐122‐5p* could distinguish between healthy donors and CP patients significantly (Figure 5). Of these, *hsa‐miR‐122‐5p* and *hsa‐miR‐125b‐5p* exhibited higher area under the curve (AUC) scores (AUC = 0.86 and 0.85, respectively, *p* < .0001, Figure 5G, L), demonstrating good discrimination from healthy donors.

### 3.6 Varied profiles of circulating miRNAs before and after TPIAT

**FIGURE 6:**
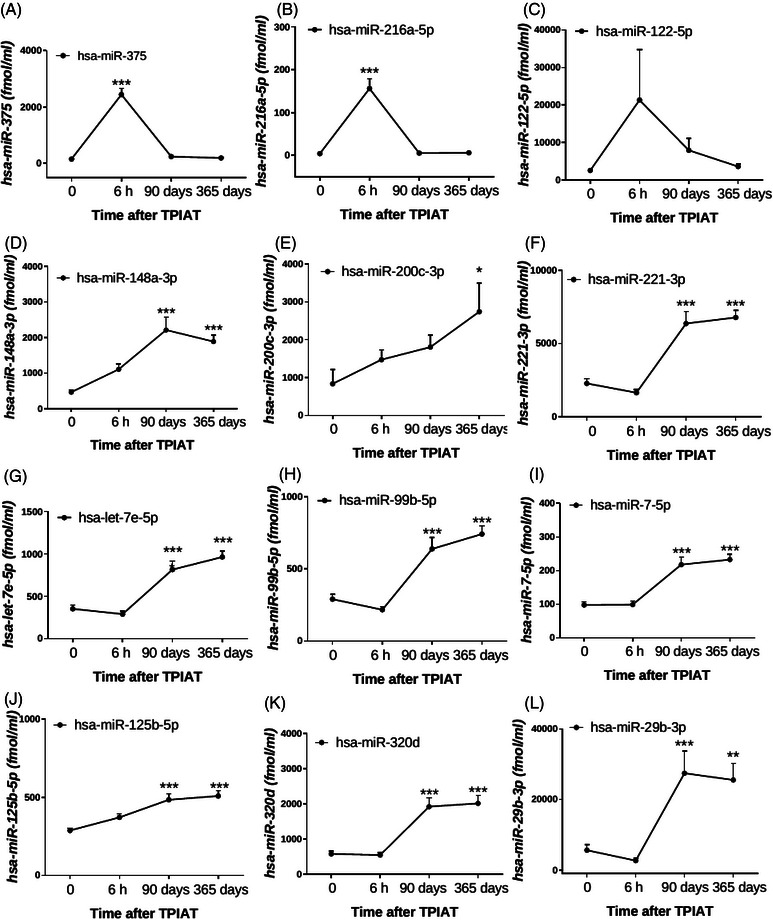
Circulating miRNA profiles before and after TPIAT. The levels (fmol/mL) of circulating miRNAs before (time point 0) and after TPIAT (6 h, 90 days and 365 days) are shown for (A) *hsa‐miR‐375*; (B) *hsa‐miR‐216a‐5p*; (C) *hsa‐miR‐122‐5p*; (D) *hsa‐miR‐148a‐3p*; (E) *hsa‐miR‐200c‐3p*; (F) *hsa‐miR‐221‐3p*; (G) *hsa‐let‐7e‐5p*; (H) *hsa‐miR‐99b‐5p*; (I) *hsa‐miR‐7‐5p*; (J) *hsa‐miR‐125b‐5p*; (K) *hsa‐miR‐320d*; (L) *hsa‐miR‐29b‐3p*. Values are means ± SD (n = 40). *p < .05, **p < .01 and ***p < .001 compared to baseline levels before TPIAT (time point 0).

To assess levels of circulating miRNAs before and after TPIAT, we performed a time‐course analysis of selected circulating miRNAs at admission, 6 h after islet infusion and 3 months and 1 year after TPIAT. Absolute levels of circulating miRNAs (fmol/mL) are provided in Figure 6. Circulating levels of *hsa‐miR‐375* (islet‐specific, *p* < .001, Figure 6A), *hsa‐miR‐216a‐5p* (acinar cell‐specific, *p* < .001, Figure 6B) and *hsa‐miR‐122‐5p* (liver‐specific, *p* < .001, Figure 6C) were elevated at 6 h after islet infusion. Interestingly, islet‐specific and acinar cell‐specific miRNA levels returned to baseline 3 months after TPIAT. At 3 months, although not significant, *hsa‐miR‐122‐5p* was slightly elevated but returned to baseline at 1 year after TPIAT, suggesting gradual improvement in liver inflammation after TPIAT. Levels of other circulating miRNAs gradually increased from 3 months and remained elevated at 1 year post‐TPIAT (*p* < .05, < .01, < .001, Figure 6).

### 3.7 Associations of pre‐TPIAT levels of circulating miRNAs with clinical parameters before and after TPIAT

**FIGURE 7:**
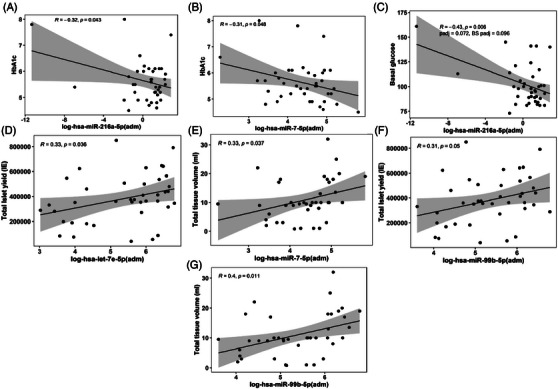
Association of pre‐TPIAT circulating miRNAs with pre‐TPIAT metabolic measures and islet isolation outcomes. Scatterplots show the correlations of pre‐TPIAT circulating miRNAs with pre‐TPIAT metabolic measures for (A) log‐*hsa‐miR‐216a‐5p* vs. haemoglobin A1c; (B) log‐*hsa‐miR‐7‐5p* vs. haemoglobin A1c; (C) log‐*hsa‐miR‐216a‐5p* vs. basal glucose; (D) log‐*hsa‐let‐7e‐5p* vs. total islet yield (IE); (E) log‐*hsa‐miR‐7‐5p* vs. total tissue volume (mL); (F) log‐*hsa‐miR‐99b‐5p* vs. total islet yield (IE); (G) log‐*hsa‐miR‐99b‐5p* vs. total tissue volume (mL). Pre‐TPIAT samples were taken at admission (adm). Pearson's correlation coefficient, *R* and *p* values (n = 40) are provided in the scatterplots.

**FIGURE 8:**
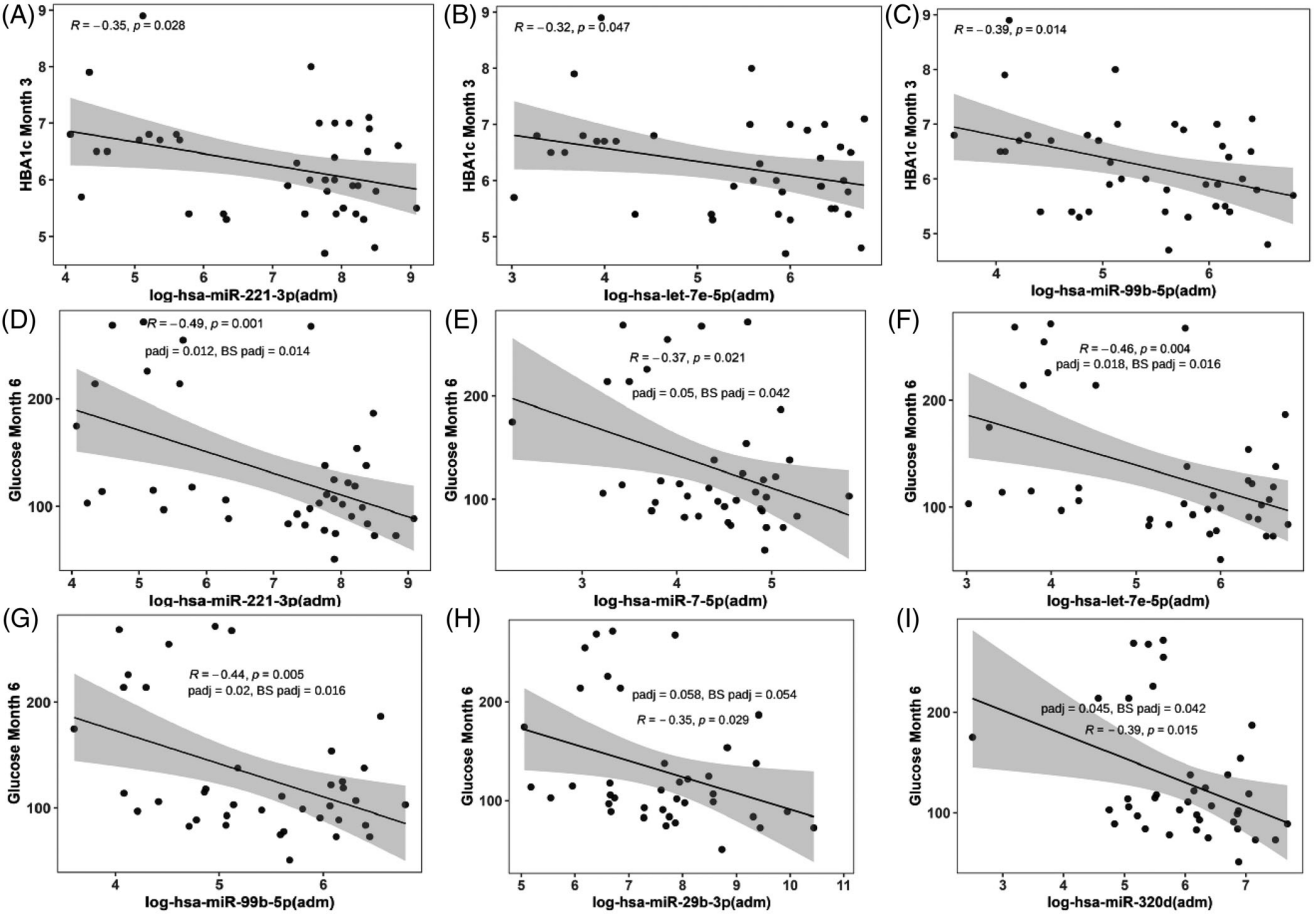
Association of pre‐TPIAT circulating miRNAs with metabolic measures after TPIAT. Scatterplots show the correlations of pre‐TPIAT circulating miRNAs with metabolic measures after TPIAT for (A) log‐*hsa‐miR‐221‐3p* vs. haemoglobin A1c month 3; (B) log‐*hsa‐let‐7e‐5p* vs. haemoglobin A1c month 3; (C) log‐*hsa‐miR‐99b‐5p* vs. haemoglobin A1c month 3; (D) log‐*hsa‐miR‐221‐3p* vs. glucose month 3; (E) log‐*hsa‐miR‐7‐5p* vs. glucose month 6; (F) log‐*hsa‐let‐7e‐5p* vs. glucose month 6; (G) log‐*hsa‐miR‐99b‐5p* vs. glucose month 6. (H) log‐*hsa‐miR‐29b‐3p* vs. glucose month 6. (I) log‐*hsa‐miR‐320d* vs. glucose month 6. Pre‐TPIAT samples were taken at admission (adm). Pearson's correlation coefficient, *R* and *p* values (n = 40) are provided in the scatterplots.

We performed correlation analyses to assess whether levels of circulating miRNAs measured at admission (pre‐TPIAT) were associated with preoperative patient characteristics, islet isolation and transplant outcomes. Circulating miRNAs were not associated with age, body mass index, or duration of disease symptoms. Levels of pre‐TPIAT log‐*hsa‐miR‐216a‐5p* (*R* = −.32, *p* = .043, Figure 7A) and log‐*hsa‐miR‐7‐5p* (*R* = −.31, *p* = .048, Figure 7B) were inversely associated with pre‐TPIAT HbA1c. Pre‐TPIAT log‐*hsa‐miR‐216a‐5p* was inversely associated with basal glucose (*R* = −.43, *p* = .006, Figure 7C), which remained significant after correcting for multiple comparisons (*p_adj_* = .072, BS *p_adj_* = .096). The level of pre‐TPIAT log‐*hsa‐let‐7e‐5p* was associated with total islet yield (*R* = .33, *p* = .036, Figure 7D), while pre‐TPIAT log‐*hsa‐miR‐7‐5p* was associated with total tissue volume (*R* = .33, *p* = .037, Figure 7E). The level of log‐*hsa‐miR‐99b‐5p* was associated with islet yield (*R* = .31, *p* = .05, Figure 7F) and total tissue volume (*R* = .4, *p* = .011, Figure 7G). Pre‐TPIAT levels of log‐hs*a‐miR‐221‐3p* (*R* = −.35, *p* = .028, Figure 8A), log‐*hsa‐let‐7e‐5p* (*R* = −.32, *p* = .047, Figure 8B) and log‐*hsa‐miR‐99b‐5p* (*R* = −.39, *p* = .014, Figure 8C) were inversely associated with HbA1c at 3 months. Pre‐TPIAT levels of log‐*hsa‐miR‐221‐3p* (*R* = −.49, *p* = .001, Figure 8D), log‐*hsa‐miR‐7‐5p* (*R* = −.37, *p* = .021, Figure 8E), log‐*hsa‐let‐7e‐5p* (*R* = −.46, *p* = .004, Figure 8F), log‐*hsa‐miR‐99b‐5p* (*R* = −.44, *p* = .005, Figure 8G), log‐*hsa‐miR‐29b‐3p* (*R* = −.35, *p* = .029) and log‐*hsa‐miR‐320d* (*R* = −.39, *p* = .015) were inversely associated with blood glucose levels at 6 months. These were significant after correction for multiple comparisons (Figure 8D–I).

Associations of circulating miRNAs 6 h after islet infusion with islet isolation characteristics and metabolic measures before and after TPIAT. Levels of log‐hsa‐miR‐375 (*R* = .41, *p* = .009) and log‐hsa‐miR‐216a‐5p (*R* = .32, p = .041) were measured at 6 h after islet infusion was associated with fasting C‐peptide. Levels of log‐hsa‐miR‐200c‐3p (*R* = −.48, *p* = .002) and log‐hsa‐miR‐7‐5p (*R* = −.36, *p* = .027) were inversely associated with 2 h post‐prandial glucose (Table 4). Level of log‐hsa‐miR‐375 measured at 6 h after islet infusion was associated with total islet yield (*R* = .55, *p* = .0001, *p*_adj_ = .001, BS *p*_adj_ = .002), tissue volume (*R* = .68, *p* < .0001, *p*_adj_ < .001, BS *p*_adj_ = .001),and islet particle number (*R* = .57, *p* < .0001, *p*_adj_ < .001, BS *p*_adj_ = .001). Level of log‐hsa‐miR‐216a‐5p measured at 6 h after islet infusion was associated with total islet yield (*R* = .36, *p* = .021, *p*_adj_ = .13, BS *p*_adj_ = .13), tissue volume (*R* = .57, *p* < .0001, *p*_adj_ < .001, BS *p*_adj_ = .001) and islet particle number (*R* = .37, *p* = .019, *p*_adj_ = .11, BS *p*_adj_ = .093). Level of log‐has‐miR‐148a‐3p was associated with tissue volume (*R* = .40, *p* = .012, *p*_adj_ = .048, BS *p*_adj_ = .041). Levels of log‐hsa‐miR‐375 (*R* = −.33, *p* = .046), log‐hsa‐miR‐200c‐3p (*R* = −.43, *p* = .007), log‐hsa‐miR‐221‐3p (*R* = −.37, *p* = .024), log‐hsa‐miR‐99b‐5p (*R* = −.35, *p* = .033), log‐hsa‐miR‐7‐5p (*R* = −.40, *p* = .013) and log‐hsa‐let‐7e‐5p (*R* = −.34, *p* = .037) measured at 6 h after islet infusion were inversely correlated with fasting glucose at 3 months after TPIAT (Table 4). The level of log‐hsa‐miR‐216a‐5p measured at 6 h after islet infusion was associated with fasting C‐peptide at 3 months post‐TPIAT (*R* = .35, *p* = .038, Table 4). The level of log‐hsa‐miR‐375 measured at 6 h after islet infusion was inversely associated with HbA1c at 3 months post‐TPIAT (*R* = −.39, *p* = .016, Table 4). Levels of log‐hsa‐miR‐375 (*R* = −.61, *p* < .001), log‐hsa‐miR‐216a‐5p (*R* = −.34, *p* = .048), log‐hsa‐miR‐221‐3p (*R* = −.41, *p* = .016) and log‐hsa‐let‐7e‐5p (*R* = −.45, *p* = .008) measured at 6 h after islet infusion were inversely correlated with HbA1c at 6 months after TPIAT (Table 4). Levels of log‐hsa‐miR‐375 (*R* = −.42, *p* = .015), log‐hsa‐miR‐29b‐3p (*R* = −.56, *p* < .001), log‐hsa‐miR‐148a‐3p (*R* = −.35, *p* = .047), log‐hsa‐miR‐221‐3p (*R* = −.65, *p* < .001), log‐hsa‐miR‐320d (*R* = −.55, *p* < .001), log‐hsa‐miR‐125b‐5p (*R* = −.54, *p* < .001), log‐hsa‐let‐7e‐5p (*R* = −.62, *p* < .001) and log‐hsa‐miR‐122‐5p (*R* = −.34, *p* = .055) measured at 6 h after islet infusion were inversely correlated with fasting glucose at 6 months after TPIAT (Table 4). The FDR‐adjusted *p*‐values and bootstrapped FDR‐adjusted *p*‐values for the significant observations are provided in Table 4.

**TABLE 4:** Association of circulating miRNAs at 6 h after islet infusion with metabolic measures before and after TPIAT.

Circulating miRNAs 6 h after islet infusion	Metabolic measures pre‐TPIAT					Metabolic measures 3 months post‐TPIAT					Metabolic measures 6 months post‐TPIAT				
	Parameter	*R*	*p*	*p_adj_*	*BS p_adj_*	Parameter	*R*	*p*	*p_adj_*	*BS p_adj_*	Parameter	R	*p*	*p_adj_*	*BS p_adj_*
*log‐hsa‐miR‐375*	Fasting C‐peptide	.41	.009	.108	.087	Haemoglobin A1c	−.39	.016	.204	.201	Haemoglobin A1c	−.61	<.001	<.001	.002
						Fasting glucose	−.33	.042	.084	.096	Fasting glucose	−.42	.010	.020	.018
*log‐hsa‐miR‐29b‐3p*	*ND*					*ND*					Fasting glucose	−.56	.002	.006	.004
*log‐hsa‐miR‐148a‐3p*	*ND*					*ND*					Fasting glucose	−.35	.047		
*log‐hsa‐miR‐216a‐5p*	Fasting C‐peptide	.32	.041	.252	.232	Fasting C‐peptide	.35	.038	.366	.348	Haemoglobin A1c	−.34	.048	.124	.126
*log‐hsa‐miR‐200c‐3p*	Glucose (2 h PP)	‐.48	.002			Fasting glucose	−.43	.007	.066	.082	*ND*				
*log‐hsa‐miR‐221‐3p*	*ND*					Fasting glucose	−.37	.024	.077	.082	Haemoglobin A1c	−.41	.016	.064	.056
	*ND*					*ND*					Fasting glucose	−.65	<.001	<.001	<.001
*log‐hsa‐miR‐99b‐5p*	*ND*					Fasting glucose	−0.35	0.028	*0.077*	*0.083*	Fasting glucose	−0.43	0.006	0.014	0.015
*log‐hsa‐miR‐7‐5p*	Glucose (2 h PP)	‐0.36	0.027			Fasting glucose	−.40	.011	.066	.082	*ND*				
*log‐hsa‐miR‐320d*	*ND*					*ND*					Fasting glucose	−.55	.002	.006	.004
*log‐hsa‐miR‐125b‐5p*	*ND*					*ND*					Fasting glucose	−.54	.012	.021	.018
*log‐hsa‐let‐7e‐5p*	*ND*					Fasting glucose	−.34	.032	.077	.085	Haemoglobin A1c	−.45	.008	.048	.049
	*ND*					*ND*					Fasting glucose	−.62	<.001	<.001	.001
*log‐hsa‐miR‐122‐5p*	*ND*					*ND*					Fasting glucose	−.34	.055		

PP, postprandial; ND, association not detected; *R*, Pearson's correlation coefficient; TPIAT, total pancreatectomy with islet autotransplantation; *p*_adj_, FDR adjusted *p*‐value; BS *p*_adj_, Bootstrapped FDR adjusted *p*‐value.

**FIGURE 9:**
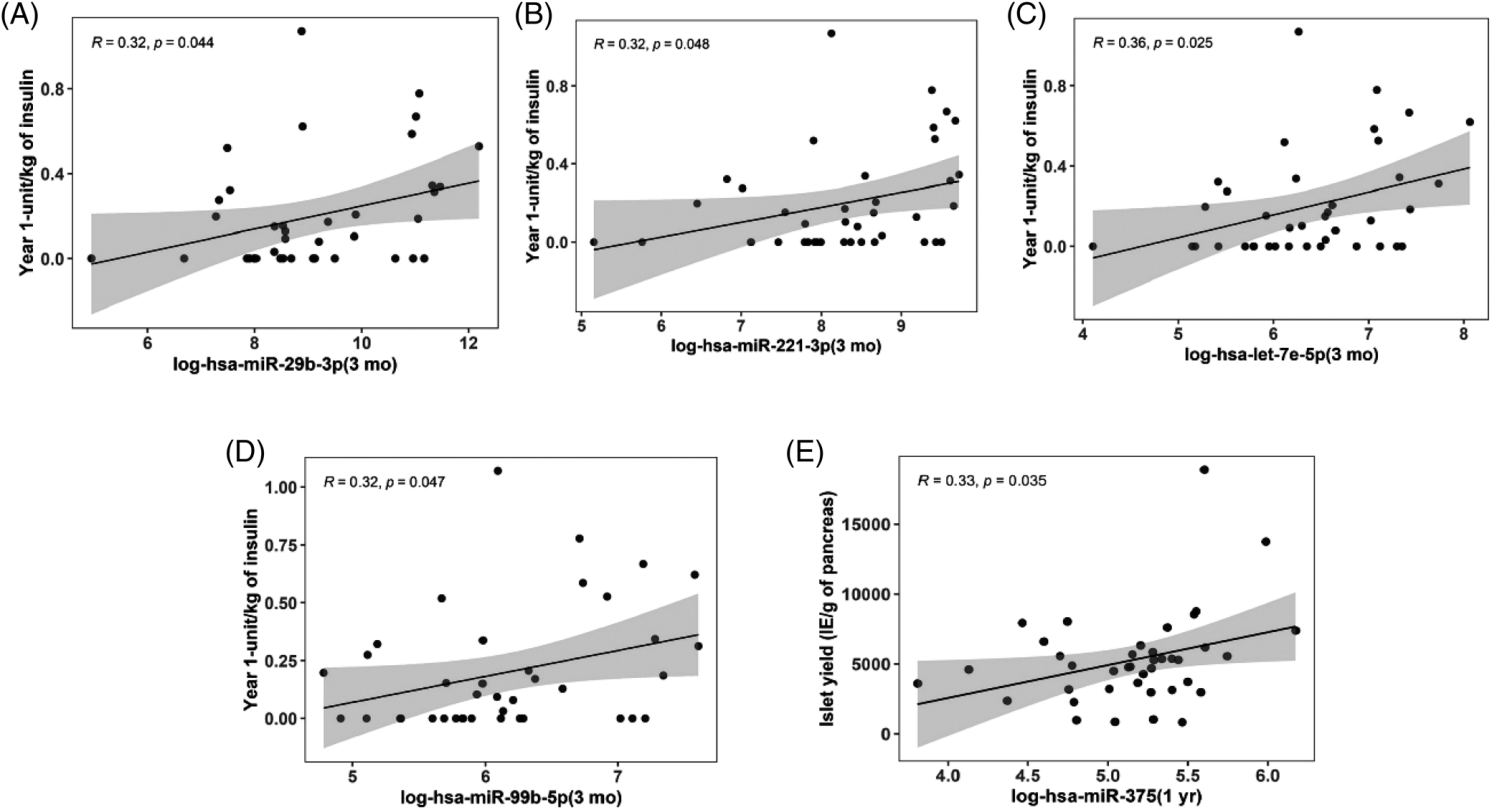
Association of circulating miRNAs at 3 months with metabolic measures after TPIAT. Scatterplots show the correlations of circulating miRNAs measured at 3 months with metabolic measures after TPIAT for (A) log‐*hsa‐miR‐29b‐3p* (3 months) vs. year 1 unit/kg of insulin; (B) log‐*hsa‐miR‐221‐3p* (3 months) vs. year 1 unit/kg of insulin; (C) log‐*hsa‐let‐7e‐5p* (3 months) vs. year 1 unit/kg of insulin; (D) log‐*hsa‐miR‐99b‐5p* (3 months) vs. year 1 unit/kg of insulin; (E) log‐*hsa‐miR‐7‐5p* (1 year) vs. islet yield (IE/g of pancreas). Pearson's correlation coefficient, *R* and *p* values (n = 40) are provided in the scatterplots.

Associations of circulating miRNAs at 3 months and 1 year post‐TPIAT with clinical parameters: Levels of log‐hsa‐miR‐29b‐3p (*R* = .32, *p* = .044, Figure 9A), log‐hsa‐miR‐221‐3p (*R* = .32, *p* = .048, Figure 9B), log‐hsa‐let‐7e‐5p (*R* = .36, *p* = .025, Figure 9C) and log‐hsa‐miR‐99b‐5p (*R* = .32, *p* = .047, Figure 9D) were associated with insulin usage (U/kg body weight) at 1 year post‐TPIAT. The level of log‐hsa‐miR‐375 measured at 1 year post‐TPIAT was associated with islet yield (IE/g of the pancreas) (*R* = .33, *p* = .035, Figure 9E).

## 4 DISCUSSION

One of the factors in achieving post‐transplantation insulin independence is optimal islet yield.^7^ Due to the unpredictable clinical course of the disease and the lack of predictive tools, disease stage and islet isolation outcomes cannot be predicted before TPIAT. Although currently available tests can provide information on metabolic measures, including C‐peptide and glucose levels, islet stress and dysfunction cannot be monitored before an irreversible loss of function before and after TPIAT. In this study, we identified distinctive circulating miRNA signatures of pancreatic damage, predicted miRNA–mRNA networks to identify potential links to CP pathogenesis and identified associations of circulating miRNAs with islet isolation and post‐transplantation functional outcomes (Graphical Abstract).

Our observations of elevated levels of *hsa‐miR‐148a‐3p, hsa‐miR‐221‐3p* and *hsa‐miR‐122‐5p* in CP patients are consistent with other studies.^12^, ^14^, ^15^ Since *hsa‐miR‐375* is an islet damage marker,^8^ reduced levels in circulation may indicate intact islet function and survival before TPIAT in these patients. Elevated levels of *hsa‐miR‐375* at 6 h after islet infusion may indicate islet stress and damage immediately after islet isolation and transplantation. The patients excluded from analyses did not have inflamed pancreas, with islet yields ranging between 161 577 IEQs (4.0 mL tissue volume) and 198 947 IEQs (6.0 mL tissue volume). Although three healthy control samples were also excluded from the analysis, the extent of pancreatic inflammation, fibrosis and damage might influence the quality of circulating miRNAs. For further investigations, we selected *hsa‐miR‐99b‐5p, hsa‐miR‐148a‐3p, hsa‐miR‐221‐3p, hsa‐let‐7e‐5p, hsa‐miR‐122‐5p* and *hsa‐miR‐375* based on base mean, FC and *p* values. Our previous sequencing analysis also identified these selected miRNAs as being differentially released from isolated human islets in exosomes under proinflammatory and hypoxic conditions and in TPIAT patients after islet infusion.^10^ Although our sequencing analysis is limited by a small sample size, consistency with our previous observations suggests that these miRNA signatures may be significant in CP and islet function. We included the following miRNAs based on literature and our previous sequencing data on isolated human islets: *hsa‐miR‐7‐5p*, *hsa‐miR‐216a‐5p*, *hsa‐miR‐200c‐3p, hsa‐miR‐29b‐3p*, *hsa‐miR‐320d* and *hsa‐miR‐125b‐5p*.^13^, ^14^, ^15^, ^29^ We included these additional miRNAs in our follow‐up analysis because we previously identified these miRNAs in islet cultures as differentially altered in proinflammatory conditions ^10^ or CP . ^15^ Our current time‐course analysis allows us to study their profiles following TPIAT and assess whether they were associated with metabolic outcomes. As observations from independent studies may be inconsistent due to differences in disease duration, the status of pancreas inflammation and fibrosis, specimen collection and storage, and techniques for measuring circulating miRNAs, we included these additional miRNAs in our studies to better understand the profiles of circulating miRNAs before and after TPIAT.

The candidate miRNAs must be disease‐ and pancreas‐specific to be non‐invasive circulating miRNA predictors of pancreatic inflammation, islet yield and post‐TPIAT insulin independence. Thus, we assessed whether these selected miRNAs were pancreas‐specific and/or CP‐specific using overrepresentation analysis. Association with Ago2 or exosomes and microvesicles stabilizes the miRNAs in circulation.^30^ Although the selected miRNAs were significantly enriched in the pancreas (except for *hsa‐let‐7e‐5p*), our current data revealed that other tissues might contribute to the circulating miRNA pool. These miRNAs were also differentially altered in circulation in other diseases. Thus, these selected miRNA candidates are neither pancreas‐specific nor CP‐specific. However, they are enriched in the pancreas and elevated/reduced in circulation in CP patients, which may indicate their involvement in disease‐specific processes, warranting further investigations.

Pathway analysis revealed an overrepresentation of these miRNAs in the pathways indicated in Figure 2C. In the context of CP, activation of Notch signalling plays a vital role in pancreas regeneration and repair.^31^ Gap junction proteins regulate pancreatic stellate cell proliferation, function and activation.^17^ Trans‐endothelial migration of leukocytes is critical in initiating and perpetuating pancreatic inflammation.^32^ A previous report on the miRNA: mRNA regulation network in CP was based on their tissue expression profile, collected from the Gene Expression Atlas. In this independent study, analysis of miRNA–mRNA pathway regulating networks identified the involvement of NOTCH, TGF‐β, wingless‐related integrated site, extracellular matrix and ubiquitin‐mediated proteolysis pathways.^18^ These common findings emphasize the involvement of miRNAs in CP pathogenesis. Further investigations are necessary to identify interactions of miRNAs with mRNAs that regulate leukocyte migration, gap junction proteins, TGF‐β and notch signalling pathways and their relevance to miRNAs in circulation to understand their contributions to disease progression.

Our analysis predicted 152 interactions of miRNAs with 72 small molecules including glucose, morphine, vitamin D3, activin A and metformin. Small molecules can either repress or promote miRNA transcription.^33^ In healthy individuals treated with hydromorphone and oxycodone, *hsa‐miR‐221‐3p* and *let‐7* family were upregulated in circulation.^34^ Given that the *let‐7* family of miRNAs plays integral roles in opioid tolerance,^35^ elevated levels of *hsa‐let‐7e‐5p* and others in circulation in our cohort of CP patients may be associated with opioid treatment and be unrelated to disease characteristics. Our analysis also predicted interactions of *hsa‐miR‐148a‐3p*, *hsa‐miR‐200c‐3p, hsa‐miR‐29b‐3p, hsa‐miR‐375* and *hsa‐miR‐122‐5p* with glucose. Previously, we observed that isolated human islets released these miRNAs into culture media in proinflammatory and hypoxic conditions.^10^ Elevated levels of *hsa‐miR‐148a‐3p* were also observed in autoantibody‐positive, non‐diabetic, recent‐onset type 1 diabetic and longstanding type 1 diabetic individuals.^36^ Our analysis also predicted 22 mRNA targets of the selected miRNAs with a risk for pancreatitis (Figure 4). Considering all our observations, the selected miRNAs may be involved in specific disease processes in CP. Their circulating levels may indicate subtle alterations in islet function in CP patients even though preoperative metabolic measures indicated optimal islet function and reflect specific unexplored disease states. Future investigations should focus on experimental validation of these predicted interactions of miRNAs with small molecules, genes associated with risk for pancreatitis and pathways linked to CP pathogenesis. These experiments will help us understand why these miRNAs are elevated in circulation in CP patients and establish their contributions to CP pathogenesis.

Receiver operating characteristic analysis revealed that *hsa‐miR‐125b‐5p* and *hsa‐miR‐122‐5p* distinguished CP patients from healthy controls with good diagnostic accuracy. Although a subset of other miRNAs could significantly discriminate CP patients from healthy controls, AUC scores were sub‐optimal, ranging between .65 and .7. As *hsa‐miR‐122‐5p* is a liver‐abundant miRNA, it is unclear as to why it is elevated in CP patients and discriminated CP patients from healthy controls. Whether perpetual pancreatic inflammation and fibrosis affect hepatocyte function and activate common progenitor cells is unknown. Another possibility is that chronic narcotics usage may induce liver damage in these patients without clinical manifestation. Further investigations are necessary to understand whether our findings are specific to CP by comparing circulating miRNA profiles in patients with CP to those with hepatitis or chronic narcotics usage.

In our previous study, POST (Prospective Observational Cohort Study of TPIAT), involving 139 participants from 9 institutions,^6^ we did not follow up on profiles of the circulating miRNAs after TPIAT. In our current study, we did not validate our sequencing observations in an independent cohort of CP patients. We conducted further studies using patient samples (n = 40, including patients from our sequencing analysis) collected before and after TPIAT. One of the objectives of our current study was to investigate whether circulating levels of selected miRNAs were altered following TPIAT. As expected, islet‐, acinar cell‐ and liver‐specific miRNAs were increased at 6 h after islet infusion, indicating islet and acinar cell stress and liver inflammation or damage immediately in the post‐operative period. These miRNAs returned to baseline levels 3 months following TPIAT, reflecting recovery. Time‐course analysis during and after islet infusion revealed that circulating islet stress and damage miRNA levels peak at the end of islet infusion and normalize to baseline levels 7 days after transplantation.^10^ As these miRNAs measured at 1‐year post‐TPIAT were not associated with functional outcomes, it is unclear whether they reflect subtle changes in islet graft function and survival.

When adjusted for multiple comparisons, circulating miRNAs were not associated with the duration of disease symptoms, consistent with previous observations in the POST study cohort. While circulating miRNAs did not correlate with age and body mass index in our current study, circulating *hsa‐miR‐200c‐3p, hsa‐miR‐148a‐3p* and *hsa‐miR‐221‐3p* were significantly higher in adults compared to paediatric patients in the POST study.^6^ We did not compare profiles of circulating miRNAs between patients of different aetiologies and with or without exocrine insufficiency because sample size and statistical power would be reduced by sub‐group analyses. Nevertheless, levels of circulating miRNAs could be influenced by pancreatic tissue volume and severity of the disease, warranting further investigations.^6^

As we did not measure pancreatic tissue levels of these miRNAs, it is unclear whether the elevated levels in circulation are due to increased tissue miRNA levels in CP. Interestingly, *hsa‐miR‐375* measured 1 year after transplantation was associated with islet yield, indicating islet survival 1 year after transplantation. Of the 63 significant associations, 33 remained significant after correction for multiple comparisons (23 associations, *BS p_adj_* < .05). The strong inverse associations of selected miRNA candidates measured before TPIAT and 6 h after infusion with post‐TPIAT metabolic outcomes highlight their potential as predictors of islet survival and function after transplantation. Although the patients were on insulin therapy to maintain HbA1c levels under 6.5%, C‐peptide was detectable and in the normal range in the fasted state at 3 months and up to 1 year after transplantation. Plasma glucagon was also detectable (at sub‐physiological levels) 1 year after transplantation. As exogenous insulin therapy may influence circulating miRNA levels apart from metabolic measures, future studies should test our hypothesis by comparing the associations of circulating miRNAs between patients with and without insulin independence 1 year after transplantation.

Weak correlations observed in our study could be due to the cohort heterogeneity and the small sample size. Our small RNA sequencing analysis is limited by an imbalanced sample size between controls (n = 3) and patients (n = 15). Thus, we may have missed important circulating miRNA signatures of pancreatic inflammation and damage. Further, to aid comparisons of independent data in the future, we quantified absolute levels of miRNAs in circulation using miRNA mimics. Limitations of our study include patient retention, follow‐up data availability and potential differences in patient sample collection and handling. Overall, our current study highlights miRNA–mRNA networks with possible links to the pathogenesis of CP and identifies circulating miRNA signatures with potential as biomarkers of pancreatic damage. Experimental validation of the highlighted miRNA–mRNA networks in the pancreas in mouse models of CP will shed light on miRNA contributions to CP pathogenesis and help identify novel therapeutic targets. Further large‐scale investigations are necessary to establish circulating miRNA signatures as predictors of pancreatic inflammation and fibrosis, islet yield, post‐TPIAT insulin independence and clinical outcomes.

## CONFLICT OF INTEREST STATEMENT

All authors declare that there is no conflict of interest.

## ETHICAL APPROVAL

Ethical approval for this study was obtained from the Institutional Review Board (IRB #010‐150). Written informed consent was obtained from participants for their study participation and the anonymised data publication. All procedures were in accordance with IRB approved protocols.

## Supporting information

Supporting Information

## Data Availability

All data are archived in the institutional network drive and are available upon request. The sequencing data have been deposited in NCBI's Gene Expression Omnibus^37^, ^38^ and are accessible through GEO Series accession number GSE229528. (https://www.ncbi.nlm.nih.gov/geo/query/acc.cgi?acc=GSE229528).
