# Supplementary material for: Clinical and biological significance of circulating miRNAs in chronic pancreatitis patients undergoing total pancreatectomy with islet autotransplantation
Source: Clin Transl Med. 2023 Oct 17;13(10):e1434. doi: 10.1002/ctm2.1434 (PMC10579997; doi:10.1002/ctm2.1434)
Supplement: Supplementary file 1 — Supporting Information [file CTM2-13-e1434-s001.docx]

***Supplementary Material:***

**Clinical and biological significance of circulating miRNAs in chronic pancreatitis patients undergoing total pancreatectomy with islet autotransplantation**

Srividya Vasu^1^, Giovanna Saracino^2^, Carly M. Darden^1^, Kenjiro Kumano^3^, Yang Liu^2^, Michael C. Lawrence^1^, Bashoo Naziruddin^2^

**Supplementary Methods**

**Study design:**

For analysis of differentially expressed miRNAs in circulation in CP patients, plasma samples were collected from CP patients (n=18, age >18 and <55, sex, race, etiology, and ethnicity) before TPIAT at Baylor University Medical Center (BUMC), Dallas. Healthy donor plasma samples (n=6, age >18 and <55, sex, race, ethnicity, no medications, body mass index, BMI < 26) were obtained from BUMC Biobank immediately after blood draw, processed, aliquoted, and stored at -80 C until further analyses. To study associations of selected circulating miRNA candidates with clinical measures, we included patients (n = 40) of any age, sex, race, and ethnicity that had stored plasma samples collected before TPIAT and at 6 h after islet infusion, 90, and 365 days after TPIAT at Baylor University Medical Center, Dallas. Patients diagnosed with other pancreatic diseases, including cancer, were excluded. The following patient data were collected: demographics (age, gender, race, ethnicity), anthropometric measures (weight, height, body mass index), medical comorbidities (history, gastroparesis or bowel dysmotility diagnosis), prior surgery (pancreas and non-pancreas specific), psychological comorbidities (depression, anxiety), health-related quality of life, visual analog pain scale, etiology (Toxic/metabolic, Idiopathic, Genetic, Autoimmune, Recurrent and severe acute pancreatitis, Obstructive – TIGAR-O), diagnostic tests for pancreatitis, disease duration, pain management, hospitalization history, and exocrine sufficiency (stool elastase test, pancreatic enzyme therapy—yes/no). The following details of TPIAT were collected: date, islet processing location, islet yield/number, islet infusion site(s), islet culture for microbial contamination testing, surgery details, surgery complications (infection, bleeding, blood transfusions, biliary duct obstruction or leak, portal vein thrombosis, severe hypoglycemia), length of hospitalization, and any readmission. After TPIAT, data on functional outcomes were collected at 30, 90, 180, and 365 days. These include insulin independence (no insulin past 14 days and HbA1c ≤ 6.5), insulin dose (units/kg body weight), fasting C-peptide, fasting glucose, HbA1c, and any hypoglycemic episode. Data on pain resolution, health-related quality of life, weight, body mass index, pancreatic enzyme therapy, fat-soluble vitamin deficiencies, comorbidities, hospitalization, and any other medical history were collected. Plasma was collected in EDTA tubes, processed, aliquoted, and stored frozen at – 80°C until analyses. The institutional review board reviewed and approved the study protocol (IRB #010-150). Informed consent was obtained from each study participant.

**Supplementary Table 1. Tissues with significant expression of selected miRNAs**

| **Organ** | ***P*  value** | **Adjusted *P* value** | **miRNAs** |
| --- | --- | --- | --- |
| Mammary gland | 0.0000004 | <0.001 | hsa-mir-29b-3p, hsa-mir-148a-3p, hsa-mir-7-5p, hsa-mir-221-3p, hsa-mir-122-5p, hsa-mir-125b-5p, hsa-mir-200c-3p, hsa-mir-99b-5p, hsa-mir-375, hsa-mir-320d |
| Pancreas | 0.00003 | <0.001 | hsa-mir-29b-3p, hsa-mir-148a-3p, hsa-mir-7-5p, hsa-mir-216a-5p, hsa-mir-221-3p, hsa-mir-122-5p, hsa-mir-125b-5p, hsa-mir-200c-3p, hsa-mir-99b-5p, hsa-mir-375, hsa-mir-320d |
| Lung | 0.00005 | <0.001 | hsa-mir-29b-3p, hsa-mir-7-5p, hsa-mir-221-3p, hsa-mir-200c-3p, hsa-mir-375 |
| Head/neck | 0.0002 | 0.001 | hsa-mir-7-5p, hsa-mir-200c-3p, hsa-mir-99b-5p |
| Cervix | 0.0002 | 0.001 | hsa-mir-29b-3p, hsa-mir-148a-3p, hsa-mir-7-5p, hsa-mir-216a-5p, hsa-mir-221-3p, hsa-mir-125b-5p, hsa-mir-200c-3p, hsa-mir-99b-5p, hsa-mir-375 |
| Breast cancer | 0.0006 | 0.002 | hsa-mir-29b-3p, hsa-mir-7-5p, hsa-mir-221-3p, hsa-mir-125b-5p |
| Liver | 0.0008 | 0.003 | hsa-mir-29b-3p, hsa-mir-216a-5p, hsa-mir-221-3p, hsa-mir-122-5p, hsa-mir-200c-3p |
| Gastric | 0.001 | 0.004 | hsa-mir-29b-3p, hsa-mir-200c-3p, hsa-mir-375 |
| Embryo | 0.001 | 0.004 | hsa-mir-29b-3p, hsa-mir-148a-3p, hsa-mir-221-3p, hsa-mir-200c-3p |
| Eye | 0.002 | 0.005 | hsa-mir-29b-3p, hsa-mir-200c-3p |
| Uterus/endometrium | 0.003 | 0.006 | hsa-mir-221-3p, hsa-mir-200c-3p |
| Kidney | 0.003 | 0.006 | hsa-mir-29b-3p, hsa-mir-148a-3p, hsa-mir-7-5p, hsa-mir-216a-5p, hsa-mir-221-3p, hsa-mir-125b-5p, hsa-mir-200c-3p, hsa-mir-99b-5p, hsa-mir-375, hsa-mir-320d |
| Skin | 0.003 | 0.006 | hsa-mir-221-3p, hsa-mir-125b-5p, hsa-mir-99b-5p |
| Umbilical vein | 0.006 | 0.012 | hsa-mir-7-5p, hsa-mir-221-3p, hsa-mir-200c-3p |
| Brain | 0.012 | 0.02 | hsa-mir-7-5p, hsa-mir-221-3p, hsa-mir-125b-5p, hsa-mir-99b-5p |
| Bone | 0.013 | 0.02 | hsa-mir-148a-3p, hsa-mir-200c-3p, hsa-mir-375 |
| Stomach | 0.015 | 0.02 | hsa-mir-29b-3p, hsa-mir-375 |
| Cartilage | 0.018 | 0.02 | hsa-mir-125b-5p |
| Mouth | 0.018 | 0.02 | hsa-mir-375 |
| Bone marrow | 0.034 | 0.04 | hsa-mir-29b-3p, hsa-mir-148a-3p, hsa-mir-7-5p, hsa-mir-216a-5p, hsa-mir-221-3p, hsa-mir-125b-5p, hsa-mir-200c-3p, hsa-mir-99b-5p, hsa-mir-320d |

*Significance determined by adjusted *p* value < 0.05, hypergeometric test. miRNet function explorer tool queried in miRNA tissue database.

**Supplementary Table 2. miEAA-miRNA Enrichment and Annotation: Analysis results for diseases (MNDR) in the category of overrepresentation**

| **Subcategory** | **P  value** | **P adjusted** | **Q value** | **Expected** | **Observed** | **miRNAs/precursors** |
| --- | --- | --- | --- | --- | --- | --- |
| Male Infertility | 1.69e-11 | 4.45e-9 | 4.45e-9 | 0.268966 | 8 | hsa-miR-375-3p; hsa-miR-29b-3p; hsa-miR-148a-3p; hsa-miR-7-5p; hsa-miR-221-3p; hsa-miR-122-5p; hsa-miR-200c-3p; hsa-miR-125b-5p |
| Fatty Liver | 1.99e-11 | 4.45e-9 | 4.45e-9 | 0.274138 | 8 | hsa-miR-375-3p; hsa-miR-29b-3p; hsa-miR-148a-3p; hsa-miR-216a-5p; hsa-miR-7-5p; hsa-miR-221-3p; hsa-miR-200c-3p; hsa-miR-125b-5p |
| Psoriasis | 2.41e-11 | 4.45e-9 | 4.45e-9 | 0.460345 | 9 | hsa-miR-375-3p; hsa-miR-29b-3p; hsa-miR-148a-3p; hsa-miR-7-5p; hsa-miR-221-3p; hsa-miR-122-5p; hsa-miR-200c-3p; hsa-miR-125b-5p; hsa-miR-99b-5p |
| Viral Hepatitis | 2.72e-11 | 4.45e-9 | 4.45e-9 | 0.284483 | 8 | hsa-miR-375-3p; hsa-miR-29b-3p; hsa-miR-148a-3p; hsa-miR-7-5p; hsa-miR-221-3p; hsa-miR-122-5p; hsa-miR-200c-3p; hsa-miR-125b-5p |
| Type 1 Diabetes Mellitus | 4.27e-11 | 5.58e-9 | 5.58e-9 | 0.3 | 8 | hsa-miR-375-3p; hsa-miR-29b-3p; hsa-miR-148a-3p; hsa-miR-7-5p; hsa-miR-221-3p; hsa-miR-122-5p; hsa-miR-200c-3p; hsa-miR-125b-5p |
| Ewing Sarcoma | 1.26e-10 | 1.38e-8 | 1.38e-8 | 0.341379 | 8 | hsa-miR-375-3p; hsa-miR-29b-3p; hsa-miR-148a-3p; hsa-miR-7-5p; hsa-miR-221-3p; hsa-miR-122-5p; hsa-miR-200c-3p; hsa-miR-125b-5p |
| Facioscapulohumeral Muscular Dystrophy | 2.32e-10 | 2.17e-8 | 2.17e-8 | 0.367241 | 8 | hsa-miR-29b-3p; hsa-miR-148a-3p; hsa-miR-7-5p; hsa-miR-221-3p; hsa-miR-122-5p; hsa-miR-200c-3p; hsa-miR-125b-5p; hsa-miR-99b-5p |
| Precursor Cell Lymphoblastic Leukemia-Lymphoma | 2.86e-10 | 2.34e-8 | 2.34e-8 | 0.910345 | 10 | hsa-miR-375-3p; hsa-miR-29b-3p; hsa-miR-148a-3p; hsa-miR-216a-5p; hsa-miR-7-5p; hsa-miR-221-3p; hsa-miR-122-5p; hsa-miR-200c-3p; hsa-miR-125b-5p; hsa-miR-99b-5p |
| Cocaine-Related Disorders | 6.49e-10 | 2.74e-8 | 2.74e-8 | 0.243103 | 7 | hsa-miR-29b-3p; hsa-miR-148a-3p; hsa-miR-7-5p; hsa-miR-221-3p; hsa-miR-122-5p; hsa-miR-200c-3p; hsa-miR-125b-5p |
| Syndrome | 7.64e-10 | 2.74e-8 | 2.74e-8 | 0.424138 | 8 | hsa-miR-29b-3p; hsa-miR-148a-3p; hsa-miR-216a-5p; hsa-miR-7-5p; hsa-miR-221-3p; hsa-miR-122-5p; hsa-miR-200c-3p; hsa-miR-125b-5p |
| Cholesteatoma | 8.83e-10 | 2.74e-8 | 2.74e-8 | 0.253448 | 7 | hsa-miR-29b-3p; hsa-miR-148a-3p; hsa-miR-7-5p; hsa-miR-221-3p; hsa-miR-122-5p; hsa-miR-200c-3p; hsa-miR-125b-5p |
| Muscular Disorders, Atrophic | 9.32e-10 | 2.74e-8 | 2.74e-8 | 0.434483 | 8 | hsa-miR-375-3p; hsa-miR-29b-3p; hsa-miR-148a-3p; hsa-miR-7-5p; hsa-miR-221-3p; hsa-miR-200c-3p; hsa-miR-125b-5p; hsa-miR-99b-5p |
| Cerebral Hemorrhage | 1.02e-9 | 2.74e-8 | 2.74e-8 | 0.258621 | 7 | hsa-miR-29b-3p; hsa-miR-148a-3p; hsa-miR-7-5p; hsa-miR-221-3p; hsa-miR-122-5p; hsa-miR-200c-3p; hsa-miR-125b-5p |
| Alopecia | 1.02e-9 | 2.74e-8 | 2.74e-8 | 0.258621 | 7 | hsa-miR-29b-3p; hsa-miR-148a-3p; hsa-miR-7-5p; hsa-miR-221-3p; hsa-miR-122-5p; hsa-miR-200c-3p; hsa-miR-125b-5p |
| Amyloidosis | 1.02e-9 | 2.74e-8 | 2.74e-8 | 0.258621 | 7 | hsa-miR-29b-3p; hsa-miR-148a-3p; hsa-miR-7-5p; hsa-miR-221-3p; hsa-miR-122-5p; hsa-miR-200c-3p; hsa-miR-125b-5p |
| Anxiety Disorder | 1.19e-9 | 2.74e-8 | 2.74e-8 | 0.263793 | 7 | hsa-miR-375-3p; hsa-miR-29b-3p; hsa-miR-148a-3p; hsa-miR-7-5p; hsa-miR-221-3p; hsa-miR-200c-3p; hsa-miR-125b-5p |
| Endomyocardial Fibrosis | 1.19e-9 | 2.74e-8 | 2.74e-8 | 0.263793 | 7 | hsa-miR-29b-3p; hsa-miR-148a-3p; hsa-miR-7-5p; hsa-miR-221-3p; hsa-miR-122-5p; hsa-miR-200c-3p; hsa-miR-125b-5p |
| Panic Disorder | 1.19e-9 | 2.74e-8 | 2.74e-8 | 0.263793 | 7 | hsa-miR-29b-3p; hsa-miR-148a-3p; hsa-miR-7-5p; hsa-miR-221-3p; hsa-miR-122-5p; hsa-miR-200c-3p; hsa-miR-125b-5p |
| Coronary Artery Disease | 1.24e-9 | 2.74e-8 | 2.74e-8 | 0.45 | 8 | hsa-miR-375-3p; hsa-miR-29b-3p; hsa-miR-148a-3p; hsa-miR-7-5p; hsa-miR-221-3p; hsa-miR-122-5p; hsa-miR-200c-3p; hsa-miR-125b-5p |
| Diffuse Large B-Cell Lymphoma | 1.24e-9 | 2.74e-8 | 2.74e-8 | 0.45 | 8 | hsa-miR-375-3p; hsa-miR-29b-3p; hsa-miR-148a-3p; hsa-miR-7-5p; hsa-miR-221-3p; hsa-miR-200c-3p; hsa-let-7e-5p; hsa-miR-125b-5p |
| Aging | 1.37e-9 | 2.74e-8 | 2.74e-8 | 0.268966 | 7 | hsa-miR-29b-3p; hsa-miR-148a-3p; hsa-miR-7-5p; hsa-miR-221-3p; hsa-miR-122-5p; hsa-miR-200c-3p; hsa-miR-125b-5p |
| Sezary's Disease | 1.37e-9 | 2.74e-8 | 2.74e-8 | 0.268966 | 7 | hsa-miR-375-3p; hsa-miR-29b-3p; hsa-miR-148a-3p; hsa-miR-221-3p; hsa-miR-122-5p; hsa-miR-200c-3p; hsa-miR-125b-5p |
| Demyelinating Disease | 1.37e-9 | 2.74e-8 | 2.74e-8 | 0.268966 | 7 | hsa-miR-29b-3p; hsa-miR-148a-3p; hsa-miR-7-5p; hsa-miR-221-3p; hsa-miR-122-5p; hsa-miR-200c-3p; hsa-miR-125b-5p |
| Prion Disease | 1.37e-9 | 2.74e-8 | 2.74e-8 | 0.268966 | 7 | hsa-miR-29b-3p; hsa-miR-148a-3p; hsa-miR-7-5p; hsa-miR-221-3p; hsa-miR-122-5p; hsa-miR-200c-3p; hsa-miR-125b-5p |
| Testicular Cancer | 1.37e-9 | 2.74e-8 | 2.74e-8 | 0.268966 | 7 | hsa-miR-375-3p; hsa-miR-29b-3p; hsa-miR-7-5p; hsa-miR-221-3p; hsa-miR-122-5p; hsa-miR-200c-3p; hsa-miR-125b-5p |
| Mouth Neoplasms | 1.50e-9 | 2.74e-8 | 2.74e-8 | 0.460345 | 8 | hsa-miR-375-3p; hsa-miR-29b-3p; hsa-miR-148a-3p; hsa-miR-7-5p; hsa-miR-221-3p; hsa-miR-122-5p; hsa-miR-200c-3p; hsa-miR-125b-5p |
| Distal Muscular Dystrophy | 1.50e-9 | 2.74e-8 | 2.74e-8 | 0.460345 | 8 | hsa-miR-29b-3p; hsa-miR-148a-3p; hsa-miR-7-5p; hsa-miR-221-3p; hsa-miR-122-5p; hsa-miR-200c-3p; hsa-miR-125b-5p; hsa-miR-99b-5p |
| Abortion, Habitual | 1.57e-9 | 2.74e-8 | 2.74e-8 | 0.274138 | 7 | hsa-miR-29b-3p; hsa-miR-148a-3p; hsa-miR-7-5p; hsa-miR-221-3p; hsa-miR-122-5p; hsa-miR-200c-3p; hsa-miR-125b-5p |
| Esophagus | 1.57e-9 | 2.74e-8 | 2.74e-8 | 0.274138 | 7 | hsa-miR-29b-3p; hsa-miR-148a-3p; hsa-miR-7-5p; hsa-miR-221-3p; hsa-miR-122-5p; hsa-miR-200c-3p; hsa-miR-125b-5p |
| Heart Defects, Congenital | 1.57e-9 | 2.74e-8 | 2.74e-8 | 0.274138 | 7 | hsa-miR-29b-3p; hsa-miR-148a-3p; hsa-miR-7-5p; hsa-miR-221-3p; hsa-miR-122-5p; hsa-miR-200c-3p; hsa-miR-125b-5p |
| Hypertrophy, Left Ventricular | 1.57e-9 | 2.74e-8 | 2.74e-8 | 0.274138 | 7 | hsa-miR-29b-3p; hsa-miR-148a-3p; hsa-miR-7-5p; hsa-miR-221-3p; hsa-miR-122-5p; hsa-miR-200c-3p; hsa-miR-125b-5p |
| Liver Failure | 1.57e-9 | 2.74e-8 | 2.74e-8 | 0.274138 | 7 | hsa-miR-29b-3p; hsa-miR-148a-3p; hsa-miR-7-5p; hsa-miR-221-3p; hsa-miR-122-5p; hsa-miR-200c-3p; hsa-miR-125b-5p |
| Myocardial Reperfusion Injury | 1.57e-9 | 2.74e-8 | 2.74e-8 | 0.274138 | 7 | hsa-miR-29b-3p; hsa-miR-148a-3p; hsa-miR-7-5p; hsa-miR-221-3p; hsa-miR-122-5p; hsa-miR-200c-3p; hsa-miR-125b-5p |
| SARS Virus | 1.57e-9 | 2.74e-8 | 2.74e-8 | 0.274138 | 7 | hsa-miR-29b-3p; hsa-miR-148a-3p; hsa-miR-7-5p; hsa-miR-221-3p; hsa-miR-122-5p; hsa-miR-200c-3p; hsa-miR-125b-5p |
| Cerebral Infarction | 1.57e-9 | 2.74e-8 | 2.74e-8 | 0.274138 | 7 | hsa-miR-29b-3p; hsa-miR-148a-3p; hsa-miR-7-5p; hsa-miR-221-3p; hsa-miR-122-5p; hsa-miR-200c-3p; hsa-miR-125b-5p |
| Eclampsia | 1.57e-9 | 2.74e-8 | 2.74e-8 | 0.274138 | 7 | hsa-miR-29b-3p; hsa-miR-148a-3p; hsa-miR-7-5p; hsa-miR-221-3p; hsa-miR-122-5p; hsa-miR-200c-3p; hsa-miR-125b-5p |
| Periodontal Disease | 1.57e-9 | 2.74e-8 | 2.74e-8 | 0.274138 | 7 | hsa-miR-29b-3p; hsa-miR-148a-3p; hsa-miR-7-5p; hsa-miR-221-3p; hsa-miR-122-5p; hsa-miR-200c-3p; hsa-miR-125b-5p |
| Uterine Cervical Neoplasms | 1.66e-9 | 2.74e-8 | 2.74e-8 | 0.72931 | 9 | hsa-miR-375-3p; hsa-miR-29b-3p; hsa-miR-148a-3p; hsa-miR-7-5p; hsa-miR-221-3p; hsa-miR-122-5p; hsa-miR-200c-3p; hsa-miR-125b-5p; hsa-miR-99b-5p |
| Chordoma | 1.77e-9 | 2.74e-8 | 2.74e-8 | 0.734483 | 9 | hsa-miR-29b-3p; hsa-miR-148a-3p; hsa-miR-7-5p; hsa-miR-221-3p; hsa-miR-320d; hsa-miR-122-5p; hsa-let-7e-5p; hsa-miR-125b-5p; hsa-miR-99b-5p |
| Cardiomyopathy | 1.80e-9 | 2.74e-8 | 2.74e-8 | 0.27931 | 7 | hsa-miR-29b-3p; hsa-miR-148a-3p; hsa-miR-7-5p; hsa-miR-221-3p; hsa-miR-122-5p; hsa-miR-200c-3p; hsa-miR-125b-5p |
| Choriocarcinoma | 1.80e-9 | 2.74e-8 | 2.74e-8 | 0.27931 | 7 | hsa-miR-29b-3p; hsa-miR-148a-3p; hsa-miR-7-5p; hsa-miR-221-3p; hsa-miR-122-5p; hsa-miR-200c-3p; hsa-miR-125b-5p |
| Cryptosporidiosis | 1.80e-9 | 2.74e-8 | 2.74e-8 | 0.27931 | 7 | hsa-miR-29b-3p; hsa-miR-148a-3p; hsa-miR-7-5p; hsa-miR-221-3p; hsa-miR-122-5p; hsa-miR-200c-3p; hsa-miR-125b-5p |
| Fatty Liver Disease | 1.80e-9 | 2.74e-8 | 2.74e-8 | 0.27931 | 7 | hsa-miR-29b-3p; hsa-miR-148a-3p; hsa-miR-7-5p; hsa-miR-221-3p; hsa-miR-122-5p; hsa-miR-200c-3p; hsa-miR-125b-5p |
| Spinal Cord Injuries | 2.06e-9 | 3.06e-8 | 3.06e-8 | 0.284483 | 7 | hsa-miR-375-3p; hsa-miR-29b-3p; hsa-miR-148a-3p; hsa-miR-221-3p; hsa-miR-122-5p; hsa-miR-200c-3p; hsa-miR-125b-5p |
| Nephrosclerosis | 2.35e-9 | 3.34e-8 | 3.34e-8 | 0.289655 | 7 | hsa-miR-375-3p; hsa-miR-29b-3p; hsa-miR-148a-3p; hsa-miR-7-5p; hsa-miR-221-3p; hsa-miR-200c-3p; hsa-miR-125b-5p |
| Pulmonary Fibrosis | 2.35e-9 | 3.34e-8 | 3.34e-8 | 0.289655 | 7 | hsa-miR-29b-3p; hsa-miR-148a-3p; hsa-miR-7-5p; hsa-miR-221-3p; hsa-miR-122-5p; hsa-miR-200c-3p; hsa-miR-125b-5p |
| Osteoporosis | 2.68e-9 | 3.72e-8 | 3.72e-8 | 0.294828 | 7 | hsa-miR-29b-3p; hsa-miR-148a-3p; hsa-miR-216a-5p; hsa-miR-7-5p; hsa-miR-221-3p; hsa-miR-122-5p; hsa-miR-125b-5p |
| Hepatitis B | 2.78e-9 | 3.79e-8 | 3.79e-8 | 0.496552 | 8 | hsa-miR-375-3p; hsa-miR-29b-3p; hsa-miR-148a-3p; hsa-miR-7-5p; hsa-miR-221-3p; hsa-miR-122-5p; hsa-miR-200c-3p; hsa-miR-125b-5p |
| Thyroid Cancer | 2.96e-9 | 3.89e-8 | 3.89e-8 | 1.14828 | 10 | hsa-miR-375-3p; hsa-miR-29b-3p; hsa-miR-148a-3p; hsa-miR-7-5p; hsa-miR-221-3p; hsa-miR-122-5p; hsa-miR-200c-3p; hsa-let-7e-5p; hsa-miR-125b-5p; hsa-miR-99b-5p |
| Ehrlich Tumor Carcinoma | 3.04e-9 | 3.89e-8 | 3.89e-8 | 0.3 | 7 | hsa-miR-375-3p; hsa-miR-29b-3p; hsa-miR-148a-3p; hsa-miR-7-5p; hsa-miR-221-3p; hsa-miR-200c-3p; hsa-miR-125b-5p |
| Pheochromocytoma | 3.04e-9 | 3.89e-8 | 3.89e-8 | 0.3 | 7 | hsa-miR-29b-3p; hsa-miR-148a-3p; hsa-miR-7-5p; hsa-miR-221-3p; hsa-miR-122-5p; hsa-miR-200c-3p; hsa-miR-125b-5p |
| Retinoblastoma | 3.24e-9 | 4.02e-8 | 4.02e-8 | 1.15862 | 10 | hsa-miR-375-3p; hsa-miR-29b-3p; hsa-miR-148a-3p; hsa-miR-7-5p; hsa-miR-221-3p; hsa-miR-320d; hsa-miR-122-5p; hsa-miR-200c-3p; hsa-let-7e-5p; hsa-miR-125b-5p |
| Endometriosis | 3.29e-9 | 4.02e-8 | 4.02e-8 | 0.506897 | 8 | hsa-miR-375-3p; hsa-miR-29b-3p; hsa-miR-148a-3p; hsa-miR-7-5p; hsa-miR-221-3p; hsa-miR-200c-3p; hsa-miR-125b-5p; hsa-miR-99b-5p |
| Barrett’s Esophagus | 3.44e-9 | 4.02e-8 | 4.02e-8 | 0.305172 | 7 | hsa-miR-375-3p; hsa-miR-29b-3p; hsa-miR-148a-3p; hsa-miR-7-5p; hsa-miR-221-3p; hsa-miR-200c-3p; hsa-miR-125b-5p |
| Aortic Valve Stenosis | 3.44e-9 | 4.02e-8 | 4.02e-8 | 0.305172 | 7 | hsa-miR-29b-3p; hsa-miR-148a-3p; hsa-miR-7-5p; hsa-miR-221-3p; hsa-miR-122-5p; hsa-miR-200c-3p; hsa-miR-125b-5p |
| Tongue Cancer | 3.44e-9 | 4.02e-8 | 4.02e-8 | 0.305172 | 7 | hsa-miR-29b-3p; hsa-miR-148a-3p; hsa-miR-7-5p; hsa-miR-221-3p; hsa-miR-122-5p; hsa-miR-200c-3p; hsa-miR-125b-5p |
| Stroke | 3.89e-9 | 4.38e-8 | 4.38e-8 | 0.310345 | 7 | hsa-miR-29b-3p; hsa-miR-148a-3p; hsa-miR-221-3p; hsa-miR-122-5p; hsa-miR-200c-3p; hsa-let-7e-5p; hsa-miR-125b-5p |
| Skin Cancer | 3.89e-9 | 4.38e-8 | 4.38e-8 | 0.310345 | 7 | hsa-miR-29b-3p; hsa-miR-148a-3p; hsa-miR-7-5p; hsa-miR-221-3p; hsa-miR-122-5p; hsa-miR-200c-3p; hsa-miR-125b-5p |
| Alzheimer’s Disease | 4.85e-9 | 5.38e-8 | 5.38e-8 | 2.4569 | 12 | hsa-miR-375-3p; hsa-miR-29b-3p; hsa-miR-148a-3p; hsa-miR-216a-5p; hsa-miR-7-5p; hsa-miR-221-3p; hsa-miR-320d; hsa-miR-122-5p; hsa-miR-200c-3p; hsa-let-7e-5p; hsa-miR-125b-5p; hsa-miR-99b-5p |
| Gastrointestinal System Cancer | 5.53e-9 | 5.93e-8 | 5.93e-8 | 0.325862 | 7 | hsa-miR-375-3p; hsa-miR-29b-3p; hsa-miR-148a-3p; hsa-miR-221-3p; hsa-miR-122-5p; hsa-miR-200c-3p; hsa-miR-125b-5p |
| Sarcoma | 5.53e-9 | 5.93e-8 | 5.93e-8 | 0.325862 | 7 | hsa-miR-29b-3p; hsa-miR-148a-3p; hsa-miR-7-5p; hsa-miR-221-3p; hsa-miR-122-5p; hsa-miR-200c-3p; hsa-miR-125b-5p |
| Inflammation | 6.20e-9 | 6.54e-8 | 6.54e-8 | 0.331034 | 7 | hsa-miR-375-3p; hsa-miR-29b-3p; hsa-miR-148a-3p; hsa-miR-7-5p; hsa-miR-221-3p; hsa-miR-200c-3p; hsa-miR-125b-5p |
| Prostatic Neoplasms | 6.69e-9 | 6.95e-8 | 6.95e-8 | 1.77414 | 11 | hsa-miR-375-3p; hsa-miR-29b-3p; hsa-miR-148a-3p; hsa-miR-216a-5p; hsa-miR-7-5p; hsa-miR-221-3p; hsa-miR-122-5p; hsa-miR-200c-3p; hsa-let-7e-5p; hsa-miR-125b-5p; hsa-miR-99b-5p |
| Salivary Gland Cancer | 8.63e-9 | 8.82e-8 | 8.82e-8 | 0.346552 | 7 | hsa-miR-375-3p; hsa-miR-29b-3p; hsa-miR-148a-3p; hsa-miR-221-3p; hsa-miR-200c-3p; hsa-miR-125b-5p; hsa-miR-99b-5p |
| B-Cell Lymphoma | 1.18e-8 | 1.19e-7 | 1.19e-7 | 1.31897 | 10 | hsa-miR-29b-3p; hsa-miR-148a-3p; hsa-miR-7-5p; hsa-miR-221-3p; hsa-miR-320d; hsa-miR-122-5p; hsa-miR-200c-3p; hsa-let-7e-5p; hsa-miR-125b-5p; hsa-miR-99b-5p |
| Renal Cell Carcinoma | 1.23e-8 | 1.22e-7 | 1.22e-7 | 0.910345 | 9 | hsa-miR-375-3p; hsa-miR-29b-3p; hsa-miR-148a-3p; hsa-miR-7-5p; hsa-miR-221-3p; hsa-miR-122-5p; hsa-miR-200c-3p; hsa-miR-125b-5p; hsa-miR-99b-5p |
| Synovial Sarcoma | 1.44e-8 | 1.40e-7 | 1.40e-7 | 0.925862 | 9 | hsa-miR-29b-3p; hsa-miR-148a-3p; hsa-miR-7-5p; hsa-miR-221-3p; hsa-miR-122-5p; hsa-miR-200c-3p; hsa-let-7e-5p; hsa-miR-125b-5p; hsa-miR-99b-5p |
| Adenocarcinoma | 1.59e-8 | 1.52e-7 | 1.52e-7 | 0.615517 | 8 | hsa-miR-375-3p; hsa-miR-29b-3p; hsa-miR-148a-3p; hsa-miR-7-5p; hsa-miR-221-3p; hsa-miR-200c-3p; hsa-miR-125b-5p; hsa-miR-99b-5p |
| HIV Infections | 1.60e-8 | 1.52e-7 | 1.52e-7 | 0.377586 | 7 | hsa-miR-29b-3p; hsa-miR-148a-3p; hsa-miR-7-5p; hsa-miR-221-3p; hsa-miR-122-5p; hsa-miR-200c-3p; hsa-miR-125b-5p |
| Crohns Disease | 1.76e-8 | 1.65e-7 | 1.65e-7 | 0.382759 | 7 | hsa-miR-375-3p; hsa-miR-29b-3p; hsa-miR-148a-3p; hsa-miR-7-5p; hsa-miR-221-3p; hsa-miR-200c-3p; hsa-miR-125b-5p |
| Moyamoya Disease | 2.51e-8 | 2.30e-7 | 2.30e-7 | 0.651724 | 8 | hsa-miR-29b-3p; hsa-miR-148a-3p; hsa-miR-7-5p; hsa-miR-221-3p; hsa-miR-320d; hsa-miR-122-5p; hsa-miR-200c-3p; hsa-miR-125b-5p |
| Type 2 Diabetes Mellitus | 2.57e-8 | 2.30e-7 | 2.30e-7 | 0.403448 | 7 | hsa-miR-375-3p; hsa-miR-29b-3p; hsa-miR-148a-3p; hsa-miR-221-3p; hsa-miR-122-5p; hsa-miR-200c-3p; hsa-miR-125b-5p |
| Ulcerative Colitis | 2.57e-8 | 2.30e-7 | 2.30e-7 | 0.403448 | 7 | hsa-miR-375-3p; hsa-miR-29b-3p; hsa-miR-148a-3p; hsa-miR-7-5p; hsa-miR-221-3p; hsa-miR-200c-3p; hsa-miR-125b-5p |
| Burkitt Lymphoma | 3.88e-8 | 3.14e-7 | 3.14e-7 | 1.03448 | 9 | hsa-miR-375-3p; hsa-miR-29b-3p; hsa-miR-148a-3p; hsa-miR-7-5p; hsa-miR-221-3p; hsa-miR-200c-3p; hsa-let-7e-5p; hsa-miR-125b-5p; hsa-miR-99b-5p |
| Hepatitis | 4.35e-8 | 3.14e-7 | 3.14e-7 | 0.434483 | 7 | hsa-miR-29b-3p; hsa-miR-148a-3p; hsa-miR-7-5p; hsa-miR-221-3p; hsa-miR-122-5p; hsa-miR-200c-3p; hsa-miR-125b-5p |
| Influenza, Human | 5.45e-8 | 3.14e-7 | 3.14e-7 | 0.253448 | 6 | hsa-miR-29b-3p; hsa-miR-148a-3p; hsa-miR-221-3p; hsa-miR-122-5p; hsa-miR-200c-3p; hsa-miR-125b-5p |
| Squamous Cell Carcinoma | 6.00e-8 | 3.14e-7 | 3.14e-7 | 1.08621 | 9 | hsa-miR-375-3p; hsa-miR-29b-3p; hsa-miR-148a-3p; hsa-miR-216a-5p; hsa-miR-7-5p; hsa-miR-221-3p; hsa-miR-122-5p; hsa-miR-200c-3p; hsa-miR-125b-5p |
| Non-Hodgkin Lymphoma | 6.05e-8 | 3.14e-7 | 3.14e-7 | 0.455172 | 7 | hsa-miR-29b-3p; hsa-miR-148a-3p; hsa-miR-7-5p; hsa-miR-221-3p; hsa-miR-122-5p; hsa-miR-200c-3p; hsa-miR-125b-5p |
| Child Development Disorders, Pervasive | 6.18e-8 | 3.14e-7 | 3.14e-7 | 0.258621 | 6 | hsa-miR-29b-3p; hsa-miR-148a-3p; hsa-miR-7-5p; hsa-miR-221-3p; hsa-miR-122-5p; hsa-miR-125b-5p |
| Ischemic Preconditioning | 6.18e-8 | 3.14e-7 | 3.14e-7 | 0.258621 | 6 | hsa-miR-29b-3p; hsa-miR-148a-3p; hsa-miR-7-5p; hsa-miR-221-3p; hsa-miR-122-5p; hsa-miR-125b-5p |
| Acute Kidney Failure | 6.18e-8 | 3.14e-7 | 3.14e-7 | 0.258621 | 6 | hsa-miR-29b-3p; hsa-miR-148a-3p; hsa-miR-7-5p; hsa-miR-221-3p; hsa-miR-122-5p; hsa-miR-125b-5p |
| Brain Ischemia | 6.18e-8 | 3.14e-7 | 3.14e-7 | 0.258621 | 6 | hsa-miR-29b-3p; hsa-miR-148a-3p; hsa-miR-7-5p; hsa-miR-221-3p; hsa-miR-122-5p; hsa-miR-125b-5p |
| Central Nervous System Disease | 6.18e-8 | 3.14e-7 | 3.14e-7 | 0.258621 | 6 | hsa-miR-29b-3p; hsa-miR-148a-3p; hsa-miR-7-5p; hsa-miR-221-3p; hsa-miR-122-5p; hsa-miR-125b-5p |
| Interstitial Cystitis | 6.18e-8 | 3.14e-7 | 3.14e-7 | 0.258621 | 6 | hsa-miR-29b-3p; hsa-miR-148a-3p; hsa-miR-7-5p; hsa-miR-221-3p; hsa-miR-200c-3p; hsa-miR-125b-5p |
| Pulmonary Embolism | 6.18e-8 | 3.14e-7 | 3.14e-7 | 0.258621 | 6 | hsa-miR-29b-3p; hsa-miR-148a-3p; hsa-miR-7-5p; hsa-miR-221-3p; hsa-miR-122-5p; hsa-miR-125b-5p |
| Stomach Disease | 6.18e-8 | 3.14e-7 | 3.14e-7 | 0.258621 | 6 | hsa-miR-29b-3p; hsa-miR-148a-3p; hsa-miR-7-5p; hsa-miR-221-3p; hsa-miR-122-5p; hsa-miR-125b-5p |
| Multiple Myeloma | 6.76e-8 | 3.14e-7 | 3.14e-7 | 1.57241 | 10 | hsa-miR-375-3p; hsa-miR-29b-3p; hsa-miR-148a-3p; hsa-miR-7-5p; hsa-miR-221-3p; hsa-miR-122-5p; hsa-miR-200c-3p; hsa-let-7e-5p; hsa-miR-125b-5p; hsa-miR-99b-5p |
| Acute Lung Injury | 6.99e-8 | 3.14e-7 | 3.14e-7 | 0.263793 | 6 | hsa-miR-29b-3p; hsa-miR-148a-3p; hsa-miR-7-5p; hsa-miR-221-3p; hsa-miR-122-5p; hsa-miR-125b-5p |
| Arrhythmias, Cardiac | 6.99e-8 | 3.14e-7 | 3.14e-7 | 0.263793 | 6 | hsa-miR-29b-3p; hsa-miR-148a-3p; hsa-miR-7-5p; hsa-miR-221-3p; hsa-miR-122-5p; hsa-miR-125b-5p |
| Atrophy | 6.99e-8 | 3.14e-7 | 3.14e-7 | 0.263793 | 6 | hsa-miR-29b-3p; hsa-miR-148a-3p; hsa-miR-7-5p; hsa-miR-221-3p; hsa-miR-122-5p; hsa-miR-125b-5p |
| Autoimmune Diseases | 6.99e-8 | 3.14e-7 | 3.14e-7 | 0.263793 | 6 | hsa-miR-29b-3p; hsa-miR-148a-3p; hsa-miR-7-5p; hsa-miR-221-3p; hsa-miR-122-5p; hsa-miR-125b-5p |
| Brain Ischemia | 6.99e-8 | 3.14e-7 | 3.14e-7 | 0.263793 | 6 | hsa-miR-29b-3p; hsa-miR-148a-3p; hsa-miR-7-5p; hsa-miR-221-3p; hsa-miR-122-5p; hsa-miR-125b-5p |
| Cervical Intraepithelial Neoplasia | 6.99e-8 | 3.14e-7 | 3.14e-7 | 0.263793 | 6 | hsa-miR-29b-3p; hsa-miR-148a-3p; hsa-miR-7-5p; hsa-miR-221-3p; hsa-miR-122-5p; hsa-miR-125b-5p |
| Endothelium, Vascular | 6.99e-8 | 3.14e-7 | 3.14e-7 | 0.263793 | 6 | hsa-miR-29b-3p; hsa-miR-148a-3p; hsa-miR-7-5p; hsa-miR-221-3p; hsa-miR-122-5p; hsa-miR-125b-5p |
| Hiv-1 | 6.99e-8 | 3.14e-7 | 3.14e-7 | 0.263793 | 6 | hsa-miR-29b-3p; hsa-miR-148a-3p; hsa-miR-7-5p; hsa-miR-221-3p; hsa-miR-122-5p; hsa-miR-200c-3p |
| Marek Disease | 6.99e-8 | 3.14e-7 | 3.14e-7 | 0.263793 | 6 | hsa-miR-29b-3p; hsa-miR-148a-3p; hsa-miR-221-3p; hsa-miR-122-5p; hsa-miR-200c-3p; hsa-miR-125b-5p |
| Neoplasms, Glandular And Epithelial | 6.99e-8 | 3.14e-7 | 3.14e-7 | 0.263793 | 6 | hsa-miR-29b-3p; hsa-miR-148a-3p; hsa-miR-7-5p; hsa-miR-221-3p; hsa-miR-122-5p; hsa-miR-125b-5p |
| Antiphospholipid Syndrome | 6.99e-8 | 3.14e-7 | 3.14e-7 | 0.263793 | 6 | hsa-miR-29b-3p; hsa-miR-148a-3p; hsa-miR-7-5p; hsa-miR-221-3p; hsa-miR-122-5p; hsa-miR-125b-5p |
| Aortic Valve Insufficiency | 6.99e-8 | 3.14e-7 | 3.14e-7 | 0.263793 | 6 | hsa-miR-29b-3p; hsa-miR-148a-3p; hsa-miR-7-5p; hsa-miR-221-3p; hsa-miR-122-5p; hsa-miR-125b-5p |
| Cataract | 6.99e-8 | 3.14e-7 | 3.14e-7 | 0.263793 | 6 | hsa-miR-29b-3p; hsa-miR-148a-3p; hsa-miR-7-5p; hsa-miR-221-3p; hsa-miR-122-5p; hsa-miR-125b-5p |
| Dementia | 6.99e-8 | 3.14e-7 | 3.14e-7 | 0.263793 | 6 | hsa-miR-29b-3p; hsa-miR-148a-3p; hsa-miR-7-5p; hsa-miR-221-3p; hsa-miR-122-5p; hsa-miR-125b-5p |
| Long QT Syndrome | 6.99e-8 | 3.14e-7 | 3.14e-7 | 0.263793 | 6 | hsa-miR-29b-3p; hsa-miR-148a-3p; hsa-miR-7-5p; hsa-miR-221-3p; hsa-miR-122-5p; hsa-miR-125b-5p |
| Multiple Endocrine Neoplasia Type 1 | 6.99e-8 | 3.14e-7 | 3.14e-7 | 0.263793 | 6 | hsa-miR-29b-3p; hsa-miR-148a-3p; hsa-miR-7-5p; hsa-miR-221-3p; hsa-miR-122-5p; hsa-miR-125b-5p |
| Myopia | 6.99e-8 | 3.14e-7 | 3.14e-7 | 0.263793 | 6 | hsa-miR-29b-3p; hsa-miR-148a-3p; hsa-miR-7-5p; hsa-miR-221-3p; hsa-miR-122-5p; hsa-miR-125b-5p |
| Papillary Thyroid Carcinoma | 7.09e-8 | 3.14e-7 | 3.14e-7 | 0.465517 | 7 | hsa-miR-375-3p; hsa-miR-29b-3p; hsa-miR-148a-3p; hsa-miR-7-5p; hsa-miR-221-3p; hsa-miR-122-5p; hsa-miR-125b-5p |
| Neuroblastoma | 7.72e-8 | 3.14e-7 | 3.14e-7 | 0.75 | 8 | hsa-miR-375-3p; hsa-miR-29b-3p; hsa-miR-148a-3p; hsa-miR-7-5p; hsa-miR-221-3p; hsa-miR-200c-3p; hsa-let-7e-5p; hsa-miR-125b-5p |
| Albuminuria | 7.89e-8 | 3.14e-7 | 3.14e-7 | 0.268966 | 6 | hsa-miR-29b-3p; hsa-miR-148a-3p; hsa-miR-7-5p; hsa-miR-221-3p; hsa-miR-122-5p; hsa-miR-125b-5p |
| Behcet's Disease | 7.89e-8 | 3.14e-7 | 3.14e-7 | 0.268966 | 6 | hsa-miR-29b-3p; hsa-miR-148a-3p; hsa-miR-7-5p; hsa-miR-221-3p; hsa-miR-122-5p; hsa-miR-125b-5p |
| Burns | 7.89e-8 | 3.14e-7 | 3.14e-7 | 0.268966 | 6 | hsa-miR-29b-3p; hsa-miR-148a-3p; hsa-miR-7-5p; hsa-miR-221-3p; hsa-miR-122-5p; hsa-miR-125b-5p |
| Cerebellar Neoplasms | 7.89e-8 | 3.14e-7 | 3.14e-7 | 0.268966 | 6 | hsa-miR-29b-3p; hsa-miR-148a-3p; hsa-miR-7-5p; hsa-miR-221-3p; hsa-miR-122-5p; hsa-miR-125b-5p |
| Colorectal Neoplasms, Hereditary Nonpolyposis | 7.89e-8 | 3.14e-7 | 3.14e-7 | 0.268966 | 6 | hsa-miR-29b-3p; hsa-miR-148a-3p; hsa-miR-7-5p; hsa-miR-221-3p; hsa-miR-122-5p; hsa-miR-125b-5p |
| Creutzfeldt-Jakob Disease | 7.89e-8 | 3.14e-7 | 3.14e-7 | 0.268966 | 6 | hsa-miR-29b-3p; hsa-miR-148a-3p; hsa-miR-7-5p; hsa-miR-221-3p; hsa-miR-122-5p; hsa-miR-125b-5p |
| Diabetes Complications | 7.89e-8 | 3.14e-7 | 3.14e-7 | 0.268966 | 6 | hsa-miR-29b-3p; hsa-miR-148a-3p; hsa-miR-7-5p; hsa-miR-221-3p; hsa-miR-122-5p; hsa-miR-125b-5p |
| Encephalomyelitis, Autoimmune, Experimental | 7.89e-8 | 3.14e-7 | 3.14e-7 | 0.268966 | 6 | hsa-miR-29b-3p; hsa-miR-148a-3p; hsa-miR-7-5p; hsa-miR-221-3p; hsa-miR-122-5p; hsa-miR-125b-5p |
| Erythropoiesis | 7.89e-8 | 3.14e-7 | 3.14e-7 | 0.268966 | 6 | hsa-miR-29b-3p; hsa-miR-148a-3p; hsa-miR-7-5p; hsa-miR-221-3p; hsa-miR-122-5p; hsa-miR-125b-5p |
| Fanconi Anemia | 7.89e-8 | 3.14e-7 | 3.14e-7 | 0.268966 | 6 | hsa-miR-29b-3p; hsa-miR-148a-3p; hsa-miR-7-5p; hsa-miR-221-3p; hsa-miR-122-5p; hsa-miR-125b-5p |
| Fibroblasts | 7.89e-8 | 3.14e-7 | 3.14e-7 | 0.268966 | 6 | hsa-miR-29b-3p; hsa-miR-148a-3p; hsa-miR-7-5p; hsa-miR-221-3p; hsa-miR-122-5p; hsa-miR-125b-5p |
| Francisella | 7.89e-8 | 3.14e-7 | 3.14e-7 | 0.268966 | 6 | hsa-miR-29b-3p; hsa-miR-148a-3p; hsa-miR-7-5p; hsa-miR-221-3p; hsa-miR-122-5p; hsa-miR-125b-5p |
| Gerstmann-Straussler-Scheinker Syndrome | 7.89e-8 | 3.14e-7 | 3.14e-7 | 0.268966 | 6 | hsa-miR-29b-3p; hsa-miR-148a-3p; hsa-miR-7-5p; hsa-miR-221-3p; hsa-miR-122-5p; hsa-miR-125b-5p |
| HEV | 7.89e-8 | 3.14e-7 | 3.14e-7 | 0.268966 | 6 | hsa-miR-29b-3p; hsa-miR-148a-3p; hsa-miR-7-5p; hsa-miR-221-3p; hsa-miR-122-5p; hsa-miR-125b-5p |
| Hearing Loss | 7.89e-8 | 3.14e-7 | 3.14e-7 | 0.268966 | 6 | hsa-miR-29b-3p; hsa-miR-148a-3p; hsa-miR-7-5p; hsa-miR-221-3p; hsa-miR-122-5p; hsa-miR-125b-5p |
| Hypoxia | 7.89e-8 | 3.14e-7 | 3.14e-7 | 0.268966 | 6 | hsa-miR-29b-3p; hsa-miR-148a-3p; hsa-miR-7-5p; hsa-miR-221-3p; hsa-miR-122-5p; hsa-miR-125b-5p |
| Intervertebral Disc | 7.89e-8 | 3.14e-7 | 3.14e-7 | 0.268966 | 6 | hsa-miR-29b-3p; hsa-miR-148a-3p; hsa-miR-7-5p; hsa-miR-221-3p; hsa-miR-122-5p; hsa-miR-125b-5p |
| Keloid | 7.89e-8 | 3.14e-7 | 3.14e-7 | 0.268966 | 6 | hsa-miR-29b-3p; hsa-miR-148a-3p; hsa-miR-7-5p; hsa-miR-221-3p; hsa-miR-122-5p; hsa-miR-125b-5p |
| Leukemia, Biphenotypic, Acute | 7.89e-8 | 3.14e-7 | 3.14e-7 | 0.268966 | 6 | hsa-miR-29b-3p; hsa-miR-148a-3p; hsa-miR-7-5p; hsa-miR-221-3p; hsa-miR-122-5p; hsa-miR-200c-3p |
| Leukemia, Myeloid, Chronic-Phase | 7.89e-8 | 3.14e-7 | 3.14e-7 | 0.268966 | 6 | hsa-miR-148a-3p; hsa-miR-7-5p; hsa-miR-221-3p; hsa-miR-122-5p; hsa-miR-200c-3p; hsa-miR-125b-5p |
| Leukoplakia, Oral | 7.89e-8 | 3.14e-7 | 3.14e-7 | 0.268966 | 6 | hsa-miR-29b-3p; hsa-miR-148a-3p; hsa-miR-7-5p; hsa-miR-221-3p; hsa-miR-122-5p; hsa-miR-125b-5p |
| Lichen Planus, Oral | 7.89e-8 | 3.14e-7 | 3.14e-7 | 0.268966 | 6 | hsa-miR-375-3p; hsa-miR-29b-3p; hsa-miR-148a-3p; hsa-miR-7-5p; hsa-miR-221-3p; hsa-miR-200c-3p |
| Liver Cirrhosis, Biliary | 7.89e-8 | 3.14e-7 | 3.14e-7 | 0.268966 | 6 | hsa-miR-29b-3p; hsa-miR-148a-3p; hsa-miR-7-5p; hsa-miR-221-3p; hsa-miR-122-5p; hsa-miR-125b-5p |
| Lymphoma, Extranodal NK-T-Cell | 7.89e-8 | 3.14e-7 | 3.14e-7 | 0.268966 | 6 | hsa-miR-29b-3p; hsa-miR-148a-3p; hsa-miR-7-5p; hsa-miR-221-3p; hsa-miR-122-5p; hsa-miR-125b-5p |
| Lymphoproliferative Disorders | 7.89e-8 | 3.14e-7 | 3.14e-7 | 0.268966 | 6 | hsa-miR-29b-3p; hsa-miR-148a-3p; hsa-miR-7-5p; hsa-miR-221-3p; hsa-miR-122-5p; hsa-miR-125b-5p |
| Myocardium | 7.89e-8 | 3.14e-7 | 3.14e-7 | 0.268966 | 6 | hsa-miR-29b-3p; hsa-miR-148a-3p; hsa-miR-221-3p; hsa-miR-122-5p; hsa-miR-200c-3p; hsa-miR-125b-5p |
| Odontogenic Tumors | 7.89e-8 | 3.14e-7 | 3.14e-7 | 0.268966 | 6 | hsa-miR-29b-3p; hsa-miR-148a-3p; hsa-miR-7-5p; hsa-miR-221-3p; hsa-miR-122-5p; hsa-miR-125b-5p |
| Osteolysis | 7.89e-8 | 3.14e-7 | 3.14e-7 | 0.268966 | 6 | hsa-miR-29b-3p; hsa-miR-148a-3p; hsa-miR-7-5p; hsa-miR-221-3p; hsa-miR-122-5p; hsa-miR-125b-5p |
| Pain | 7.89e-8 | 3.14e-7 | 3.14e-7 | 0.268966 | 6 | hsa-miR-29b-3p; hsa-miR-148a-3p; hsa-miR-7-5p; hsa-miR-221-3p; hsa-miR-200c-3p; hsa-miR-125b-5p |
| Patau Syndrome | 7.89e-8 | 3.14e-7 | 3.14e-7 | 0.268966 | 6 | hsa-miR-29b-3p; hsa-miR-148a-3p; hsa-miR-7-5p; hsa-miR-221-3p; hsa-miR-122-5p; hsa-miR-125b-5p |
| Precursor T-Cell Lymphoblastic Leukemia-Lymphoma | 7.89e-8 | 3.14e-7 | 3.14e-7 | 0.268966 | 6 | hsa-miR-29b-3p; hsa-miR-148a-3p; hsa-miR-7-5p; hsa-miR-221-3p; hsa-miR-122-5p; hsa-miR-125b-5p |
| RNA Virus Infections | 7.89e-8 | 3.14e-7 | 3.14e-7 | 0.268966 | 6 | hsa-miR-29b-3p; hsa-miR-148a-3p; hsa-miR-7-5p; hsa-miR-221-3p; hsa-miR-122-5p; hsa-miR-125b-5p |
| Radiation Injuries | 7.89e-8 | 3.14e-7 | 3.14e-7 | 0.268966 | 6 | hsa-miR-29b-3p; hsa-miR-148a-3p; hsa-miR-7-5p; hsa-miR-221-3p; hsa-miR-122-5p; hsa-miR-125b-5p |
| Renal Insufficiency | 7.89e-8 | 3.14e-7 | 3.14e-7 | 0.268966 | 6 | hsa-miR-29b-3p; hsa-miR-148a-3p; hsa-miR-7-5p; hsa-miR-221-3p; hsa-miR-122-5p; hsa-miR-125b-5p |
| Sjögren’s Syndrome | 7.89e-8 | 3.14e-7 | 3.14e-7 | 0.268966 | 6 | hsa-miR-29b-3p; hsa-miR-148a-3p; hsa-miR-7-5p; hsa-miR-221-3p; hsa-miR-122-5p; hsa-miR-125b-5p |
| Synapses | 7.89e-8 | 3.14e-7 | 3.14e-7 | 0.268966 | 6 | hsa-miR-29b-3p; hsa-miR-148a-3p; hsa-miR-7-5p; hsa-miR-221-3p; hsa-miR-122-5p; hsa-miR-125b-5p |
| Vascular Calcification | 7.89e-8 | 3.14e-7 | 3.14e-7 | 0.268966 | 6 | hsa-miR-29b-3p; hsa-miR-148a-3p; hsa-miR-7-5p; hsa-miR-221-3p; hsa-miR-122-5p; hsa-miR-125b-5p |
| Acoustic Neuroma | 7.89e-8 | 3.14e-7 | 3.14e-7 | 0.268966 | 6 | hsa-miR-29b-3p; hsa-miR-148a-3p; hsa-miR-7-5p; hsa-miR-221-3p; hsa-miR-122-5p; hsa-miR-125b-5p |
| Acute Leukemia | 7.89e-8 | 3.14e-7 | 3.14e-7 | 0.268966 | 6 | hsa-miR-29b-3p; hsa-miR-148a-3p; hsa-miR-7-5p; hsa-miR-221-3p; hsa-miR-122-5p; hsa-miR-125b-5p |
| Adrenal Cortex Cancer | 7.89e-8 | 3.14e-7 | 3.14e-7 | 0.268966 | 6 | hsa-miR-375-3p; hsa-miR-148a-3p; hsa-miR-7-5p; hsa-miR-221-3p; hsa-miR-200c-3p; hsa-miR-125b-5p |
| Adrenal Cortical Adenoma | 7.89e-8 | 3.14e-7 | 3.14e-7 | 0.268966 | 6 | hsa-miR-375-3p; hsa-miR-148a-3p; hsa-miR-7-5p; hsa-miR-221-3p; hsa-miR-200c-3p; hsa-miR-125b-5p |
| Alcoholic Hepatitis | 7.89e-8 | 3.14e-7 | 3.14e-7 | 0.268966 | 6 | hsa-miR-29b-3p; hsa-miR-148a-3p; hsa-miR-7-5p; hsa-miR-221-3p; hsa-miR-122-5p; hsa-miR-125b-5p |
| Anus Neoplasm | 7.89e-8 | 3.14e-7 | 3.14e-7 | 0.268966 | 6 | hsa-miR-29b-3p; hsa-miR-148a-3p; hsa-miR-7-5p; hsa-miR-221-3p; hsa-miR-122-5p; hsa-miR-125b-5p |
| Breast Ductal Carcinoma | 7.89e-8 | 3.14e-7 | 3.14e-7 | 0.268966 | 6 | hsa-miR-29b-3p; hsa-miR-148a-3p; hsa-miR-7-5p; hsa-miR-221-3p; hsa-miR-122-5p; hsa-miR-125b-5p |
| Chlamydia | 7.89e-8 | 3.14e-7 | 3.14e-7 | 0.268966 | 6 | hsa-miR-29b-3p; hsa-miR-148a-3p; hsa-miR-7-5p; hsa-miR-221-3p; hsa-miR-122-5p; hsa-miR-125b-5p |
| Chondrodysplasia Punctata | 7.89e-8 | 3.14e-7 | 3.14e-7 | 0.268966 | 6 | hsa-miR-29b-3p; hsa-miR-148a-3p; hsa-miR-7-5p; hsa-miR-221-3p; hsa-miR-122-5p; hsa-miR-125b-5p |
| Familial Hyperlipidemia | 7.89e-8 | 3.14e-7 | 3.14e-7 | 0.268966 | 6 | hsa-miR-29b-3p; hsa-miR-148a-3p; hsa-miR-7-5p; hsa-miR-221-3p; hsa-miR-122-5p; hsa-miR-125b-5p |
| Focal Epithelial Hyperplasia | 7.89e-8 | 3.14e-7 | 3.14e-7 | 0.268966 | 6 | hsa-miR-29b-3p; hsa-miR-148a-3p; hsa-miR-7-5p; hsa-miR-221-3p; hsa-miR-122-5p; hsa-miR-125b-5p |
| Germ Cell And Embryonal Cancer | 7.89e-8 | 3.14e-7 | 3.14e-7 | 0.268966 | 6 | hsa-miR-29b-3p; hsa-miR-148a-3p; hsa-miR-7-5p; hsa-miR-221-3p; hsa-miR-122-5p; hsa-miR-125b-5p |
| Granulosa Cell Tumor | 7.89e-8 | 3.14e-7 | 3.14e-7 | 0.268966 | 6 | hsa-miR-29b-3p; hsa-miR-148a-3p; hsa-miR-7-5p; hsa-miR-221-3p; hsa-miR-122-5p; hsa-miR-125b-5p |
| Hemangioma | 7.89e-8 | 3.14e-7 | 3.14e-7 | 0.268966 | 6 | hsa-miR-29b-3p; hsa-miR-148a-3p; hsa-miR-7-5p; hsa-miR-221-3p; hsa-miR-122-5p; hsa-miR-125b-5p |
| Leiomyosarcoma | 7.89e-8 | 3.14e-7 | 3.14e-7 | 0.268966 | 6 | hsa-miR-29b-3p; hsa-miR-148a-3p; hsa-miR-7-5p; hsa-miR-221-3p; hsa-miR-122-5p; hsa-miR-125b-5p |
| Localized Scleroderma | 7.89e-8 | 3.14e-7 | 3.14e-7 | 0.268966 | 6 | hsa-miR-29b-3p; hsa-miR-148a-3p; hsa-miR-7-5p; hsa-miR-221-3p; hsa-miR-122-5p; hsa-miR-125b-5p |
| Mycosis Fungoides | 7.89e-8 | 3.14e-7 | 3.14e-7 | 0.268966 | 6 | hsa-miR-29b-3p; hsa-miR-148a-3p; hsa-miR-7-5p; hsa-miR-221-3p; hsa-miR-200c-3p; hsa-miR-125b-5p |
| Myositis Ossificans | 7.89e-8 | 3.14e-7 | 3.14e-7 | 0.268966 | 6 | hsa-miR-29b-3p; hsa-miR-148a-3p; hsa-miR-7-5p; hsa-miR-221-3p; hsa-miR-122-5p; hsa-miR-125b-5p |
| Peripheral Nerve Sheath Neoplasm | 7.89e-8 | 3.14e-7 | 3.14e-7 | 0.268966 | 6 | hsa-miR-29b-3p; hsa-miR-148a-3p; hsa-miR-7-5p; hsa-miR-221-3p; hsa-miR-122-5p; hsa-miR-125b-5p |
| Psoriatic Arthritis | 7.89e-8 | 3.14e-7 | 3.14e-7 | 0.268966 | 6 | hsa-miR-29b-3p; hsa-miR-148a-3p; hsa-miR-7-5p; hsa-miR-221-3p; hsa-miR-122-5p; hsa-miR-125b-5p |
| Psychotic Disorder | 7.89e-8 | 3.14e-7 | 3.14e-7 | 0.268966 | 6 | hsa-miR-29b-3p; hsa-miR-148a-3p; hsa-miR-7-5p; hsa-miR-221-3p; hsa-miR-122-5p; hsa-miR-125b-5p |
| Chemical And Drug Induced Liver Injury | 8.87e-8 | 3.19e-7 | 3.19e-7 | 0.274138 | 6 | hsa-miR-29b-3p; hsa-miR-148a-3p; hsa-miR-7-5p; hsa-miR-221-3p; hsa-miR-122-5p; hsa-miR-125b-5p |
| Eye Abnormalities | 8.87e-8 | 3.19e-7 | 3.19e-7 | 0.274138 | 6 | hsa-miR-29b-3p; hsa-miR-148a-3p; hsa-miR-7-5p; hsa-miR-221-3p; hsa-miR-122-5p; hsa-miR-125b-5p |
| Gilles De La Tourette Syndrome | 8.87e-8 | 3.19e-7 | 3.19e-7 | 0.274138 | 6 | hsa-miR-29b-3p; hsa-miR-148a-3p; hsa-miR-7-5p; hsa-miR-221-3p; hsa-miR-122-5p; hsa-miR-125b-5p |
| Hypoxia-Ischemia, Brain | 8.87e-8 | 3.19e-7 | 3.19e-7 | 0.274138 | 6 | hsa-miR-29b-3p; hsa-miR-148a-3p; hsa-miR-7-5p; hsa-miR-221-3p; hsa-miR-122-5p; hsa-miR-125b-5p |
| Lymphoma, Primary Effusion | 8.87e-8 | 3.19e-7 | 3.19e-7 | 0.274138 | 6 | hsa-miR-375-3p; hsa-miR-29b-3p; hsa-miR-148a-3p; hsa-miR-221-3p; hsa-miR-200c-3p; hsa-miR-125b-5p |
| Precursor B-Cell Lymphoblastic Leukemia-Lymphoma | 8.87e-8 | 3.19e-7 | 3.19e-7 | 0.274138 | 6 | hsa-miR-375-3p; hsa-miR-29b-3p; hsa-miR-148a-3p; hsa-miR-7-5p; hsa-miR-221-3p; hsa-miR-200c-3p |
| Trophoblasts | 8.87e-8 | 3.19e-7 | 3.19e-7 | 0.274138 | 6 | hsa-miR-29b-3p; hsa-miR-148a-3p; hsa-miR-7-5p; hsa-miR-221-3p; hsa-miR-200c-3p; hsa-miR-125b-5p |
| Atrophic Gastritis | 8.87e-8 | 3.19e-7 | 3.19e-7 | 0.274138 | 6 | hsa-miR-29b-3p; hsa-miR-148a-3p; hsa-miR-7-5p; hsa-miR-221-3p; hsa-miR-122-5p; hsa-miR-125b-5p |
| Chronic Kidney Disease | 8.87e-8 | 3.19e-7 | 3.19e-7 | 0.274138 | 6 | hsa-miR-29b-3p; hsa-miR-148a-3p; hsa-miR-7-5p; hsa-miR-221-3p; hsa-miR-200c-3p; hsa-miR-125b-5p |
| Colitis | 8.87e-8 | 3.19e-7 | 3.19e-7 | 0.274138 | 6 | hsa-miR-29b-3p; hsa-miR-148a-3p; hsa-miR-7-5p; hsa-miR-221-3p; hsa-miR-122-5p; hsa-miR-125b-5p |
| Dyspepsia | 8.87e-8 | 3.19e-7 | 3.19e-7 | 0.274138 | 6 | hsa-miR-29b-3p; hsa-miR-148a-3p; hsa-miR-7-5p; hsa-miR-221-3p; hsa-miR-122-5p; hsa-miR-125b-5p |
| Embryonal Carcinoma | 8.87e-8 | 3.19e-7 | 3.19e-7 | 0.274138 | 6 | hsa-miR-29b-3p; hsa-miR-148a-3p; hsa-miR-7-5p; hsa-miR-221-3p; hsa-miR-122-5p; hsa-miR-125b-5p |
| Glomerulonephritis | 8.87e-8 | 3.19e-7 | 3.19e-7 | 0.274138 | 6 | hsa-miR-29b-3p; hsa-miR-148a-3p; hsa-miR-7-5p; hsa-miR-221-3p; hsa-miR-122-5p; hsa-miR-125b-5p |
| Hyperglycemia | 8.87e-8 | 3.19e-7 | 3.19e-7 | 0.274138 | 6 | hsa-miR-29b-3p; hsa-miR-148a-3p; hsa-miR-7-5p; hsa-miR-221-3p; hsa-miR-122-5p; hsa-miR-125b-5p |
| Hypopharynx Cancer | 8.87e-8 | 3.19e-7 | 3.19e-7 | 0.274138 | 6 | hsa-miR-29b-3p; hsa-miR-148a-3p; hsa-miR-7-5p; hsa-miR-221-3p; hsa-miR-122-5p; hsa-miR-125b-5p |
| Irritable Bowel Syndrome | 8.87e-8 | 3.19e-7 | 3.19e-7 | 0.274138 | 6 | hsa-miR-29b-3p; hsa-miR-148a-3p; hsa-miR-7-5p; hsa-miR-221-3p; hsa-miR-122-5p; hsa-miR-125b-5p |
| Keratoconus | 8.87e-8 | 3.19e-7 | 3.19e-7 | 0.274138 | 6 | hsa-miR-29b-3p; hsa-miR-148a-3p; hsa-miR-7-5p; hsa-miR-221-3p; hsa-miR-122-5p; hsa-miR-125b-5p |
| Schistosomiasis | 8.87e-8 | 3.19e-7 | 3.19e-7 | 0.274138 | 6 | hsa-miR-29b-3p; hsa-miR-148a-3p; hsa-miR-7-5p; hsa-miR-221-3p; hsa-miR-122-5p; hsa-miR-125b-5p |
| Head And Neck Cancer | 9.84e-8 | 3.36e-7 | 3.36e-7 | 1.14828 | 9 | hsa-miR-375-3p; hsa-miR-29b-3p; hsa-miR-148a-3p; hsa-miR-7-5p; hsa-miR-221-3p; hsa-miR-122-5p; hsa-miR-200c-3p; hsa-let-7e-5p; hsa-miR-125b-5p |
| Cowden Disease | 9.96e-8 | 3.36e-7 | 3.36e-7 | 0.27931 | 6 | hsa-miR-29b-3p; hsa-miR-148a-3p; hsa-miR-7-5p; hsa-miR-221-3p; hsa-miR-122-5p; hsa-miR-125b-5p |
| Lymphoma, Large-Cell, Anaplastic | 9.96e-8 | 3.36e-7 | 3.36e-7 | 0.27931 | 6 | hsa-miR-29b-3p; hsa-miR-148a-3p; hsa-miR-7-5p; hsa-miR-221-3p; hsa-miR-200c-3p; hsa-miR-125b-5p |
| Angiosarcoma | 9.96e-8 | 3.36e-7 | 3.36e-7 | 0.27931 | 6 | hsa-miR-29b-3p; hsa-miR-148a-3p; hsa-miR-7-5p; hsa-miR-221-3p; hsa-miR-200c-3p; hsa-miR-125b-5p |
| Cystic Fibrosis | 9.96e-8 | 3.36e-7 | 3.36e-7 | 0.27931 | 6 | hsa-miR-29b-3p; hsa-miR-148a-3p; hsa-miR-7-5p; hsa-miR-221-3p; hsa-miR-122-5p; hsa-miR-125b-5p |
| Diabetic Retinopathy | 9.96e-8 | 3.36e-7 | 3.36e-7 | 0.27931 | 6 | hsa-miR-29b-3p; hsa-miR-148a-3p; hsa-miR-7-5p; hsa-miR-221-3p; hsa-miR-122-5p; hsa-miR-125b-5p |
| Giant Cell Tumor | 9.96e-8 | 3.36e-7 | 3.36e-7 | 0.27931 | 6 | hsa-miR-29b-3p; hsa-miR-148a-3p; hsa-miR-7-5p; hsa-miR-221-3p; hsa-miR-122-5p; hsa-miR-125b-5p |
| Intermediate Coronary Syndrome | 9.96e-8 | 3.36e-7 | 3.36e-7 | 0.27931 | 6 | hsa-miR-29b-3p; hsa-miR-148a-3p; hsa-miR-7-5p; hsa-miR-221-3p; hsa-miR-122-5p; hsa-miR-125b-5p |
| Leprosy | 9.96e-8 | 3.36e-7 | 3.36e-7 | 0.27931 | 6 | hsa-miR-29b-3p; hsa-miR-148a-3p; hsa-miR-7-5p; hsa-miR-221-3p; hsa-miR-122-5p; hsa-miR-125b-5p |
| Myocarditis | 9.96e-8 | 3.36e-7 | 3.36e-7 | 0.27931 | 6 | hsa-miR-29b-3p; hsa-miR-148a-3p; hsa-miR-7-5p; hsa-miR-221-3p; hsa-miR-122-5p; hsa-miR-125b-5p |
| Neuroendocrine Carcinoma | 9.96e-8 | 3.36e-7 | 3.36e-7 | 0.27931 | 6 | hsa-miR-29b-3p; hsa-miR-148a-3p; hsa-miR-7-5p; hsa-miR-221-3p; hsa-miR-122-5p; hsa-miR-125b-5p |
| Systemic Mastocytosis | 9.96e-8 | 3.36e-7 | 3.36e-7 | 0.27931 | 6 | hsa-miR-29b-3p; hsa-miR-148a-3p; hsa-miR-7-5p; hsa-miR-221-3p; hsa-miR-122-5p; hsa-miR-125b-5p |
| Medulloblastoma | 1.05e-7 | 3.54e-7 | 3.54e-7 | 1.64483 | 10 | hsa-miR-375-3p; hsa-miR-29b-3p; hsa-miR-148a-3p; hsa-miR-216a-5p; hsa-miR-7-5p; hsa-miR-221-3p; hsa-miR-200c-3p; hsa-let-7e-5p; hsa-miR-125b-5p; hsa-miR-99b-5p |
| Musculoskeletal Abnormalities | 1.12e-7 | 3.63e-7 | 3.63e-7 | 0.284483 | 6 | hsa-miR-29b-3p; hsa-miR-148a-3p; hsa-miR-7-5p; hsa-miR-221-3p; hsa-miR-200c-3p; hsa-miR-125b-5p |
| Polycystic Kidney Diseases | 1.12e-7 | 3.63e-7 | 3.63e-7 | 0.284483 | 6 | hsa-miR-29b-3p; hsa-miR-148a-3p; hsa-miR-7-5p; hsa-miR-221-3p; hsa-miR-200c-3p; hsa-miR-125b-5p |
| Toxoplasma | 1.12e-7 | 3.63e-7 | 3.63e-7 | 0.284483 | 6 | hsa-miR-29b-3p; hsa-miR-148a-3p; hsa-miR-7-5p; hsa-miR-221-3p; hsa-miR-200c-3p; hsa-miR-125b-5p |
| Disease Of Metabolism | 1.12e-7 | 3.63e-7 | 3.63e-7 | 0.284483 | 6 | hsa-miR-29b-3p; hsa-miR-148a-3p; hsa-miR-7-5p; hsa-miR-221-3p; hsa-miR-122-5p; hsa-miR-125b-5p |
| Fibrosarcoma | 1.12e-7 | 3.63e-7 | 3.63e-7 | 0.284483 | 6 | hsa-miR-29b-3p; hsa-miR-148a-3p; hsa-miR-7-5p; hsa-miR-221-3p; hsa-miR-122-5p; hsa-miR-125b-5p |
| Hand, Foot And Mouth Disease | 1.12e-7 | 3.63e-7 | 3.63e-7 | 0.284483 | 6 | hsa-miR-375-3p; hsa-miR-148a-3p; hsa-miR-7-5p; hsa-miR-221-3p; hsa-miR-200c-3p; hsa-miR-125b-5p |
| Thyroid Carcinoma | 1.16e-7 | 3.76e-7 | 3.76e-7 | 2.30172 | 11 | hsa-miR-375-3p; hsa-miR-29b-3p; hsa-miR-148a-3p; hsa-miR-216a-5p; hsa-miR-7-5p; hsa-miR-221-3p; hsa-miR-320d; hsa-miR-200c-3p; hsa-let-7e-5p; hsa-miR-125b-5p; hsa-miR-99b-5p |
| Hepatitis C | 1.20e-7 | 3.86e-7 | 3.86e-7 | 0.501724 | 7 | hsa-miR-29b-3p; hsa-miR-148a-3p; hsa-miR-7-5p; hsa-miR-221-3p; hsa-miR-122-5p; hsa-miR-200c-3p; hsa-miR-125b-5p |
| Chronic Lymphocytic Leukemia | 1.25e-7 | 3.86e-7 | 3.86e-7 | 0.796552 | 8 | hsa-miR-375-3p; hsa-miR-29b-3p; hsa-miR-148a-3p; hsa-miR-7-5p; hsa-miR-221-3p; hsa-miR-122-5p; hsa-miR-200c-3p; hsa-miR-125b-5p |
| Graft Vs Host Disease | 1.25e-7 | 3.86e-7 | 3.86e-7 | 0.289655 | 6 | hsa-miR-29b-3p; hsa-miR-148a-3p; hsa-miR-7-5p; hsa-miR-221-3p; hsa-miR-122-5p; hsa-miR-125b-5p |
| Endometrial Carcinoma | 1.25e-7 | 3.86e-7 | 3.86e-7 | 0.289655 | 6 | hsa-miR-29b-3p; hsa-miR-148a-3p; hsa-miR-7-5p; hsa-miR-221-3p; hsa-miR-122-5p; hsa-miR-125b-5p |
| Lipid Metabolism Disorder | 1.25e-7 | 3.86e-7 | 3.86e-7 | 0.289655 | 6 | hsa-miR-29b-3p; hsa-miR-148a-3p; hsa-miR-7-5p; hsa-miR-221-3p; hsa-miR-122-5p; hsa-miR-125b-5p |
| Myasthenia Gravis | 1.25e-7 | 3.86e-7 | 3.86e-7 | 0.289655 | 6 | hsa-miR-29b-3p; hsa-miR-148a-3p; hsa-miR-7-5p; hsa-miR-221-3p; hsa-miR-122-5p; hsa-miR-125b-5p |
| Neutropenia | 1.25e-7 | 3.86e-7 | 3.86e-7 | 0.289655 | 6 | hsa-miR-29b-3p; hsa-miR-148a-3p; hsa-miR-7-5p; hsa-miR-221-3p; hsa-miR-122-5p; hsa-miR-125b-5p |
| Obesity | 1.25e-7 | 3.86e-7 | 3.86e-7 | 0.289655 | 6 | hsa-miR-29b-3p; hsa-miR-148a-3p; hsa-miR-7-5p; hsa-miR-221-3p; hsa-miR-200c-3p; hsa-miR-125b-5p |
| Vitiligo | 1.25e-7 | 3.86e-7 | 3.86e-7 | 0.289655 | 6 | hsa-miR-29b-3p; hsa-miR-148a-3p; hsa-miR-7-5p; hsa-miR-221-3p; hsa-miR-122-5p; hsa-miR-125b-5p |
| Sickle Cell Anemia | 1.39e-7 | 4.29e-7 | 4.29e-7 | 0.294828 | 6 | hsa-miR-29b-3p; hsa-miR-148a-3p; hsa-miR-7-5p; hsa-miR-221-3p; hsa-miR-122-5p; hsa-miR-125b-5p |
| HCV | 1.55e-7 | 4.68e-7 | 4.68e-7 | 0.3 | 6 | hsa-miR-29b-3p; hsa-miR-148a-3p; hsa-miR-7-5p; hsa-miR-221-3p; hsa-miR-122-5p; hsa-miR-125b-5p |
| Helplessness, Learned | 1.55e-7 | 4.68e-7 | 4.68e-7 | 0.3 | 6 | hsa-miR-375-3p; hsa-miR-148a-3p; hsa-miR-7-5p; hsa-miR-221-3p; hsa-miR-200c-3p; hsa-miR-125b-5p |
| Adult T-Cell Leukemia | 1.55e-7 | 4.68e-7 | 4.68e-7 | 0.3 | 6 | hsa-miR-29b-3p; hsa-miR-148a-3p; hsa-miR-7-5p; hsa-miR-221-3p; hsa-miR-200c-3p; hsa-miR-125b-5p |
| Muscular Dystrophy | 1.55e-7 | 4.68e-7 | 4.68e-7 | 0.3 | 6 | hsa-miR-375-3p; hsa-miR-29b-3p; hsa-miR-148a-3p; hsa-miR-221-3p; hsa-miR-200c-3p; hsa-miR-125b-5p |
| Endometrial Cancer | 1.60e-7 | 4.82e-7 | 4.82e-7 | 0.522414 | 7 | hsa-miR-375-3p; hsa-miR-29b-3p; hsa-miR-148a-3p; hsa-miR-7-5p; hsa-miR-221-3p; hsa-miR-200c-3p; hsa-miR-125b-5p |
| Hypertrophy | 1.72e-7 | 5.13e-7 | 5.13e-7 | 0.305172 | 6 | hsa-miR-375-3p; hsa-miR-29b-3p; hsa-miR-148a-3p; hsa-miR-221-3p; hsa-miR-200c-3p; hsa-miR-125b-5p |
| Abdominal Aortic Aneurysm | 1.72e-7 | 5.13e-7 | 5.13e-7 | 0.305172 | 6 | hsa-miR-29b-3p; hsa-miR-148a-3p; hsa-miR-7-5p; hsa-miR-221-3p; hsa-miR-122-5p; hsa-miR-125b-5p |
| Reperfusion Injury | 1.90e-7 | 5.64e-7 | 5.64e-7 | 0.310345 | 6 | hsa-miR-375-3p; hsa-miR-29b-3p; hsa-miR-148a-3p; hsa-miR-221-3p; hsa-miR-200c-3p; hsa-miR-125b-5p |
| Dermatitis | 1.90e-7 | 5.64e-7 | 5.64e-7 | 0.310345 | 6 | hsa-miR-29b-3p; hsa-miR-148a-3p; hsa-miR-7-5p; hsa-miR-221-3p; hsa-miR-122-5p; hsa-miR-125b-5p |
| Duchenne Muscular Dystrophy | 2.10e-7 | 6.10e-7 | 6.10e-7 | 0.543103 | 7 | hsa-miR-375-3p; hsa-miR-29b-3p; hsa-miR-148a-3p; hsa-miR-7-5p; hsa-miR-221-3p; hsa-miR-200c-3p; hsa-miR-125b-5p |
| Pancreatic Adenocarcinoma | 2.11e-7 | 6.10e-7 | 6.10e-7 | 2.43103 | 11 | hsa-miR-375-3p; hsa-miR-29b-3p; hsa-miR-148a-3p; hsa-miR-216a-5p; hsa-miR-7-5p; hsa-miR-221-3p; hsa-miR-320d; hsa-miR-122-5p; hsa-let-7e-5p; hsa-miR-125b-5p; hsa-miR-99b-5p |
| Brain Injuries | 2.11e-7 | 6.10e-7 | 6.10e-7 | 0.315517 | 6 | hsa-miR-29b-3p; hsa-miR-148a-3p; hsa-miR-7-5p; hsa-miR-221-3p; hsa-miR-122-5p; hsa-miR-125b-5p |
| Kaposi’s Sarcoma | 2.11e-7 | 6.10e-7 | 6.10e-7 | 0.315517 | 6 | hsa-miR-375-3p; hsa-miR-29b-3p; hsa-miR-148a-3p; hsa-miR-221-3p; hsa-miR-200c-3p; hsa-miR-125b-5p |
| Periodontitis | 2.11e-7 | 6.10e-7 | 6.10e-7 | 0.315517 | 6 | hsa-miR-29b-3p; hsa-miR-148a-3p; hsa-miR-7-5p; hsa-miR-221-3p; hsa-miR-200c-3p; hsa-miR-125b-5p |
| Cardiovascular Diseases | 2.33e-7 | 6.62e-7 | 6.62e-7 | 0.32069 | 6 | hsa-miR-29b-3p; hsa-miR-148a-3p; hsa-miR-7-5p; hsa-miR-221-3p; hsa-miR-122-5p; hsa-miR-125b-5p |
| Sepsis | 2.33e-7 | 6.62e-7 | 6.62e-7 | 0.32069 | 6 | hsa-miR-29b-3p; hsa-miR-148a-3p; hsa-miR-221-3p; hsa-miR-122-5p; hsa-miR-200c-3p; hsa-miR-125b-5p |
| Atrial Fibrillation | 2.33e-7 | 6.62e-7 | 6.62e-7 | 0.32069 | 6 | hsa-miR-29b-3p; hsa-miR-148a-3p; hsa-miR-7-5p; hsa-miR-221-3p; hsa-miR-200c-3p; hsa-miR-125b-5p |
| Mantle Cell Lymphoma | 2.33e-7 | 6.62e-7 | 6.62e-7 | 0.32069 | 6 | hsa-miR-29b-3p; hsa-miR-148a-3p; hsa-miR-7-5p; hsa-miR-221-3p; hsa-miR-200c-3p; hsa-miR-125b-5p |
| Atherosclerosis | 2.56e-7 | 7.21e-7 | 7.21e-7 | 0.558621 | 7 | hsa-miR-375-3p; hsa-miR-148a-3p; hsa-miR-7-5p; hsa-miR-221-3p; hsa-miR-200c-3p; hsa-miR-125b-5p; hsa-miR-99b-5p |
| Carotid Artery Disease | 2.57e-7 | 7.21e-7 | 7.21e-7 | 0.325862 | 6 | hsa-miR-29b-3p; hsa-miR-148a-3p; hsa-miR-7-5p; hsa-miR-221-3p; hsa-miR-122-5p; hsa-miR-125b-5p |
| Ectopic Pregnancy | 2.57e-7 | 7.21e-7 | 7.21e-7 | 0.325862 | 6 | hsa-miR-29b-3p; hsa-miR-148a-3p; hsa-miR-7-5p; hsa-miR-221-3p; hsa-miR-122-5p; hsa-miR-125b-5p |
| IgA Glomerulonephritis | 2.83e-7 | 7.87e-7 | 7.87e-7 | 0.331034 | 6 | hsa-miR-375-3p; hsa-miR-29b-3p; hsa-miR-148a-3p; hsa-miR-221-3p; hsa-miR-200c-3p; hsa-miR-125b-5p |
| Oligodendroglioma | 2.83e-7 | 7.87e-7 | 7.87e-7 | 0.331034 | 6 | hsa-miR-29b-3p; hsa-miR-148a-3p; hsa-miR-7-5p; hsa-miR-221-3p; hsa-miR-122-5p; hsa-miR-125b-5p |
| Squamous Cell Neoplasm | 3.11e-7 | 8.61e-7 | 8.61e-7 | 0.336207 | 6 | hsa-miR-29b-3p; hsa-miR-148a-3p; hsa-miR-7-5p; hsa-miR-221-3p; hsa-miR-200c-3p; hsa-miR-125b-5p |
| Urinary Bladder Cancer | 3.13e-7 | 8.63e-7 | 8.63e-7 | 0.894828 | 8 | hsa-miR-29b-3p; hsa-miR-148a-3p; hsa-miR-7-5p; hsa-miR-221-3p; hsa-miR-122-5p; hsa-miR-200c-3p; hsa-miR-125b-5p; hsa-miR-99b-5p |
| Laryngeal Neoplasms | 3.41e-7 | 9.33e-7 | 9.33e-7 | 0.341379 | 6 | hsa-miR-375-3p; hsa-miR-29b-3p; hsa-miR-148a-3p; hsa-miR-7-5p; hsa-miR-221-3p; hsa-miR-200c-3p |
| Myelodysplastic myeloproliferative Neoplasm | 3.41e-7 | 9.33e-7 | 9.33e-7 | 0.341379 | 6 | hsa-miR-375-3p; hsa-miR-29b-3p; hsa-miR-148a-3p; hsa-miR-7-5p; hsa-miR-221-3p; hsa-miR-200c-3p |
| Lung Cancer | 3.49e-7 | 9.52e-7 | 9.52e-7 | 3.49655 | 12 | hsa-miR-375-3p; hsa-miR-29b-3p; hsa-miR-148a-3p; hsa-miR-216a-5p; hsa-miR-7-5p; hsa-miR-221-3p; hsa-miR-320d; hsa-miR-122-5p; hsa-miR-200c-3p; hsa-let-7e-5p; hsa-miR-125b-5p; hsa-miR-99b-5p |
| Mesothelioma | 3.74e-7 | 1.01e-6 | 1.01e-6 | 0.346552 | 6 | hsa-miR-375-3p; hsa-miR-29b-3p; hsa-miR-148a-3p; hsa-miR-221-3p; hsa-miR-200c-3p; hsa-miR-125b-5p |
| Liver Cirrhosis | 4.09e-7 | 1.10e-6 | 1.10e-6 | 0.351724 | 6 | hsa-miR-375-3p; hsa-miR-148a-3p; hsa-miR-7-5p; hsa-miR-221-3p; hsa-miR-200c-3p; hsa-miR-125b-5p |
| Osteoarthritis | 4.47e-7 | 1.20e-6 | 1.20e-6 | 0.356897 | 6 | hsa-miR-29b-3p; hsa-miR-148a-3p; hsa-miR-221-3p; hsa-miR-122-5p; hsa-miR-200c-3p; hsa-miR-125b-5p |
| Chronic Obstructive Pulmonary Disease | 4.88e-7 | 1.31e-6 | 1.31e-6 | 0.362069 | 6 | hsa-miR-375-3p; hsa-miR-29b-3p; hsa-miR-148a-3p; hsa-miR-221-3p; hsa-miR-200c-3p; hsa-miR-125b-5p |
| Lupus Nephritis | 5.31e-7 | 1.42e-6 | 1.42e-6 | 0.367241 | 6 | hsa-miR-29b-3p; hsa-miR-7-5p; hsa-miR-221-3p; hsa-miR-122-5p; hsa-miR-200c-3p; hsa-miR-125b-5p |
| Brain Cancer | 5.78e-7 | 1.54e-6 | 1.54e-6 | 0.372414 | 6 | hsa-miR-29b-3p; hsa-miR-148a-3p; hsa-miR-7-5p; hsa-miR-221-3p; hsa-miR-200c-3p; hsa-miR-125b-5p |
| Basal Cell Carcinoma | 6.29e-7 | 1.66e-6 | 1.66e-6 | 0.377586 | 6 | hsa-miR-29b-3p; hsa-miR-148a-3p; hsa-miR-7-5p; hsa-miR-221-3p; hsa-miR-200c-3p; hsa-miR-125b-5p |
| Huntington’s Disease | 6.31e-7 | 1.66e-6 | 1.66e-6 | 1.41724 | 9 | hsa-miR-375-3p; hsa-miR-29b-3p; hsa-miR-148a-3p; hsa-miR-7-5p; hsa-miR-221-3p; hsa-miR-122-5p; hsa-miR-200c-3p; hsa-miR-125b-5p; hsa-miR-99b-5p |
| Myasthenia Gravis, Autoimmune, Experimental | 6.32e-7 | 1.66e-6 | 1.66e-6 | 0.636207 | 7 | hsa-miR-375-3p; hsa-miR-29b-3p; hsa-miR-221-3p; hsa-miR-320d; hsa-miR-122-5p; hsa-miR-200c-3p; hsa-miR-125b-5p |
| Breast Neoplasms | 6.43e-7 | 1.68e-6 | 1.68e-6 | 3.67759 | 12 | hsa-miR-375-3p; hsa-miR-29b-3p; hsa-miR-148a-3p; hsa-miR-216a-5p; hsa-miR-7-5p; hsa-miR-221-3p; hsa-miR-320d; hsa-miR-122-5p; hsa-miR-200c-3p; hsa-let-7e-5p; hsa-miR-125b-5p; hsa-miR-99b-5p |
| Pancreatic Ductal Adenocarcinoma | 7.39e-7 | 1.92e-6 | 1.92e-6 | 0.998276 | 8 | hsa-miR-375-3p; hsa-miR-148a-3p; hsa-miR-216a-5p; hsa-miR-221-3p; hsa-miR-320d; hsa-miR-122-5p; hsa-miR-200c-3p; hsa-miR-125b-5p |
| Graves Disease | 8.02e-7 | 2.08e-6 | 2.08e-6 | 0.393103 | 6 | hsa-miR-29b-3p; hsa-miR-148a-3p; hsa-miR-7-5p; hsa-miR-221-3p; hsa-miR-122-5p; hsa-miR-125b-5p |
| Pulmonary Tuberculosis | 8.68e-7 | 2.24e-6 | 2.24e-6 | 0.398276 | 6 | hsa-miR-29b-3p; hsa-miR-148a-3p; hsa-miR-7-5p; hsa-miR-221-3p; hsa-miR-200c-3p; hsa-miR-125b-5p |
| Heart Failure | 9.27e-7 | 2.39e-6 | 2.39e-6 | 0.672414 | 7 | hsa-miR-375-3p; hsa-miR-148a-3p; hsa-miR-216a-5p; hsa-miR-7-5p; hsa-miR-221-3p; hsa-miR-200c-3p; hsa-miR-125b-5p |
| Lung Small Cell Carcinoma | 9.77e-7 | 2.50e-6 | 2.50e-6 | 1.48966 | 9 | hsa-miR-375-3p; hsa-miR-29b-3p; hsa-miR-148a-3p; hsa-miR-216a-5p; hsa-miR-7-5p; hsa-miR-221-3p; hsa-miR-122-5p; hsa-miR-200c-3p; hsa-miR-125b-5p |
| Pituitary Adenoma | 1.04e-6 | 2.65e-6 | 2.65e-6 | 1.5 | 9 | hsa-miR-29b-3p; hsa-miR-148a-3p; hsa-miR-7-5p; hsa-miR-221-3p; hsa-miR-122-5p; hsa-miR-200c-3p; hsa-let-7e-5p; hsa-miR-125b-5p; hsa-miR-99b-5p |
| Multiple Sclerosis | 1.09e-6 | 2.76e-6 | 2.76e-6 | 0.413793 | 6 | hsa-miR-148a-3p; hsa-miR-7-5p; hsa-miR-221-3p; hsa-miR-122-5p; hsa-miR-200c-3p; hsa-miR-125b-5p |
| Myocardial Infarction | 1.09e-6 | 2.76e-6 | 2.76e-6 | 0.413793 | 6 | hsa-miR-375-3p; hsa-miR-148a-3p; hsa-miR-7-5p; hsa-miR-221-3p; hsa-miR-200c-3p; hsa-miR-125b-5p |
| Systemic Lupus Erythematosus | 1.09e-6 | 2.76e-6 | 2.76e-6 | 0.413793 | 6 | hsa-miR-375-3p; hsa-miR-148a-3p; hsa-miR-7-5p; hsa-miR-221-3p; hsa-miR-200c-3p; hsa-miR-125b-5p |
| Lung Squamous Cell Carcinoma | 1.14e-6 | 2.86e-6 | 2.86e-6 | 2.83966 | 11 | hsa-miR-375-3p; hsa-miR-29b-3p; hsa-miR-148a-3p; hsa-miR-7-5p; hsa-miR-221-3p; hsa-miR-320d; hsa-miR-122-5p; hsa-miR-200c-3p; hsa-let-7e-5p; hsa-miR-125b-5p; hsa-miR-99b-5p |
| Aids Dementia Complex | 1.27e-6 | 3.16e-6 | 3.16e-6 | 0.424138 | 6 | hsa-miR-29b-3p; hsa-miR-148a-3p; hsa-miR-7-5p; hsa-miR-221-3p; hsa-miR-122-5p; hsa-miR-125b-5p |
| Fragile X Syndrome | 1.27e-6 | 3.16e-6 | 3.16e-6 | 0.424138 | 6 | hsa-miR-29b-3p; hsa-miR-148a-3p; hsa-miR-7-5p; hsa-miR-221-3p; hsa-miR-122-5p; hsa-miR-125b-5p |
| Glioblastoma | 1.42e-6 | 3.52e-6 | 3.52e-6 | 3.92586 | 12 | hsa-miR-375-3p; hsa-miR-29b-3p; hsa-miR-148a-3p; hsa-miR-216a-5p; hsa-miR-7-5p; hsa-miR-221-3p; hsa-miR-320d; hsa-miR-122-5p; hsa-miR-200c-3p; hsa-let-7e-5p; hsa-miR-125b-5p; hsa-miR-99b-5p |
| Acute Myeloid Leukemia | 1.54e-6 | 3.81e-6 | 3.81e-6 | 1.09655 | 8 | hsa-miR-375-3p; hsa-miR-29b-3p; hsa-miR-148a-3p; hsa-miR-7-5p; hsa-miR-221-3p; hsa-miR-200c-3p; hsa-let-7e-5p; hsa-miR-125b-5p |
| Down Syndrome | 1.55e-6 | 3.81e-6 | 3.81e-6 | 0.724138 | 7 | hsa-miR-29b-3p; hsa-miR-148a-3p; hsa-miR-7-5p; hsa-miR-221-3p; hsa-miR-122-5p; hsa-miR-200c-3p; hsa-miR-125b-5p |
| Non-Alcoholic Fatty Liver Disease | 1.69e-6 | 4.14e-6 | 4.14e-6 | 0.444828 | 6 | hsa-miR-29b-3p; hsa-miR-7-5p; hsa-miR-221-3p; hsa-miR-122-5p; hsa-miR-200c-3p; hsa-miR-125b-5p |
| Melanoma | 1.88e-6 | 4.60e-6 | 4.60e-6 | 2.97414 | 11 | hsa-miR-375-3p; hsa-miR-29b-3p; hsa-miR-148a-3p; hsa-miR-216a-5p; hsa-miR-7-5p; hsa-miR-221-3p; hsa-miR-122-5p; hsa-miR-200c-3p; hsa-let-7e-5p; hsa-miR-125b-5p; hsa-miR-99b-5p |
| Oral Squamous Cell Carcinoma | 1.89e-6 | 4.60e-6 | 4.60e-6 | 2.20862 | 10 | hsa-miR-375-3p; hsa-miR-29b-3p; hsa-miR-148a-3p; hsa-miR-216a-5p; hsa-miR-7-5p; hsa-miR-221-3p; hsa-miR-122-5p; hsa-miR-200c-3p; hsa-let-7e-5p; hsa-miR-125b-5p |
| Carcinoma, Ductal, Breast | 1.97e-6 | 4.80e-6 | 4.80e-6 | 2.21897 | 10 | hsa-miR-375-3p; hsa-miR-29b-3p; hsa-miR-148a-3p; hsa-miR-7-5p; hsa-miR-221-3p; hsa-miR-320d; hsa-miR-200c-3p; hsa-let-7e-5p; hsa-miR-125b-5p; hsa-miR-99b-5p |
| Chronic Myeloid Leukemia | 2.07e-6 | 5.01e-6 | 5.01e-6 | 0.460345 | 6 | hsa-miR-375-3p; hsa-miR-29b-3p; hsa-miR-7-5p; hsa-miR-221-3p; hsa-miR-200c-3p; hsa-miR-125b-5p |
| Nevus, Pigmented | 2.18e-6 | 5.26e-6 | 5.26e-6 | 0.248276 | 5 | hsa-miR-29b-3p; hsa-miR-148a-3p; hsa-miR-7-5p; hsa-miR-221-3p; hsa-miR-122-5p |
| Osteosarcoma | 2.21e-6 | 5.30e-6 | 5.30e-6 | 2.24483 | 10 | hsa-miR-375-3p; hsa-miR-29b-3p; hsa-miR-148a-3p; hsa-miR-7-5p; hsa-miR-221-3p; hsa-miR-122-5p; hsa-miR-200c-3p; hsa-let-7e-5p; hsa-miR-125b-5p; hsa-miR-99b-5p |
| Cardiomegaly | 2.21e-6 | 5.30e-6 | 5.30e-6 | 0.465517 | 6 | hsa-miR-29b-3p; hsa-miR-148a-3p; hsa-miR-221-3p; hsa-miR-122-5p; hsa-miR-200c-3p; hsa-miR-125b-5p |
| Meningioma | 2.37e-6 | 5.66e-6 | 5.66e-6 | 0.77069 | 7 | hsa-miR-375-3p; hsa-miR-29b-3p; hsa-miR-148a-3p; hsa-miR-7-5p; hsa-miR-221-3p; hsa-miR-200c-3p; hsa-miR-125b-5p |
| Myotonic Dystrophy | 2.52e-6 | 6.00e-6 | 6.00e-6 | 0.475862 | 6 | hsa-miR-29b-3p; hsa-miR-148a-3p; hsa-miR-7-5p; hsa-miR-221-3p; hsa-miR-200c-3p; hsa-miR-125b-5p |
| Digestive System Neoplasms | 2.68e-6 | 6.31e-6 | 6.31e-6 | 0.258621 | 5 | hsa-miR-29b-3p; hsa-miR-148a-3p; hsa-miR-221-3p; hsa-miR-200c-3p; hsa-miR-125b-5p |
| Biliary Tract Neoplasm | 2.68e-6 | 6.31e-6 | 6.31e-6 | 0.258621 | 5 | hsa-miR-29b-3p; hsa-miR-148a-3p; hsa-miR-221-3p; hsa-miR-200c-3p; hsa-miR-125b-5p |
| Nervous System Disease | 2.68e-6 | 6.31e-6 | 6.31e-6 | 0.258621 | 5 | hsa-miR-148a-3p; hsa-miR-7-5p; hsa-miR-221-3p; hsa-miR-122-5p; hsa-miR-125b-5p |
| Ovarian Neoplasms | 2.88e-6 | 6.74e-6 | 6.74e-6 | 2.3069 | 10 | hsa-miR-375-3p; hsa-miR-29b-3p; hsa-miR-148a-3p; hsa-miR-216a-5p; hsa-miR-7-5p; hsa-miR-221-3p; hsa-miR-122-5p; hsa-miR-200c-3p; hsa-miR-125b-5p; hsa-miR-99b-5p |
| Acute Coronary Syndrome | 2.97e-6 | 6.79e-6 | 6.79e-6 | 0.263793 | 5 | hsa-miR-29b-3p; hsa-miR-148a-3p; hsa-miR-221-3p; hsa-miR-200c-3p; hsa-miR-125b-5p |
| Cicatrix | 2.97e-6 | 6.79e-6 | 6.79e-6 | 0.263793 | 5 | hsa-miR-148a-3p; hsa-miR-7-5p; hsa-miR-221-3p; hsa-miR-122-5p; hsa-miR-125b-5p |
| Hailey-Hailey Disease | 2.97e-6 | 6.79e-6 | 6.79e-6 | 0.263793 | 5 | hsa-miR-29b-3p; hsa-miR-148a-3p; hsa-miR-7-5p; hsa-miR-221-3p; hsa-miR-122-5p |
| Nasal Polyps | 2.97e-6 | 6.79e-6 | 6.79e-6 | 0.263793 | 5 | hsa-miR-29b-3p; hsa-miR-148a-3p; hsa-miR-7-5p; hsa-miR-221-3p; hsa-miR-122-5p |
| Neurofibromatosis 2 | 2.97e-6 | 6.79e-6 | 6.79e-6 | 0.263793 | 5 | hsa-miR-29b-3p; hsa-miR-148a-3p; hsa-miR-221-3p; hsa-miR-122-5p; hsa-miR-125b-5p |
| Acquired Immunodeficiency Syndrome | 2.97e-6 | 6.79e-6 | 6.79e-6 | 0.263793 | 5 | hsa-miR-148a-3p; hsa-miR-7-5p; hsa-miR-221-3p; hsa-miR-122-5p; hsa-miR-125b-5p |
| Adenoma | 2.97e-6 | 6.79e-6 | 6.79e-6 | 0.796552 | 7 | hsa-miR-29b-3p; hsa-miR-148a-3p; hsa-miR-7-5p; hsa-miR-221-3p; hsa-miR-122-5p; hsa-let-7e-5p; hsa-miR-125b-5p |
| Head And Neck Squamous Cell Carcinoma | 3.14e-6 | 7.15e-6 | 7.15e-6 | 2.32759 | 10 | hsa-miR-375-3p; hsa-miR-29b-3p; hsa-miR-148a-3p; hsa-miR-7-5p; hsa-miR-320d; hsa-miR-122-5p; hsa-miR-200c-3p; hsa-let-7e-5p; hsa-miR-125b-5p; hsa-miR-99b-5p |
| Retinal Degeneration | 3.25e-6 | 7.38e-6 | 7.38e-6 | 0.496552 | 6 | hsa-miR-375-3p; hsa-miR-29b-3p; hsa-miR-148a-3p; hsa-miR-221-3p; hsa-miR-200c-3p; hsa-miR-125b-5p |
| Carcinoma, Non-Small-Cell Lung | 3.31e-6 | 7.48e-6 | 7.48e-6 | 1.21034 | 8 | hsa-miR-375-3p; hsa-miR-29b-3p; hsa-miR-148a-3p; hsa-miR-7-5p; hsa-miR-221-3p; hsa-miR-122-5p; hsa-miR-200c-3p; hsa-miR-125b-5p |
| Leukemia, B-Cell | 3.61e-6 | 8.05e-6 | 8.05e-6 | 0.274138 | 5 | hsa-miR-29b-3p; hsa-miR-148a-3p; hsa-miR-7-5p; hsa-miR-221-3p; hsa-miR-200c-3p |
| Interstitial Lung Disease | 3.61e-6 | 8.05e-6 | 8.05e-6 | 0.274138 | 5 | hsa-miR-29b-3p; hsa-miR-7-5p; hsa-miR-221-3p; hsa-miR-200c-3p; hsa-miR-125b-5p |
| Thoracic Aortic Aneurysm | 3.61e-6 | 8.05e-6 | 8.05e-6 | 0.274138 | 5 | hsa-miR-148a-3p; hsa-miR-7-5p; hsa-miR-221-3p; hsa-miR-200c-3p; hsa-miR-125b-5p |
| Toxoplasmosis | 3.61e-6 | 8.05e-6 | 8.05e-6 | 0.274138 | 5 | hsa-miR-29b-3p; hsa-miR-7-5p; hsa-miR-221-3p; hsa-miR-200c-3p; hsa-miR-125b-5p |
| Asthma | 3.67e-6 | 8.17e-6 | 8.17e-6 | 0.506897 | 6 | hsa-miR-375-3p; hsa-miR-29b-3p; hsa-miR-148a-3p; hsa-miR-221-3p; hsa-miR-200c-3p; hsa-miR-125b-5p |
| Rhinitis, Allergic, Perennial | 3.97e-6 | 8.79e-6 | 8.79e-6 | 0.27931 | 5 | hsa-miR-29b-3p; hsa-miR-148a-3p; hsa-miR-221-3p; hsa-miR-200c-3p; hsa-miR-125b-5p |
| Nasopharynx Carcinoma | 4.09e-6 | 9.03e-6 | 9.03e-6 | 3.19655 | 11 | hsa-miR-375-3p; hsa-miR-29b-3p; hsa-miR-148a-3p; hsa-miR-216a-5p; hsa-miR-7-5p; hsa-miR-221-3p; hsa-miR-122-5p; hsa-miR-200c-3p; hsa-let-7e-5p; hsa-miR-125b-5p; hsa-miR-99b-5p |
| Hepatoblastoma | 4.35e-6 | 9.55e-6 | 9.55e-6 | 0.284483 | 5 | hsa-miR-29b-3p; hsa-miR-148a-3p; hsa-miR-221-3p; hsa-miR-200c-3p; hsa-miR-125b-5p |
| Leiomyoma | 4.35e-6 | 9.55e-6 | 9.55e-6 | 0.284483 | 5 | hsa-miR-375-3p; hsa-miR-7-5p; hsa-miR-221-3p; hsa-miR-200c-3p; hsa-miR-125b-5p |
| Rheumatoid Arthritis | 4.39e-6 | 9.60e-6 | 9.60e-6 | 0.522414 | 6 | hsa-miR-29b-3p; hsa-miR-148a-3p; hsa-miR-7-5p; hsa-miR-221-3p; hsa-miR-200c-3p; hsa-miR-125b-5p |
| Progesterone-Receptor Negative Breast Cancer | 4.46e-6 | 9.72e-6 | 9.72e-6 | 3.22241 | 11 | hsa-miR-375-3p; hsa-miR-29b-3p; hsa-miR-148a-3p; hsa-miR-7-5p; hsa-miR-221-3p; hsa-miR-320d; hsa-miR-122-5p; hsa-miR-200c-3p; hsa-let-7e-5p; hsa-miR-125b-5p; hsa-miR-99b-5p |
| Lymphoma, T-Cell | 4.77e-6 | 1.03e-5 | 1.03e-5 | 0.289655 | 5 | hsa-miR-29b-3p; hsa-miR-148a-3p; hsa-miR-7-5p; hsa-miR-221-3p; hsa-miR-200c-3p |
| Myocytes, Cardiac | 4.77e-6 | 1.03e-5 | 1.03e-5 | 0.289655 | 5 | hsa-miR-29b-3p; hsa-miR-7-5p; hsa-miR-221-3p; hsa-miR-200c-3p; hsa-miR-125b-5p |
| Liposarcoma | 4.95e-6 | 1.07e-5 | 1.07e-5 | 0.858621 | 7 | hsa-miR-375-3p; hsa-miR-29b-3p; hsa-miR-148a-3p; hsa-miR-221-3p; hsa-miR-122-5p; hsa-miR-200c-3p; hsa-miR-125b-5p |
| Myeloproliferative Disorders | 5.21e-6 | 1.10e-5 | 1.10e-5 | 0.294828 | 5 | hsa-miR-29b-3p; hsa-miR-148a-3p; hsa-miR-221-3p; hsa-miR-200c-3p; hsa-miR-125b-5p |
| Biliary Atresia | 5.21e-6 | 1.10e-5 | 1.10e-5 | 0.294828 | 5 | hsa-miR-148a-3p; hsa-miR-7-5p; hsa-miR-221-3p; hsa-miR-122-5p; hsa-miR-125b-5p |
| Hypertrophic Cardiomyopathy | 5.21e-6 | 1.10e-5 | 1.10e-5 | 0.294828 | 5 | hsa-miR-29b-3p; hsa-miR-148a-3p; hsa-miR-7-5p; hsa-miR-221-3p; hsa-miR-200c-3p |
| Small Cell Carcinoma | 5.21e-6 | 1.10e-5 | 1.10e-5 | 0.294828 | 5 | hsa-miR-375-3p; hsa-miR-148a-3p; hsa-miR-221-3p; hsa-miR-200c-3p; hsa-miR-125b-5p |
| Urinary Bladder Neoplasms | 5.22e-6 | 1.10e-5 | 1.10e-5 | 0.537931 | 6 | hsa-miR-375-3p; hsa-miR-148a-3p; hsa-miR-221-3p; hsa-miR-122-5p; hsa-miR-200c-3p; hsa-miR-99b-5p |
| Progressive Supranuclear Palsy | 5.22e-6 | 1.10e-5 | 1.10e-5 | 0.537931 | 6 | hsa-miR-29b-3p; hsa-miR-148a-3p; hsa-miR-7-5p; hsa-miR-221-3p; hsa-miR-122-5p; hsa-miR-125b-5p |
| Adrenocortical Carcinoma | 5.52e-6 | 1.17e-5 | 1.17e-5 | 0.543103 | 6 | hsa-miR-375-3p; hsa-miR-29b-3p; hsa-miR-7-5p; hsa-miR-320d; hsa-let-7e-5p; hsa-miR-125b-5p |
| Hematologic Cancer | 5.69e-6 | 1.18e-5 | 1.18e-5 | 0.3 | 5 | hsa-miR-29b-3p; hsa-miR-148a-3p; hsa-miR-7-5p; hsa-miR-221-3p; hsa-miR-200c-3p |
| Myeloid Leukemia | 5.69e-6 | 1.18e-5 | 1.18e-5 | 0.3 | 5 | hsa-miR-29b-3p; hsa-miR-148a-3p; hsa-miR-7-5p; hsa-miR-221-3p; hsa-miR-200c-3p |
| Neurilemmoma | 5.69e-6 | 1.18e-5 | 1.18e-5 | 0.3 | 5 | hsa-miR-29b-3p; hsa-miR-148a-3p; hsa-miR-221-3p; hsa-miR-122-5p; hsa-miR-125b-5p |
| Polycythemia Vera | 5.69e-6 | 1.18e-5 | 1.18e-5 | 0.3 | 5 | hsa-miR-29b-3p; hsa-miR-148a-3p; hsa-miR-221-3p; hsa-miR-200c-3p; hsa-miR-125b-5p |
| Kidney Cancer | 5.84e-6 | 1.21e-5 | 1.21e-5 | 0.548276 | 6 | hsa-miR-375-3p; hsa-miR-29b-3p; hsa-miR-221-3p; hsa-miR-122-5p; hsa-miR-200c-3p; hsa-miR-125b-5p |
| Atopic Dermatitis | 6.20e-6 | 1.27e-5 | 1.27e-5 | 0.305172 | 5 | hsa-miR-29b-3p; hsa-miR-148a-3p; hsa-miR-7-5p; hsa-miR-221-3p; hsa-miR-122-5p |
| Eosinophilic Esophagitis | 6.20e-6 | 1.27e-5 | 1.27e-5 | 0.305172 | 5 | hsa-miR-375-3p; hsa-miR-148a-3p; hsa-miR-221-3p; hsa-miR-200c-3p; hsa-miR-125b-5p |
| Systemic Scleroderma | 6.20e-6 | 1.27e-5 | 1.27e-5 | 0.305172 | 5 | hsa-miR-148a-3p; hsa-miR-7-5p; hsa-miR-221-3p; hsa-miR-200c-3p; hsa-miR-125b-5p |
| T-Cell Leukemia | 6.52e-6 | 1.34e-5 | 1.34e-5 | 0.558621 | 6 | hsa-miR-29b-3p; hsa-miR-148a-3p; hsa-miR-7-5p; hsa-miR-221-3p; hsa-let-7e-5p; hsa-miR-125b-5p |
| Azoospermia | 6.74e-6 | 1.38e-5 | 1.38e-5 | 0.310345 | 5 | hsa-miR-29b-3p; hsa-miR-148a-3p; hsa-miR-221-3p; hsa-miR-200c-3p; hsa-miR-125b-5p |
| Hodgkin’s Lymphoma | 7.29e-6 | 1.48e-5 | 1.48e-5 | 1.87759 | 9 | hsa-miR-29b-3p; hsa-miR-148a-3p; hsa-miR-216a-5p; hsa-miR-7-5p; hsa-miR-221-3p; hsa-miR-200c-3p; hsa-let-7e-5p; hsa-miR-125b-5p; hsa-miR-99b-5p |
| Autistic Disorder | 7.95e-6 | 1.61e-5 | 1.61e-5 | 0.32069 | 5 | hsa-miR-29b-3p; hsa-miR-148a-3p; hsa-miR-221-3p; hsa-miR-200c-3p; hsa-miR-125b-5p |
| Intracranial Hemorrhage, Hypertensive | 8.51e-6 | 1.72e-5 | 1.72e-5 | 0.584483 | 6 | hsa-miR-375-3p; hsa-miR-29b-3p; hsa-miR-221-3p; hsa-miR-122-5p; hsa-let-7e-5p; hsa-miR-99b-5p |
| Prostate Adenocarcinoma | 8.59e-6 | 1.73e-5 | 1.73e-5 | 1.91379 | 9 | hsa-miR-375-3p; hsa-miR-29b-3p; hsa-miR-148a-3p; hsa-miR-7-5p; hsa-miR-221-3p; hsa-miR-320d; hsa-miR-200c-3p; hsa-let-7e-5p; hsa-miR-99b-5p |
| Amyotrophic Lateral Sclerosis | 9.00e-6 | 1.81e-5 | 1.81e-5 | 1.92414 | 9 | hsa-miR-29b-3p; hsa-miR-148a-3p; hsa-miR-216a-5p; hsa-miR-7-5p; hsa-miR-221-3p; hsa-miR-122-5p; hsa-let-7e-5p; hsa-miR-125b-5p; hsa-miR-99b-5p |
| Acute Promyelocytic Leukemia | 9.32e-6 | 1.87e-5 | 1.87e-5 | 0.331034 | 5 | hsa-miR-375-3p; hsa-miR-29b-3p; hsa-miR-148a-3p; hsa-miR-221-3p; hsa-miR-200c-3p |
| HIV | 1.01e-5 | 2.01e-5 | 2.01e-5 | 0.336207 | 5 | hsa-miR-29b-3p; hsa-miR-148a-3p; hsa-miR-221-3p; hsa-miR-200c-3p; hsa-miR-125b-5p |
| Inflammatory Bowel Disease | 1.01e-5 | 2.01e-5 | 2.01e-5 | 0.336207 | 5 | hsa-miR-29b-3p; hsa-miR-7-5p; hsa-miR-221-3p; hsa-miR-200c-3p; hsa-miR-125b-5p |
| Wounds And Injuries | 1.26e-5 | 2.51e-5 | 2.51e-5 | 0.351724 | 5 | hsa-miR-29b-3p; hsa-miR-7-5p; hsa-miR-221-3p; hsa-miR-200c-3p; hsa-miR-125b-5p |
| Lung Adenocarcinoma | 1.40e-5 | 2.76e-5 | 2.76e-5 | 3.58448 | 11 | hsa-miR-375-3p; hsa-miR-29b-3p; hsa-miR-148a-3p; hsa-miR-7-5p; hsa-miR-221-3p; hsa-miR-320d; hsa-miR-122-5p; hsa-miR-200c-3p; hsa-let-7e-5p; hsa-miR-125b-5p; hsa-miR-99b-5p |
| Infection | 1.40e-5 | 2.76e-5 | 2.76e-5 | 0.636207 | 6 | hsa-miR-29b-3p; hsa-miR-148a-3p; hsa-miR-7-5p; hsa-miR-221-3p; hsa-miR-122-5p; hsa-miR-125b-5p |
| Intracranial Aneurysm | 1.67e-5 | 3.29e-5 | 3.29e-5 | 1.49483 | 8 | hsa-miR-29b-3p; hsa-miR-148a-3p; hsa-miR-7-5p; hsa-miR-221-3p; hsa-miR-320d; hsa-miR-122-5p; hsa-miR-125b-5p; hsa-miR-99b-5p |
| Rectum Cancer | 1.68e-5 | 3.29e-5 | 3.29e-5 | 0.372414 | 5 | hsa-miR-375-3p; hsa-miR-29b-3p; hsa-miR-148a-3p; hsa-miR-221-3p; hsa-miR-200c-3p |
| Ependymoma | 2.11e-5 | 4.13e-5 | 4.13e-5 | 0.682759 | 6 | hsa-miR-29b-3p; hsa-miR-148a-3p; hsa-miR-7-5p; hsa-miR-221-3p; hsa-miR-200c-3p; hsa-miR-125b-5p |
| Dilated Cardiomyopathy | 2.30e-5 | 4.50e-5 | 4.50e-5 | 0.693103 | 6 | hsa-miR-29b-3p; hsa-miR-148a-3p; hsa-miR-221-3p; hsa-miR-200c-3p; hsa-miR-125b-5p; hsa-miR-99b-5p |
| Relapsing-Remitting Multiple Sclerosis | 2.34e-5 | 4.56e-5 | 4.56e-5 | 2.15172 | 9 | hsa-miR-29b-3p; hsa-miR-148a-3p; hsa-miR-216a-5p; hsa-miR-7-5p; hsa-miR-221-3p; hsa-miR-320d; hsa-miR-200c-3p; hsa-miR-125b-5p; hsa-miR-99b-5p |
| Leukemia | 2.44e-5 | 4.73e-5 | 4.73e-5 | 3.77586 | 11 | hsa-miR-375-3p; hsa-miR-29b-3p; hsa-miR-148a-3p; hsa-miR-7-5p; hsa-miR-221-3p; hsa-miR-320d; hsa-miR-122-5p; hsa-miR-200c-3p; hsa-let-7e-5p; hsa-miR-125b-5p; hsa-miR-99b-5p |
| Autoimmune Lymphoproliferative Syndrome | 2.45e-5 | 4.73e-5 | 4.73e-5 | 0.0103448 | 2 | hsa-miR-7-5p; hsa-miR-125b-5p |
| Familial Mediterranean Fever | 2.45e-5 | 4.73e-5 | 4.73e-5 | 0.0103448 | 2 | hsa-miR-7-5p; hsa-miR-125b-5p |
| Uterine Cancer | 2.62e-5 | 5.04e-5 | 5.04e-5 | 0.708621 | 6 | hsa-miR-29b-3p; hsa-miR-148a-3p; hsa-miR-216a-5p; hsa-miR-221-3p; hsa-miR-122-5p; hsa-let-7e-5p |
| Prolactinoma | 2.65e-5 | 5.08e-5 | 5.08e-5 | 0.408621 | 5 | hsa-miR-29b-3p; hsa-miR-148a-3p; hsa-miR-7-5p; hsa-miR-221-3p; hsa-miR-200c-3p |
| Malignant Glioma | 2.78e-5 | 5.31e-5 | 5.31e-5 | 3.82241 | 11 | hsa-miR-375-3p; hsa-miR-29b-3p; hsa-miR-148a-3p; hsa-miR-216a-5p; hsa-miR-7-5p; hsa-miR-221-3p; hsa-miR-122-5p; hsa-miR-200c-3p; hsa-let-7e-5p; hsa-miR-125b-5p; hsa-miR-99b-5p |
| Chronic Leukemia | 2.94e-5 | 5.61e-5 | 5.61e-5 | 0.0672414 | 3 | hsa-miR-29b-3p; hsa-miR-7-5p; hsa-miR-125b-5p |
| Breast Cancer | 2.99e-5 | 5.68e-5 | 5.68e-5 | 5.05345 | 12 | hsa-miR-375-3p; hsa-miR-29b-3p; hsa-miR-148a-3p; hsa-miR-216a-5p; hsa-miR-7-5p; hsa-miR-221-3p; hsa-miR-320d; hsa-miR-122-5p; hsa-miR-200c-3p; hsa-let-7e-5p; hsa-miR-125b-5p; hsa-miR-99b-5p |
| Cervical Adenocarcinoma | 3.00e-5 | 5.68e-5 | 5.68e-5 | 0.418966 | 5 | hsa-miR-375-3p; hsa-miR-221-3p; hsa-miR-200c-3p; hsa-let-7e-5p; hsa-miR-125b-5p |
| Arthritis | 3.93e-5 | 7.44e-5 | 7.44e-5 | 1.16897 | 7 | hsa-miR-29b-3p; hsa-miR-148a-3p; hsa-miR-7-5p; hsa-miR-221-3p; hsa-miR-122-5p; hsa-miR-200c-3p; hsa-miR-125b-5p |
| Parkinson’s Disease | 4.29e-5 | 8.09e-5 | 8.09e-5 | 1.18448 | 7 | hsa-miR-375-3p; hsa-miR-29b-3p; hsa-miR-148a-3p; hsa-miR-7-5p; hsa-miR-221-3p; hsa-miR-200c-3p; hsa-miR-125b-5p |
| Renal Clear Cell Carcinoma | 4.56e-5 | 8.56e-5 | 8.56e-5 | 2.32759 | 9 | hsa-miR-375-3p; hsa-miR-29b-3p; hsa-miR-148a-3p; hsa-miR-320d; hsa-miR-122-5p; hsa-miR-200c-3p; hsa-let-7e-5p; hsa-miR-125b-5p; hsa-miR-99b-5p |
| Lupus Vulgaris | 5.02e-5 | 9.41e-5 | 9.41e-5 | 0.465517 | 5 | hsa-miR-29b-3p; hsa-miR-148a-3p; hsa-miR-221-3p; hsa-miR-200c-3p; hsa-miR-125b-5p |
| Colon Adenocarcinoma | 5.95e-5 | 1.11e-4 | 1.11e-4 | 1.76897 | 8 | hsa-miR-375-3p; hsa-miR-29b-3p; hsa-miR-148a-3p; hsa-miR-216a-5p; hsa-miR-200c-3p; hsa-let-7e-5p; hsa-miR-125b-5p; hsa-miR-99b-5p |
| Thymic Carcinoma | 7.34e-5 | 1.36e-4 | 1.36e-4 | 0.0155172 | 2 | hsa-miR-7-5p; hsa-miR-125b-5p |
| Thymoma | 7.34e-5 | 1.36e-4 | 1.36e-4 | 0.0155172 | 2 | hsa-miR-7-5p; hsa-miR-125b-5p |
| Duke C | 9.01e-5 | 1.67e-4 | 1.67e-4 | 0.263793 | 4 | hsa-miR-7-5p; hsa-miR-221-3p; hsa-let-7e-5p; hsa-miR-99b-5p |
| Schizophrenia | 9.36e-5 | 1.73e-4 | 1.73e-4 | 0.884483 | 6 | hsa-miR-29b-3p; hsa-miR-148a-3p; hsa-miR-7-5p; hsa-miR-221-3p; hsa-miR-200c-3p; hsa-miR-125b-5p |
| HPV | 9.79e-5 | 1.80e-4 | 1.80e-4 | 0.0982759 | 3 | hsa-miR-375-3p; hsa-miR-221-3p; hsa-miR-125b-5p |
| Tonsil Cancer | 1.00e-4 | 1.84e-4 | 1.84e-4 | 0.894828 | 6 | hsa-miR-375-3p; hsa-miR-29b-3p; hsa-miR-216a-5p; hsa-miR-7-5p; hsa-let-7e-5p; hsa-miR-125b-5p |
| Stroke, Lacunar | 1.07e-4 | 1.96e-4 | 1.96e-4 | 1.36034 | 7 | hsa-miR-29b-3p; hsa-miR-148a-3p; hsa-miR-7-5p; hsa-miR-221-3p; hsa-miR-320d; hsa-miR-122-5p; hsa-miR-125b-5p |
| ACTH-Secreting Pituitary Adenoma | 1.13e-4 | 2.07e-4 | 2.07e-4 | 0.27931 | 4 | hsa-miR-29b-3p; hsa-miR-221-3p; hsa-miR-200c-3p; hsa-miR-125b-5p |
| Hypertension | 1.18e-4 | 2.14e-4 | 2.14e-4 | 1.38103 | 7 | hsa-miR-29b-3p; hsa-miR-148a-3p; hsa-miR-7-5p; hsa-miR-221-3p; hsa-miR-122-5p; hsa-miR-200c-3p; hsa-miR-125b-5p |
| Lymphoma | 1.18e-4 | 2.14e-4 | 2.14e-4 | 2.6069 | 9 | hsa-miR-375-3p; hsa-miR-29b-3p; hsa-miR-148a-3p; hsa-miR-7-5p; hsa-miR-221-3p; hsa-miR-122-5p; hsa-miR-200c-3p; hsa-miR-125b-5p; hsa-miR-99b-5p |
| Hepatocellular Carcinoma | 1.20e-4 | 2.17e-4 | 2.17e-4 | 4.38621 | 11 | hsa-miR-375-3p; hsa-miR-29b-3p; hsa-miR-148a-3p; hsa-miR-216a-5p; hsa-miR-7-5p; hsa-miR-221-3p; hsa-miR-122-5p; hsa-miR-200c-3p; hsa-let-7e-5p; hsa-miR-125b-5p; hsa-miR-99b-5p |
| Lymphoma, Non-Hodgkin | 1.22e-4 | 2.19e-4 | 2.19e-4 | 0.558621 | 5 | hsa-miR-148a-3p; hsa-miR-221-3p; hsa-let-7e-5p; hsa-miR-125b-5p; hsa-miR-99b-5p |
| Fibrosis | 1.22e-4 | 2.19e-4 | 2.19e-4 | 0.284483 | 4 | hsa-miR-221-3p; hsa-miR-122-5p; hsa-miR-200c-3p; hsa-miR-125b-5p |
| Retinal Neovascularization | 1.22e-4 | 2.19e-4 | 2.19e-4 | 0.284483 | 4 | hsa-miR-148a-3p; hsa-miR-221-3p; hsa-miR-200c-3p; hsa-miR-125b-5p |
| Progesterone-Receptor Positive Breast Cancer | 1.30e-4 | 2.31e-4 | 2.31e-4 | 3.43966 | 10 | hsa-miR-375-3p; hsa-miR-29b-3p; hsa-miR-148a-3p; hsa-miR-7-5p; hsa-miR-320d; hsa-miR-122-5p; hsa-miR-200c-3p; hsa-let-7e-5p; hsa-miR-125b-5p; hsa-miR-99b-5p |
| Intellectual Disability | 1.30e-4 | 2.31e-4 | 2.31e-4 | 1.96552 | 8 | hsa-miR-29b-3p; hsa-miR-148a-3p; hsa-miR-7-5p; hsa-miR-221-3p; hsa-miR-122-5p; hsa-miR-200c-3p; hsa-miR-125b-5p; hsa-miR-99b-5p |
| Myocardial Ischemia | 1.31e-4 | 2.31e-4 | 2.31e-4 | 0.289655 | 4 | hsa-miR-29b-3p; hsa-miR-7-5p; hsa-miR-221-3p; hsa-miR-200c-3p |
| Ischemia | 1.31e-4 | 2.31e-4 | 2.31e-4 | 0.289655 | 4 | hsa-miR-148a-3p; hsa-miR-221-3p; hsa-miR-200c-3p; hsa-miR-125b-5p |
| Lymphoplasmacytic Lymphoma | 1.31e-4 | 2.31e-4 | 2.31e-4 | 0.289655 | 4 | hsa-miR-7-5p; hsa-miR-221-3p; hsa-miR-200c-3p; hsa-miR-125b-5p |
| Rhabdomyosarcoma | 1.31e-4 | 2.31e-4 | 2.31e-4 | 0.289655 | 4 | hsa-miR-7-5p; hsa-miR-221-3p; hsa-miR-200c-3p; hsa-miR-125b-5p |
| Ovarian Clear Cell Carcinoma | 1.58e-4 | 2.78e-4 | 2.78e-4 | 0.589655 | 5 | hsa-miR-375-3p; hsa-miR-29b-3p; hsa-miR-320d; hsa-miR-200c-3p; hsa-miR-125b-5p |
| Neoplasms | 1.71e-4 | 3.01e-4 | 3.01e-4 | 0.6 | 5 | hsa-miR-375-3p; hsa-miR-148a-3p; hsa-miR-221-3p; hsa-miR-122-5p; hsa-miR-200c-3p |
| Laryngeal Squamous Cell Carcinoma | 1.77e-4 | 3.10e-4 | 3.10e-4 | 0.118966 | 3 | hsa-miR-375-3p; hsa-miR-148a-3p; hsa-miR-125b-5p |
| Heart Disease | 1.90e-4 | 3.32e-4 | 3.32e-4 | 2.06897 | 8 | hsa-miR-375-3p; hsa-miR-29b-3p; hsa-miR-148a-3p; hsa-miR-7-5p; hsa-miR-221-3p; hsa-miR-122-5p; hsa-miR-200c-3p; hsa-miR-125b-5p |
| Liver Disease | 2.01e-4 | 3.51e-4 | 3.51e-4 | 0.62069 | 5 | hsa-miR-7-5p; hsa-miR-221-3p; hsa-miR-122-5p; hsa-miR-200c-3p; hsa-miR-125b-5p |
| Colorectal Cancer | 2.14e-4 | 3.72e-4 | 3.72e-4 | 5.94828 | 12 | hsa-miR-375-3p; hsa-miR-29b-3p; hsa-miR-148a-3p; hsa-miR-216a-5p; hsa-miR-7-5p; hsa-miR-221-3p; hsa-miR-320d; hsa-miR-122-5p; hsa-miR-200c-3p; hsa-let-7e-5p; hsa-miR-125b-5p; hsa-miR-99b-5p |
| Fibromyalgia | 2.28e-4 | 3.96e-4 | 3.96e-4 | 0.12931 | 3 | hsa-miR-7-5p; hsa-miR-125b-5p; hsa-miR-99b-5p |
| MPTP Poisoning | 2.43e-4 | 4.21e-4 | 4.21e-4 | 0.0258621 | 2 | hsa-miR-7-5p; hsa-miR-221-3p |
| Autosomal Recessive Limb-Girdle Muscular Dystrophy Type 2A | 2.49e-4 | 4.30e-4 | 4.30e-4 | 0.341379 | 4 | hsa-miR-29b-3p; hsa-miR-148a-3p; hsa-miR-221-3p; hsa-miR-99b-5p |
| Eye Disease | 2.59e-4 | 4.45e-4 | 4.45e-4 | 2.86552 | 9 | hsa-miR-375-3p; hsa-miR-29b-3p; hsa-miR-148a-3p; hsa-miR-216a-5p; hsa-miR-7-5p; hsa-miR-221-3p; hsa-miR-122-5p; hsa-miR-200c-3p; hsa-miR-125b-5p |
| Diabetic Nephropathies | 2.64e-4 | 4.54e-4 | 4.54e-4 | 0.346552 | 4 | hsa-miR-375-3p; hsa-miR-221-3p; hsa-miR-200c-3p; hsa-miR-125b-5p |
| Cholangiocarcinoma | 2.94e-4 | 5.03e-4 | 5.03e-4 | 3.75517 | 10 | hsa-miR-375-3p; hsa-miR-29b-3p; hsa-miR-148a-3p; hsa-miR-7-5p; hsa-miR-221-3p; hsa-miR-122-5p; hsa-miR-200c-3p; hsa-let-7e-5p; hsa-miR-125b-5p; hsa-miR-99b-5p |
| Myopathy | 3.06e-4 | 5.22e-4 | 5.22e-4 | 0.677586 | 5 | hsa-miR-29b-3p; hsa-miR-148a-3p; hsa-miR-7-5p; hsa-miR-200c-3p; hsa-miR-125b-5p |
| Esophageal Cancer | 3.19e-4 | 5.44e-4 | 5.44e-4 | 4.81552 | 11 | hsa-miR-375-3p; hsa-miR-29b-3p; hsa-miR-148a-3p; hsa-miR-216a-5p; hsa-miR-7-5p; hsa-miR-221-3p; hsa-miR-122-5p; hsa-miR-200c-3p; hsa-let-7e-5p; hsa-miR-125b-5p; hsa-miR-99b-5p |
| Myeloid Neoplasm | 3.22e-4 | 5.48e-4 | 5.48e-4 | 0.144828 | 3 | hsa-miR-29b-3p; hsa-miR-7-5p; hsa-miR-125b-5p |
| Peripheral Nervous System Diseases | 3.31e-4 | 5.60e-4 | 5.60e-4 | 0.367241 | 4 | hsa-miR-29b-3p; hsa-miR-148a-3p; hsa-miR-7-5p; hsa-miR-125b-5p |
| Nemaline Myopathy | 3.31e-4 | 5.60e-4 | 5.60e-4 | 0.367241 | 4 | hsa-miR-29b-3p; hsa-miR-148a-3p; hsa-miR-221-3p; hsa-miR-99b-5p |
| Astrocytoma | 4.15e-4 | 7.00e-4 | 7.00e-4 | 1.15345 | 6 | hsa-miR-29b-3p; hsa-miR-148a-3p; hsa-miR-7-5p; hsa-miR-221-3p; hsa-miR-200c-3p; hsa-miR-125b-5p |
| Her2-Receptor Positive Breast Cancer | 4.40e-4 | 7.40e-4 | 7.40e-4 | 3.0569 | 9 | hsa-miR-375-3p; hsa-miR-29b-3p; hsa-miR-216a-5p; hsa-miR-7-5p; hsa-miR-221-3p; hsa-miR-320d; hsa-miR-122-5p; hsa-miR-200c-3p; hsa-miR-125b-5p |
| Adenoviridae Infections | 4.54e-4 | 7.61e-4 | 7.61e-4 | 0.398276 | 4 | hsa-miR-29b-3p; hsa-miR-148a-3p; hsa-miR-221-3p; hsa-miR-200c-3p |
| Intrahepatic Cholangiocarcinoma | 4.77e-4 | 7.98e-4 | 7.98e-4 | 0.403448 | 4 | hsa-miR-148a-3p; hsa-miR-221-3p; hsa-miR-122-5p; hsa-miR-200c-3p |
| Pancreatic Cancer | 4.90e-4 | 8.18e-4 | 8.18e-4 | 6.37241 | 12 | hsa-miR-375-3p; hsa-miR-29b-3p; hsa-miR-148a-3p; hsa-miR-216a-5p; hsa-miR-7-5p; hsa-miR-221-3p; hsa-miR-320d; hsa-miR-122-5p; hsa-miR-200c-3p; hsa-let-7e-5p; hsa-miR-125b-5p; hsa-miR-99b-5p |
| Pre-Eclampsia | 5.78e-4 | 9.60e-4 | 9.60e-4 | 0.424138 | 4 | hsa-miR-148a-3p; hsa-miR-221-3p; hsa-miR-200c-3p; hsa-miR-125b-5p |
| Lymphoblastic Leukemia | 5.78e-4 | 9.60e-4 | 9.60e-4 | 0.175862 | 3 | hsa-miR-29b-3p; hsa-miR-7-5p; hsa-miR-125b-5p |
| Aortic Valve Disease | 6.75e-4 | 0.0011151 | 0.0011151 | 0.0413793 | 2 | hsa-miR-148a-3p; hsa-miR-125b-5p |
| Gastrointestinal Stromal Tumor | 6.75e-4 | 0.0011151 | 0.0011151 | 0.0413793 | 2 | hsa-miR-221-3p; hsa-miR-125b-5p |
| Temporal Lobe Epilepsy | 6.94e-4 | 0.0011435 | 0.0011435 | 0.806897 | 5 | hsa-miR-375-3p; hsa-miR-221-3p; hsa-miR-200c-3p; hsa-let-7e-5p; hsa-miR-125b-5p |
| Vascular Disease | 7.11e-4 | 0.0011679 | 0.001168 | 4.13276 | 10 | hsa-miR-375-3p; hsa-miR-29b-3p; hsa-miR-148a-3p; hsa-miR-216a-5p; hsa-miR-7-5p; hsa-miR-221-3p; hsa-miR-122-5p; hsa-miR-200c-3p; hsa-miR-125b-5p; hsa-miR-99b-5p |
| Leukopenia | 8.66e-4 | 0.0014153 | 0.0014153 | 0.0465517 | 2 | hsa-miR-7-5p; hsa-miR-125b-5p |
| Pneumonia | 8.66e-4 | 0.0014153 | 0.0014153 | 0.0465517 | 2 | hsa-miR-7-5p; hsa-miR-125b-5p |
| Nephroblastoma | 8.73e-4 | 0.001424 | 0.001424 | 3.32586 | 9 | hsa-miR-29b-3p; hsa-miR-148a-3p; hsa-miR-216a-5p; hsa-miR-221-3p; hsa-miR-320d; hsa-miR-200c-3p; hsa-let-7e-5p; hsa-miR-125b-5p; hsa-miR-99b-5p |
| Oropharynx Cancer | 0.0010789 | 0.0017553 | 0.0017553 | 0.0517241 | 2 | hsa-miR-375-3p; hsa-miR-200c-3p |
| Lung Disease | 0.0010856 | 0.0017617 | 0.0017617 | 1.95517 | 7 | hsa-miR-29b-3p; hsa-miR-148a-3p; hsa-miR-7-5p; hsa-miR-221-3p; hsa-miR-122-5p; hsa-miR-200c-3p; hsa-miR-125b-5p |
| Adrenocortical Carcinoma | 0.0012443 | 0.0020142 | 0.0020142 | 0.915517 | 5 | hsa-miR-375-3p; hsa-miR-29b-3p; hsa-miR-148a-3p; hsa-miR-221-3p; hsa-miR-200c-3p |
| Small Intestine Cancer | 0.0014167 | 0.0022878 | 0.0022878 | 0.237931 | 3 | hsa-miR-375-3p; hsa-miR-221-3p; hsa-miR-99b-5p |
| Gastric Lymphoma | 0.0015348 | 0.0024723 | 0.0024723 | 0.548276 | 4 | hsa-miR-221-3p; hsa-let-7e-5p; hsa-miR-125b-5p; hsa-miR-99b-5p |
| Brain Disease | 0.001555 | 0.0024987 | 0.0024987 | 3.57414 | 9 | hsa-miR-375-3p; hsa-miR-29b-3p; hsa-miR-148a-3p; hsa-miR-7-5p; hsa-miR-221-3p; hsa-miR-122-5p; hsa-miR-200c-3p; hsa-miR-125b-5p; hsa-miR-99b-5p |
| Carcinoma | 0.0018238 | 0.0029234 | 0.0029234 | 3.64655 | 9 | hsa-miR-375-3p; hsa-miR-29b-3p; hsa-miR-148a-3p; hsa-miR-7-5p; hsa-miR-221-3p; hsa-miR-320d; hsa-miR-122-5p; hsa-miR-200c-3p; hsa-miR-125b-5p |
| Gallbladder Carcinoma | 0.0018541 | 0.0029575 | 0.0029574 | 0.0672414 | 2 | hsa-miR-122-5p; hsa-miR-125b-5p |
| Medulloepithelioma | 0.0018541 | 0.0029575 | 0.0029574 | 0.0672414 | 2 | hsa-miR-216a-5p; hsa-miR-122-5p |
| Ovarian Epithelial Cancer | 0.0021418 | 0.0034072 | 0.0034072 | 0.274138 | 3 | hsa-miR-7-5p; hsa-miR-200c-3p; hsa-miR-125b-5p |
| Thyroid Carcinoma, Anaplastic | 0.0021569 | 0.0034072 | 0.0034072 | 0.0724138 | 2 | hsa-miR-221-3p; hsa-miR-125b-5p |
| Colorectal Adenoma | 0.0021569 | 0.0034072 | 0.0034072 | 0.0724138 | 2 | hsa-let-7e-5p; hsa-miR-125b-5p |
| Laryngeal Carcinoma | 0.0021569 | 0.0034072 | 0.0034072 | 0.0724138 | 2 | hsa-miR-29b-3p; hsa-miR-125b-5p |
| Neurodegenerative Disease | 0.0023004 | 0.0036252 | 0.0036252 | 3.75517 | 9 | hsa-miR-375-3p; hsa-miR-29b-3p; hsa-miR-148a-3p; hsa-miR-7-5p; hsa-miR-221-3p; hsa-miR-122-5p; hsa-miR-200c-3p; hsa-miR-125b-5p; hsa-miR-99b-5p |
| Thyroid Adenoma | 0.0024815 | 0.0039012 | 0.0039012 | 0.0775862 | 2 | hsa-miR-29b-3p; hsa-miR-125b-5p |
| Skin Melanoma | 0.0027028 | 0.0042389 | 0.0042389 | 1.08621 | 5 | hsa-miR-375-3p; hsa-miR-148a-3p; hsa-miR-320d; hsa-miR-200c-3p; hsa-miR-125b-5p |
| Cervical Squamous Cell Carcinoma | 0.002761 | 0.0043198 | 0.0043198 | 1.09138 | 5 | hsa-miR-375-3p; hsa-miR-148a-3p; hsa-miR-122-5p; hsa-miR-200c-3p; hsa-miR-125b-5p |
| Williams-Beuren Syndrome | 0.0028279 | 0.0044139 | 0.0044139 | 0.0827586 | 2 | hsa-miR-200c-3p; hsa-miR-125b-5p |
| Multiple Sclerosis, Chronic Progressive | 0.0028802 | 0.0044848 | 0.0044848 | 1.10172 | 5 | hsa-miR-29b-3p; hsa-miR-148a-3p; hsa-miR-7-5p; hsa-miR-221-3p; hsa-miR-200c-3p |
| Neuroendocrine Tumors | 0.0029203 | 0.0045259 | 0.0045258 | 0.305172 | 3 | hsa-miR-375-3p; hsa-miR-148a-3p; hsa-miR-7-5p |
| Autosomal Recessive Limb-Girdle Muscular Dystrophy Type 2B | 0.0029203 | 0.0045259 | 0.0045258 | 0.305172 | 3 | hsa-miR-148a-3p; hsa-miR-221-3p; hsa-miR-99b-5p |
| Rectum Adenocarcinoma | 0.0029717 | 0.0045946 | 0.0045946 | 1.66034 | 6 | hsa-miR-375-3p; hsa-miR-29b-3p; hsa-miR-148a-3p; hsa-miR-216a-5p; hsa-miR-125b-5p; hsa-miR-99b-5p |
| Bone Marrow Cancer | 0.0031957 | 0.0049292 | 0.0049292 | 0.087931 | 2 | hsa-miR-29b-3p; hsa-miR-7-5p |
| Cancer | 0.0037679 | 0.0057981 | 0.0057981 | 6.11379 | 11 | hsa-miR-375-3p; hsa-miR-29b-3p; hsa-miR-148a-3p; hsa-miR-216a-5p; hsa-miR-7-5p; hsa-miR-221-3p; hsa-miR-320d; hsa-miR-122-5p; hsa-miR-200c-3p; hsa-miR-125b-5p; hsa-miR-99b-5p |
| Dermatomyositis | 0.0039726 | 0.0060987 | 0.0060987 | 0.708621 | 4 | hsa-miR-148a-3p; hsa-miR-7-5p; hsa-miR-221-3p; hsa-miR-99b-5p |
| Lupus Erythematosus | 0.0039951 | 0.0061189 | 0.0061189 | 0.0982759 | 2 | hsa-miR-7-5p; hsa-miR-125b-5p |
| Acromegaly | 0.0042033 | 0.0064228 | 0.0064228 | 0.346552 | 3 | hsa-miR-200c-3p; hsa-miR-125b-5p; hsa-miR-99b-5p |
| Skin Disease | 0.0044572 | 0.0067949 | 0.0067949 | 1.21552 | 5 | hsa-miR-29b-3p; hsa-miR-148a-3p; hsa-miR-7-5p; hsa-miR-200c-3p; hsa-miR-125b-5p |
| Stomach Cancer | 0.0045426 | 0.006909 | 0.006909 | 7.66552 | 12 | hsa-miR-375-3p; hsa-miR-29b-3p; hsa-miR-148a-3p; hsa-miR-216a-5p; hsa-miR-7-5p; hsa-miR-221-3p; hsa-miR-320d; hsa-miR-122-5p; hsa-miR-200c-3p; hsa-let-7e-5p; hsa-miR-125b-5p; hsa-miR-99b-5p |
| Polymyositis | 0.0048781 | 0.0069892 | 0.0069892 | 0.108621 | 2 | hsa-miR-221-3p; hsa-miR-99b-5p |
| Liver Cancer | 0.0063561 | 0.0085708 | 0.0085708 | 0.124138 | 2 | hsa-miR-122-5p; hsa-let-7e-5p |
| Familiar Ovarian Carcinoma | 0.0065996 | 0.0088809 | 0.0088809 | 2.63276 | 7 | hsa-miR-148a-3p; hsa-miR-216a-5p; hsa-miR-320d; hsa-miR-122-5p; hsa-miR-200c-3p; hsa-let-7e-5p; hsa-miR-99b-5p |
| Gastrointestinal System Disease | 0.0074415 | 0.0099729 | 0.0099729 | 0.134483 | 2 | hsa-miR-375-3p; hsa-miR-148a-3p |
| Intestinal Disease | 0.0074415 | 0.0099729 | 0.0099729 | 0.134483 | 2 | hsa-miR-7-5p; hsa-miR-125b-5p |
| Autosomal Dominant Disease | 0.0086054 | 0.0115091 | 0.0115091 | 0.144828 | 2 | hsa-miR-200c-3p; hsa-miR-125b-5p |
| Gastric Adenocarcinoma | 0.0090937 | 0.0121373 | 0.0121373 | 2.06379 | 6 | hsa-miR-216a-5p; hsa-miR-7-5p; hsa-miR-221-3p; hsa-miR-200c-3p; hsa-let-7e-5p; hsa-miR-125b-5p |
| Diabetes Mellitus | 0.0098258 | 0.0130878 | 0.0130878 | 2.81897 | 7 | hsa-miR-375-3p; hsa-miR-29b-3p; hsa-miR-148a-3p; hsa-miR-7-5p; hsa-miR-221-3p; hsa-miR-200c-3p; hsa-miR-125b-5p |
| Cerebral Hemorrhage, Traumatic | 0.0098462 | 0.0130883 | 0.0130883 | 0.155172 | 2 | hsa-miR-29b-3p; hsa-miR-122-5p |
| Kidney Disease | 0.0099793 | 0.0131058 | 0.0131058 | 1.46379 | 5 | hsa-miR-29b-3p; hsa-miR-7-5p; hsa-miR-221-3p; hsa-miR-200c-3p; hsa-miR-125b-5p |
| Colon Cancer | 0.0103595 | 0.0131301 | 0.0131301 | 8.20862 | 12 | hsa-miR-375-3p; hsa-miR-29b-3p; hsa-miR-148a-3p; hsa-miR-216a-5p; hsa-miR-7-5p; hsa-miR-221-3p; hsa-miR-320d; hsa-miR-122-5p; hsa-miR-200c-3p; hsa-let-7e-5p; hsa-miR-125b-5p; hsa-miR-99b-5p |
| Growth Hormone-Secreting Pituitary Adenoma | 0.010861 | 0.0137391 | 0.0137391 | 0.486207 | 3 | hsa-miR-200c-3p; hsa-miR-125b-5p; hsa-miR-99b-5p |
| Primary Biliary Cirrhosis | 0.0111626 | 0.0140933 | 0.0140933 | 0.165517 | 2 | hsa-miR-29b-3p; hsa-miR-122-5p |
| Uterine Corpus Endometrial Carcinoma | 0.0123111 | 0.0155134 | 0.0155134 | 2.1931 | 6 | hsa-miR-375-3p; hsa-miR-29b-3p; hsa-miR-7-5p; hsa-miR-221-3p; hsa-miR-200c-3p; hsa-miR-125b-5p |
| Neuromyelitis Optica | 0.0125447 | 0.0157575 | 0.0157575 | 0.977586 | 4 | hsa-miR-375-3p; hsa-miR-29b-3p; hsa-miR-148a-3p; hsa-let-7e-5p |
| Oxyphilic Adenoma | 0.012553 | 0.0157575 | 0.0157575 | 0.175862 | 2 | hsa-miR-29b-3p; hsa-miR-221-3p |
| Liver Neoplasms | 0.0129815 | 0.0162642 | 0.0162642 | 3.78621 | 8 | hsa-miR-375-3p; hsa-miR-29b-3p; hsa-miR-148a-3p; hsa-miR-221-3p; hsa-miR-122-5p; hsa-miR-200c-3p; hsa-let-7e-5p; hsa-miR-125b-5p |
| Uterine Fibroid | 0.0147745 | 0.0184752 | 0.0184752 | 0.191379 | 2 | hsa-miR-29b-3p; hsa-miR-125b-5p |
| Sleep Deprivation | 0.0155505 | 0.0189739 | 0.0189739 | 0.196552 | 2 | hsa-miR-125b-5p; hsa-miR-99b-5p |
| Biliary Tract Cancer | 0.0163089 | 0.0198622 | 0.0198622 | 2.32241 | 6 | hsa-miR-221-3p; hsa-miR-320d; hsa-miR-122-5p; hsa-miR-200c-3p; hsa-let-7e-5p; hsa-miR-125b-5p |
| Machado-Joseph Disease | 0.0171548 | 0.0208536 | 0.0208536 | 0.206897 | 2 | hsa-miR-375-3p; hsa-miR-125b-5p |
| Papillary Renal Cell Carcinoma | 0.0175821 | 0.0213334 | 0.0213334 | 2.35862 | 6 | hsa-miR-375-3p; hsa-miR-29b-3p; hsa-miR-148a-3p; hsa-miR-200c-3p; hsa-let-7e-5p; hsa-miR-125b-5p |
| Testicular Germ Cell Cancer | 0.0206044 | 0.0244117 | 0.0244117 | 0.615517 | 3 | hsa-miR-375-3p; hsa-miR-29b-3p; hsa-miR-200c-3p |
| Large Cell Carcinoma | 0.0242446 | 0.0286726 | 0.0286726 | 0.248276 | 2 | hsa-miR-7-5p; hsa-miR-200c-3p |
| Large Cell Neuroendocrine Cancer | 0.0265885 | 0.0307224 | 0.0307224 | 0.677586 | 3 | hsa-miR-29b-3p; hsa-miR-7-5p; hsa-miR-200c-3p |
| Ovarian Carcinoma | 0.0291078 | 0.0335741 | 0.0335741 | 1.25172 | 4 | hsa-miR-29b-3p; hsa-miR-221-3p; hsa-miR-200c-3p; hsa-miR-125b-5p |
| Polycystic Ovary Syndrome | 0.0356313 | 0.0390381 | 0.0390381 | 0.305172 | 2 | hsa-miR-7-5p; hsa-miR-221-3p |
| Stomach Carcinoma | 0.0402065 | 0.0438982 | 0.0438982 | 0.325862 | 2 | hsa-miR-375-3p; hsa-miR-221-3p |
| Gout | 0.0425759 | 0.0460242 | 0.0460242 | 0.336207 | 2 | hsa-miR-200c-3p; hsa-miR-125b-5p |

**Supplementary Table 3. Information on normal healthy human donor**

| **S. No** | **Donor ID/ Specimen ID** | **Age** | **Gender** | **BMI** | **Any medication?** | **Specimen  collection year** | **Ethnicity** | **Source** |
| --- | --- | --- | --- | --- | --- | --- | --- | --- |
| 1 | 106692 | 47 | F | 24 | Birth control pills | 2019 |  | BPM core, BUMC |
| 2 | 159171 | 39.8 | M | 20.8 | None | 2019 |  | BPM core, BUMC |
| 3 | 419039 | 41 | F | 26.7 | Magnesium, Vitamin D | 2019 |  | BPM core, BUMC |
| 4 | 150969 | 40 | M | 23.1 | None | 2019 |  | BPM core, BUMC |
| 5 | 161538 | 48 | M | 24.2 | None | 2019 |  | BPM core, BUMC |
| 6 | 161515 | 54 | M | 26.4 | None | 2019 |  | BPM core, BUMC |
| 7 | 103375 | 51 | F | 20.4 | None | 2019 |  | BPM core, BUMC |
| 8 | 161356 | 31 | F | 26.52 | None | 2021 |  | BPM core, BUMC |
| 9 | 162616 | 26 | F | 25 | None | 2021 |  | BPM core, BUMC |
| 10 | 162362 | 31 | F | 24.1 | None | 2021 | Hispanic | BPM core, BUMC |
| 11 | 164396 | 29 | M | 28.4 | None | 2021 | Non-Hispanic | BPM core, BUMC |
| 12 | D8001Pk2 | 30 | M | 22.8 | None | 2016 | Non-Hispanic | iSpecimen |
| 13 | D8180Pk2 | 43 | M | 22.5 | None | 2016 | Non-Hispanic | iSpecimen |
| 14 | D8183Pk2 | 42 | F | 25.5 | None | 2016 | Non-Hispanic | iSpecimen |
| 15 | D8205Pk2 | 40 | F | 25.4 | None | 2016 | Non-Hispanic | iSpecimen |
| 16 | D8230Pk2 | 35 | F | 25.3 | None | 2016 | Non-Hispanic | iSpecimen |
| 17 | D8556Pk2 | 40 | M | 23.5 | None | 2017 | Non-Hispanic | iSpecimen |
| 18 | D8617Pk2 | 35 | F | 24.2 | None | 2017 | Non-Hispanic | iSpecimen |
| 19 | D8659Pk2 | 35 | M | 23.1 | None | 2017 | Non-Hispanic | iSpecimen |
